# Photoredox Driven Amide Synthesis from Tertiary Amines and Carboxylic Acids via C–N Bond Cleavage

**DOI:** 10.1021/acsomega.5c04718

**Published:** 2025-07-23

**Authors:** Merve Dogan, Irem Akgul, Marion H. Emmert, Ozgur Yilmaz

**Affiliations:** † Department of Chemistry, Faculty of Sciences, 52983 Mersin University, Mersin 33343, Turkey; ‡ Discovery Chemistry, 2793 MRL, Merck & Co. Inc., 770 Sumneytown Pike, West Point, Pennsylvania 19486, United States

## Abstract

This manuscript describes a visible-light-promoted, Ir-catalyzed, aerobic protocol for amide synthesis from tertiary amines and carboxylic acids. The reaction likely proceeds through α-C–H oxidation of the tertiary amine, followed by C–N bond cleavage via hydrolysis to the secondary amine intermediate. Concurrently, the additive CF_3_SO_2_Na reacts to activate the carboxylic acid via an acyl fluoride intermediate, which in turn reacts with the secondary amine to yield the product amides. The established conditions tolerate a wide variety of carboxylic acid substrates, including complex carboxylic acids. Furthermore, previously unreactive amine substrates such as *N*-methyl morpholine and tri-*n*-butyl amine are shown to react readily.

## Graphical abstract

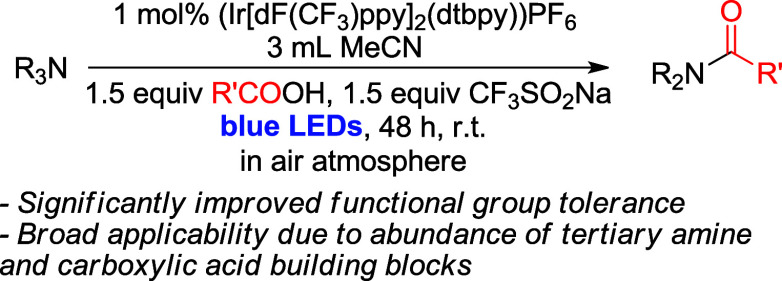

## Introduction

Amide substructures are essential building blocks across various industrial applications and can thus be found in diverse products such as plastics, agrochemicals, pharmaceuticals, and other biologically active molecules.^1–6^ Although there are several well-known classical strategies for their formation such as the Schmidt reaction,^7^ Staudinger reaction,^8^ and Ugi reaction^9^ as well as biocatalytic approaches,^10^ developing methods for the synthesis of amides is still of significant interest.^11,12^ This is particularly true, if the new approaches use uncommon building blocks leading to different types of disconnections or when novel reagents to activate carboxylic acids can be reported.

Traditionally, amides are synthesized via reaction of an acyl chloride (or other activated carboxylic acid) with a primary or secondary amine.^13^ Interestingly, recent developments allow also the use of tertiary amines as building blocks (Scheme 1A, Method A).^14,15^ These methods proceed through secondary amine intermediates, which are often produced *in situ* via oxidative pathways. Other unusual pathways to access amides from tertiary amines include the α-C-H oxidation of tertiary amines (Scheme 1A, Method B),^1,16^ and the reaction of tertiary amines with anhydrides (Scheme 1A, Method C).^17–19^ Relevant to the herein presented work, Pd­(II),^20^ Cu­(I)^6^ and photoredox catalyzed methods^21–23^ for the synthesis of amides via the reaction of tertiary amines with carboxylic acids have been reported (Scheme 1B). However, Cu­(I) and Pd­(II) catalyzed protocols require high temperatures (60–120 °C) and are not amenable to room temperature reactivity. On the other hand, known photoredox catalyzed methods are strongly limited with regard to the substrate scope they have been applied to.

**Scheme 1:**
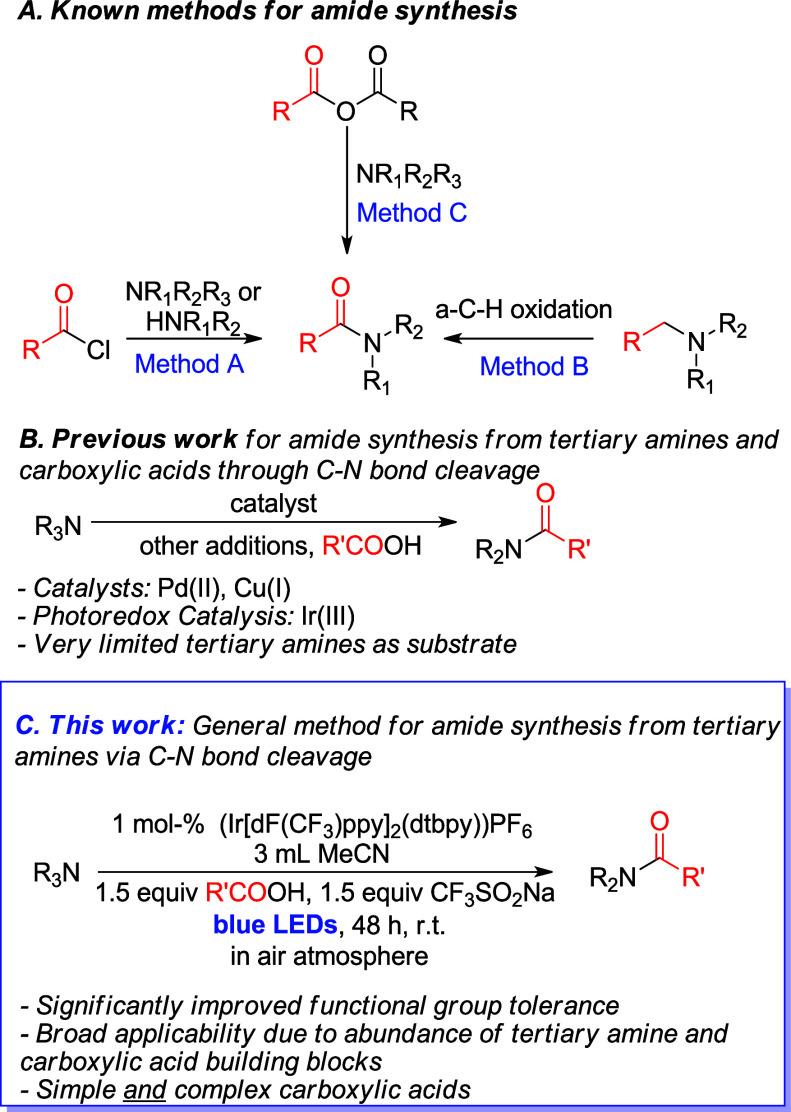
Previous Approaches and Herein Reported Protocol for Amide Synthesis from Tertiary Amines and Carboxylic Acids through C–N Bond Cleavage

Herein, we report a photoredox-catalyzed, general method for amide synthesis from tertiary amines and carboxylic acids. The reaction proceeds via C–H and C–N bond cleavage of the tertiary amine, which is an important and useful process^24–26^ enabled by air oxidation (Scheme 1C). Both simple and complex carboxylic acids have shown to be successful reactants under the described conditions. This work significantly broadens the functional group tolerance and thus the scope of useful building blocks for the direct synthesis of amides from tertiary amines and carboxylic acids, which in turn has the potential to allow access to novel chemical space.

## Results and Discussion

### Reaction Optimization

Reactions were optimized using NBu_3_ (tri-*n*-butylamine) as starting material. This compound is readily available in large quantities and has previously been shown to be relatively unreactive (6% *N*,*N*-dialkyl amide^23^ in direct amidation via C–N bond cleavage. Based on our previous studies on photoredox-catalyzed *N*-dealkylations of tertiary amines,^27^ we considered that (Ir­[dF­(CF_3_)­ppy]_2_(dtbpy))­PF_6_ in combination with blue LEDs (465 nm) would be a good starting point for a photoredox catalysis system. We reasoned that providing suitable conditions for the *N*-dealkylation of tertiary amines would be a key precondition to ensure the successful synthesis of amides, as this reaction has been proposed in the field as the first reaction step in direct amide formation from amines. We initiated the reaction optimization with acetic acid as acid source, but were initially not successful in obtaining the desired amine products (Table 1, entry 1). Screening several additives (NaOH, entry 2; TMSCF_3_, entry 3; for others, see the Supporting Information) did not result in successful reactivity; however, reactions in the presence of 1.0 equiv CF_3_SO_2_Na produced the target amide **1a** in 46% assay yield (entry 4). Interestingly, the product yield increased with larger amounts of CF_3_SO_2_Na (1.5 equiv) to 52% (entry 5), suggesting an activating role of this reagent.

**Table 1:**
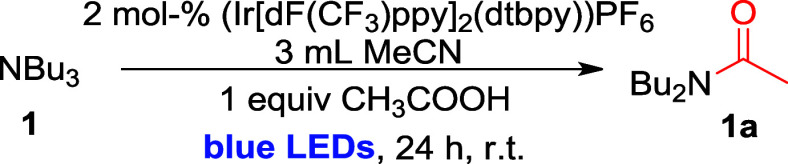
Selected Optimization Studies

Entry	Changes to Conditions^a^	Yield^b^
1	-	N.R.^c^
2	1 equiv NaOH	N.R.
3	1 equiv TMSCF_3_	N.R.
4	1 equiv CF_3_SO_2_Na	46%
5	1.5 equiv CF_3_SO_2_Na	52%
6	Ru(bpy)_3_	<5%
7	[Ir(dtbbpy)(ppy)_2_]PF_6_	9%
8	1.5 equiv CF_3_SO_2_Na, 1.5 equiv CH_3_COOH	86%
9	Entry 8 with 1 mol % [Ir]	85%
10	Entry 8 with 1 mol % [Ir] and 48 h	94%

a Conditions unless otherwise defined in the table: NBu_3_ (0.27 mmol, 64 μL, 1.0 equiv), 3 mL MeCN, (Ir­[dF­(CF_3_)­ppy]_2_(dtbpy))­PF_6_ (0.0054 mmol, 0.06 g, 0.02 equiv), CH_3_COOH (0.27 mmol, 16 μL, 1.0 equiv), r.t., 24 h, blue LEDs.

b Yields were determined by quantitative, crude ^1^H NMR using 1,3-dinitrobenzene or *p*-xylene as internal standard or by GC using decane as internal standard.

c N.R.: no reaction.

In a next step, we evaluated different photoredox catalysts (Ru­(bpy)_3_, [Ir­(dtbbpy)­(ppy)_2_]­PF_6_), which were found to lead to decreased yields (entries 6 and 7; < 5% and 9% yield, respectively). Additionally, a solvent screen was performed, which also did not result in a desired yield increase (see Supporting Information for details).

Excitingly, however, increasing both acid and CF_3_SO_2_Na loading to 1.5 equiv resulted in a significant jump in yield to afford 86% of desired amide (entry 8). Further reaction optimization focused on minimizing the amount of Ir catalyst. 1 mol % and 0.5 mol % Ir catalyst (for details, see the Supporting Information) afforded the desired product in 85% (Table 1, entry 9) and 64% yields, respectively. Finally, a time study (for details, see the Supporting Information) revealed that decreasing the reaction time also decreases the yield; in turn, increasing the reaction time led to an almost quantitative yield of desired product (94%, entry 10).

### Substrate Scope I: Reactions of Simple Tertiary Amines with AcOH

After successful optimization of the reaction conditions, we aimed to show that the obtained catalytic system would be useful for amide synthesis from different simple tertiary amines. Excitingly, amide products of nonactivated tertiary amines (Scheme 2) were readily obtained: All simple aliphatic amines (NEt_3_, **2**; N­(*n*-Pr)_3_, **3**; N­(*n*-Pent)_3_, **4**; N­(*n*-Hex)_3_, **5**; and N­(*n*-Oct)_3_, **6**) provided similarly high assay yields as obtained for the initial test substrate NBu_3_ **1** (92% **2a**, 89% **3a**, 88% **4a**, 91% **5a**, and 90% **6a**).

**Scheme 2:**
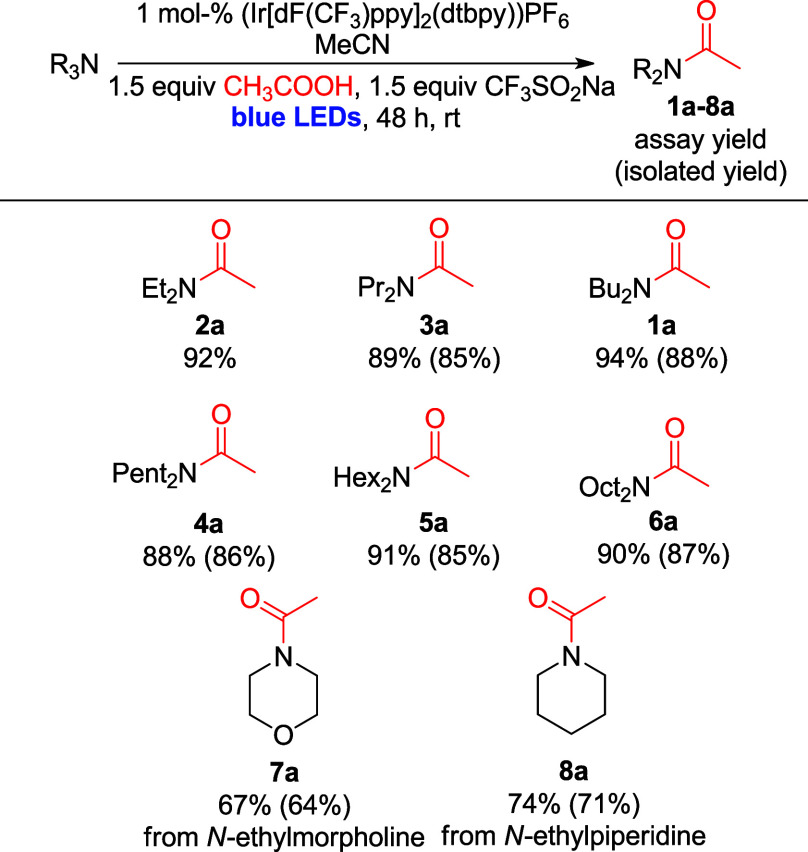
Substrate Scope I: Reaction of Simple Tertiary Amines with Acetic Acid^a^

a Assay yields (determined by quantitative ^1^H NMR of the crude reaction mixture or calibrated GC/FID in the presence of an internal standard) are shown outside of brackets; isolated yields are shown in brackets.

The synthesis of amide molecules from morpholine and piperidine derivatives, which are frequently found in the skeleton of biologically active molecules,^28,29^ was also tested. Amide products of the model substrates *N*-ethyl morpholine (**7**) and *N*-ethyl piperidine (**8**) were formed in 67% and 74% yield, respectively. Importantly, this is likely the first report of amide synthesis from *N*-alkyl morpholines, which has been shown to be unreactive with previous methods, including photoredox-catalyzed protocols.^22^

### Substrate Scope II: Reactivity of Simple Tertiary Amines with Propionic Acid

After determining that the developed protocol is able to react acetic acids with several tertiary amines, we aimed to show that the generality with regard to amine substrates remains true with other acids. To this purpose, reactions were carried out with propionic acid instead of AcOH, using the same tertiary amine molecule set used previously. Excitingly, amide products of all amines (Scheme 3) were readily obtained, affording yields between 44 and 71% assay yield (49% to 68% isolated yield). Overall, these yields are slightly lower than those obtained with AcOH, suggesting that the identify of the acid is important for the overall reaction performance. Notably, no desired product was obtained with *N*-ethyl morpholine.

**Scheme 3:**
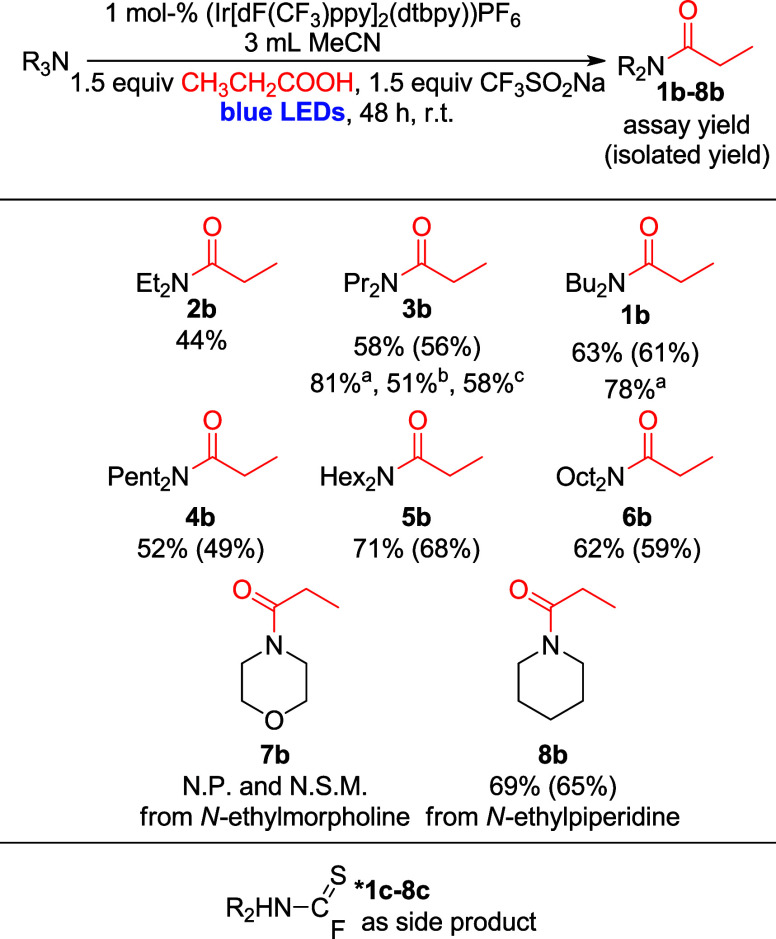
Substrate Scope II: Reaction of Simple Tertiary Amines with Propionic Acid^b^ ^c^ ^d^ ^e^ [Fn sch3-fn6]

a With 3 equiv CH_3_CH_2_COOH.
b With 3 equiv CF_3_SO_2_Na.
c With 3 equiv CH_3_CH_2_COOH and 3 equiv CF_3_SO_2_Na.
d Assay yields (determined by quantitative ^1^H NMR of the crude reaction mixture or calibrated GC/FID in the presence of an internal standard) are shown outside of brackets; isolated yields are shown in brackets.
e *See Supporting Information for yields.

One of the reasons for this decrease in yield is likely the formation of byproducts with the general formula R_2_N–C­(=S)­F (**1c** to **8c**; Scheme 3, bottom). These types of byproducts are known in the literature^30,31^ to originate from reduction of CF_3_SO_2_Na, producing F_3_CS^–^, which in turn forms F_2_C=S by elimination of fluoride. F_2_C=S can then react with secondary amines present in the solution to form the observed side products. Byproducts **1c**–**8c** were detected by GCMS and NMR in all reactions regardless of the acid used; isolation of **1c**–**8c** as pure materials and ^1^H, ^13^C and ^19^F NMR analysis supports the identity of the structures. The amount of byproduct formed varies with the acid used: while byproducts are formed in very low amounts in reactions with AcOH, they are obtained in yields between 24% and 46% in reactions with propanoic acid under standard conditions (see Supporting Information for details). Typically, an increase of these byproducts concurrently leads to a decrease of the target amide yields.

We reasoned that limiting the formation of side product could be achieved by further optimization of the reaction conditions. To this end, we varied the amounts of propanoic acid and CF_3_SO_2_Na used in reactions with NPr_3_ (**3**) (acid: 1 to 3 equiv; CF_3_SO_2_Na: 1 to 3 equiv). Excitingly, increasing the amount of acid relative to CF_3_SO_2_Na was found to significantly increase the yield of the amide product **3b** (from 52% to 81% with 1 equiv propanoic acid and 3 equiv CF_3_SO_2_Na; see Figure 1).

**Figure 1:**
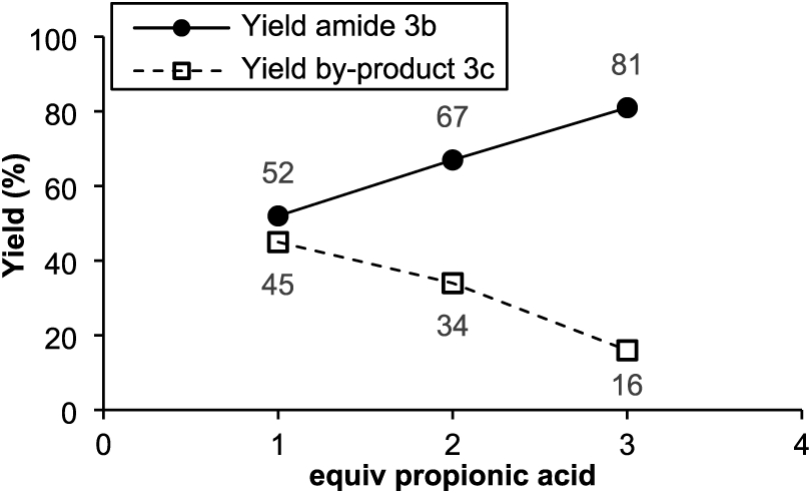
Yield of desired amide **3b** and by-product **3c** in dependence on propanoic acid loading. Conditions: NPr_3_ (0.27 mmol, 50 μL, 1.0 equiv), 3 mL MeCN, (Ir­[dF­(CF_3_)­ppy]_2_(dtbpy))­PF_6_ (0.0027 mmol, 0.003 g, 0.01 equiv), CF_3_SO_2_Na (0.41 mmol, 0.063 g, 1.5 equiv), CH_3_CH_2_COOH (1.0–3.0 equiv), r.t., 48 h, blue LEDs.

### Substrate Scope III: Reaction of Simple Tertiary Amines with (*S*)-(+)-Ibuprofen

Due to the just documented dependence of amide yields on the acid, we were particularly interested to explore how the suite of amine substrates would perform in combination with a more complex carboxylic acid. To this end, we chose (*S*)-(+)-Ibuprofen (Scheme 4) as test substrate. Excitingly, amide products of all tertiary amines were obtained in good to moderate yields (51% to 74% assay yield, 52% to 70% isolated yield), even with *N*-ethyl morpholine (**7d**, 77% assay yield, 74% isolated yield). Compared to reactions with propanoic acid, lower amounts of byproducts (**1c**–**8c**) were obtained (in yields between 21% and 43%).

**Scheme 4:**
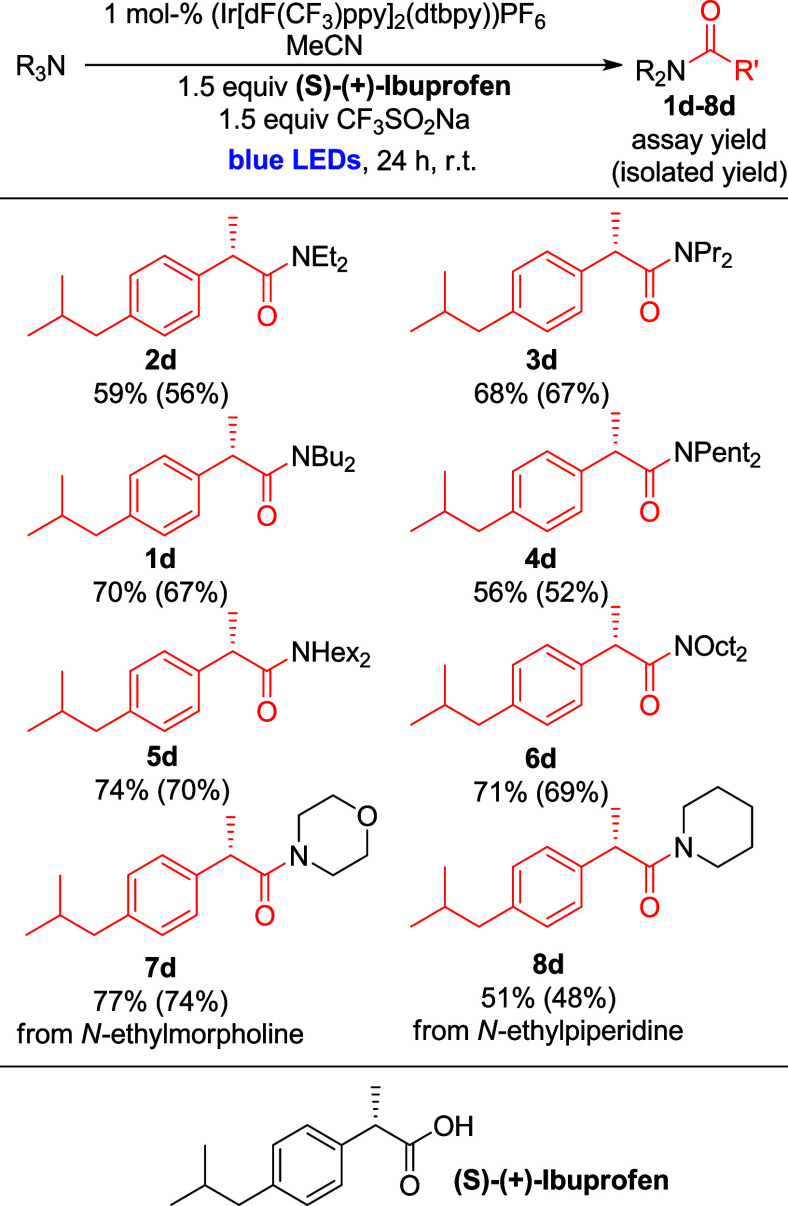
Substrate Scope III: Reaction of Simple Tertiary Amines with (*S*)-(+)-Ibuprofen[Fn sch4-fn7]

a Assay yields (determined by quantitative ^1^H NMR of the crude reaction mixture or calibrated GC/FID in the presence of an internal standard) are shown outside of brackets; isolated yields are shown in brackets.

### Substrate Scope IV: Other Acids

Encouraged by the general reactivity of (*S*)-(+)-Ibuprofen, we decided to explore further acid substrate to obtain insight into their suitability for the developed reaction. For the simple acids formic acid (**5**) and benzoic acid (**6**), reactions were performed with NBu_3_ (**1**) as amine substrate. Gratifyingly, the desired amides were obtaied in reasonable yields (74% and 57% assay yield, respectively; Scheme 5).

**Scheme 5:**
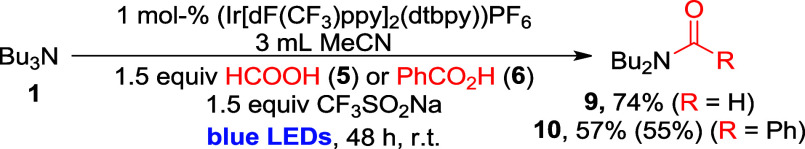
Reaction of NBu_3_(1) with Formic Acid or Benzoic Acid[Fn sch5-fn8]

a Assay yields (determined by quantitative ^1^H NMR of the crude reaction mixture) are shown outside of brackets; isolated yields are shown in brackets.

Next, a series of carboxylic acids of intermediate complexity were obtained from our building block collection. These substrates were subjected to the optimized reaction conditions and analyzed by UPLC-MS. For these reactions, slightly more forcing conditions (3.0 equiv CF_3_SO_2_Na) and an extended reaction time of 72 h were employed. Furthermore, we decided to employ *N*-methyl morpholine as a pharmaceutically relevant building block and to access chemical space that is not accessible with previous literature methods.

Results are shown as LCAP (liquid chromatography area percent); structural assignments were made based on ESI-MS data from the UPLCMS measurement (see Supporting Information for complete data set, including unreactive substrates and inconslusive reactivity). Excitingly, successful reactivity (9 to 73 LCAP) was observed with a range of htereocyclic substructures, including purine-bases (11a), pyrazoles (**13/24a**), annulated azoles (**14/16a**), pyridines (**15/20/21a**), indoles (**17a**), imidazoles (**19a**), and thioazoles (**20a**). Furthermore, a ketone substructure (**18a**) is tolerated, as well as other amide functionalities (**22a**). Finally, an electron-rich benzoic acid (**23a**) also shows reactivity (Scheme 6). This set of substrates shows that the developed reactivity is applicable to more complex substrates than simple amines or acids. Even though some LCAP values obtained were relatively low, no optimization was performed on these substrates, suggesting that the used reaction conditions are fairly general for aliphatic or aromatic acid reactants.

**Scheme 6:**
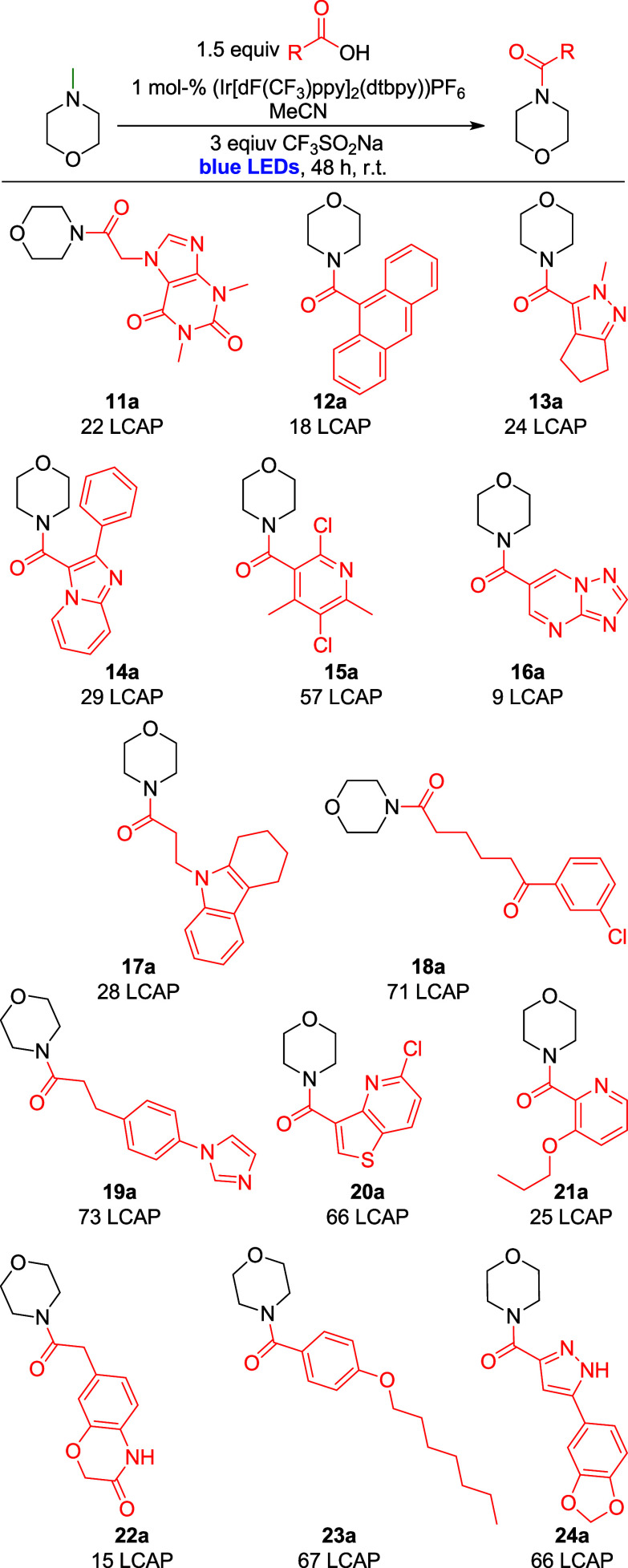
Complex Carboxylic Acids Reactions with *N*-Methylmorpholine[Fn sch6-fn9]

a LCAP (liquid chromatography area percent) values are determined by integration of the UPLC trace at 254 nm. Structural assignments were confirmed by online MS (for details see the Supporting Information).

### Proposed Mechanism and Supporting Experimental Studies

Our mechanistic investigations started with a series of background experiments were performed to further elucidate the role of each reaction component (Table 2). As expected, no amide product was obtained in the reactions performed without photoredox catalyst (entry 2), CF_3_SO_2_Na (entry 3), CH_3_COOH (entry 4) or light (entry 9). Combined with the observation that the product yield decreased by ∼50% when 0.5 equiv CF_3_SO_2_Na was used instead of 1 equiv (entry 5), these data support that CF_3_SO_2_Na drives product formation. Additional studies showed that the product yields decrease slightly in the presence of radical scavengers, such as 50 mol % BHT (82%; entry 6) or 50 mol % TEMPO (84%; entry 7). When the reaction was repeated using 3 equiv TEMPO (entry 8), no product was obtained. Setting up the reaction after flushing the reaction solution and headspace with nitrogen for 10 min resulted in a slightly lower reaction performance (56% yield; entry 10). This implies that air oxygen may play an active role in the formation of the desired product. Overall, these data suggest that radicals are important intermediates in the reaction mechanism.

**Table 2:**
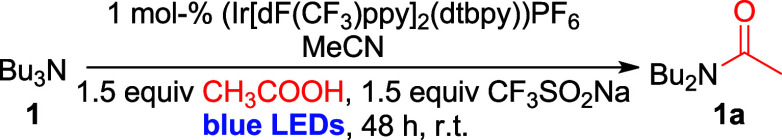
Control Reactions with NBu_3_

Entry	Conditions	Yield^a^
1	-	94%
2	No (Ir[dF(CF_3_)ppy]_2_(dtbpy))PF_6_	N.R.^b^
3	No CF_3_SO_2_Na	N.P.^c^
4	No CH_3_COOH	N.R.
5	With 0.5 equiv CF_3_SO_2_Na	38%
6	50 mol % BHT	82%
7	50 mol % TEMPO	84%
8	3 equiv TEMPO	N.R.
9	No light	N.R.
10	N_2_ atmosphere	56%

a Yields were determined by quantitative, crude ^1^H NMR using *p*-xylene as internal standard or by GC using decane as internal standard.

b N.R.: no reaction.

c N.P.: no product (**1a**), obtained 45% dealkylation product (HNBu_2_).

Considered together, the importance of radical intermediates, photocatalyst, and air suggests that the initial activation of the amine substrates occurs via an aerobically driven, single-electron oxidation of the amine substrate by the Ir photocatalyst (Scheme 7A), as proposed previously^27^ for N-dealkylation reactions. A second oxidation can then afford an iminium intermediate, which can be hydrolyzed in the presence of traces of water. Such a hydrolysis process would form an aldehyde product and a seconday amine HNR_2_. While the secondary amines are expected to further react to form the desired amides (see further below), the fate of the aldehyde products remains largely unclear, as aldehydes themselves have not been detected directly in the reactions at hand via GCMS, LCMS, or NMR analysis. However, an acid catalyzed dimerization of the aldehyde side products could lead to Claisen condensation products derived from aldehydes; those types of products (see Scheme 8A) were indeed observed by GCMS, supporting the formation of aldehydes in the reaction.

**Scheme 7:**
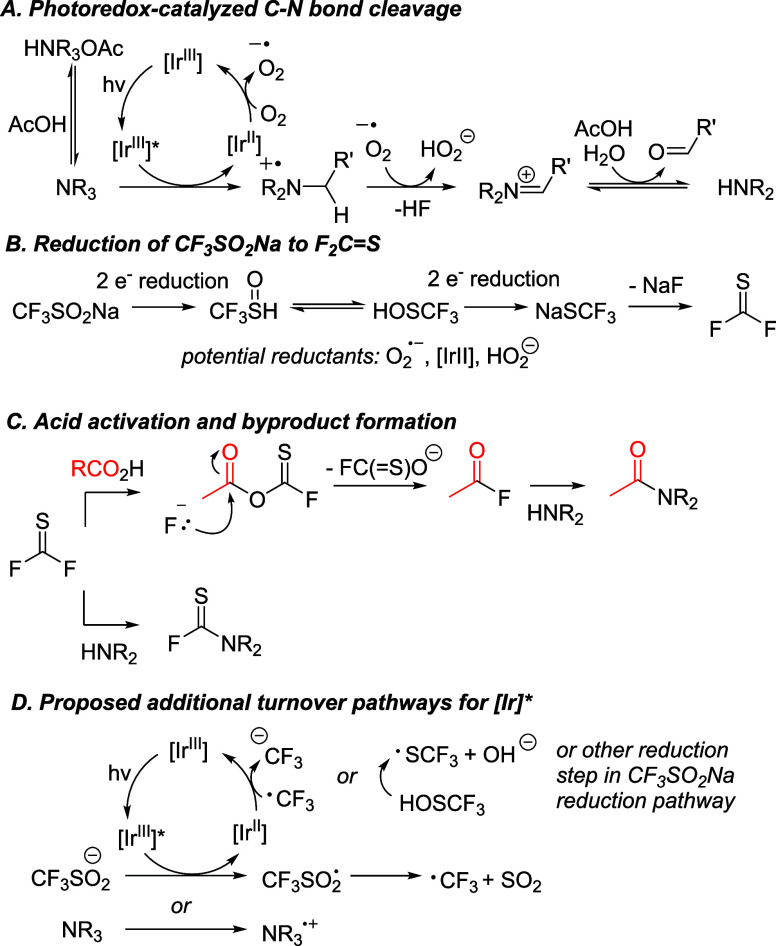
Mechanistic Hypothesis

**Scheme 8:**
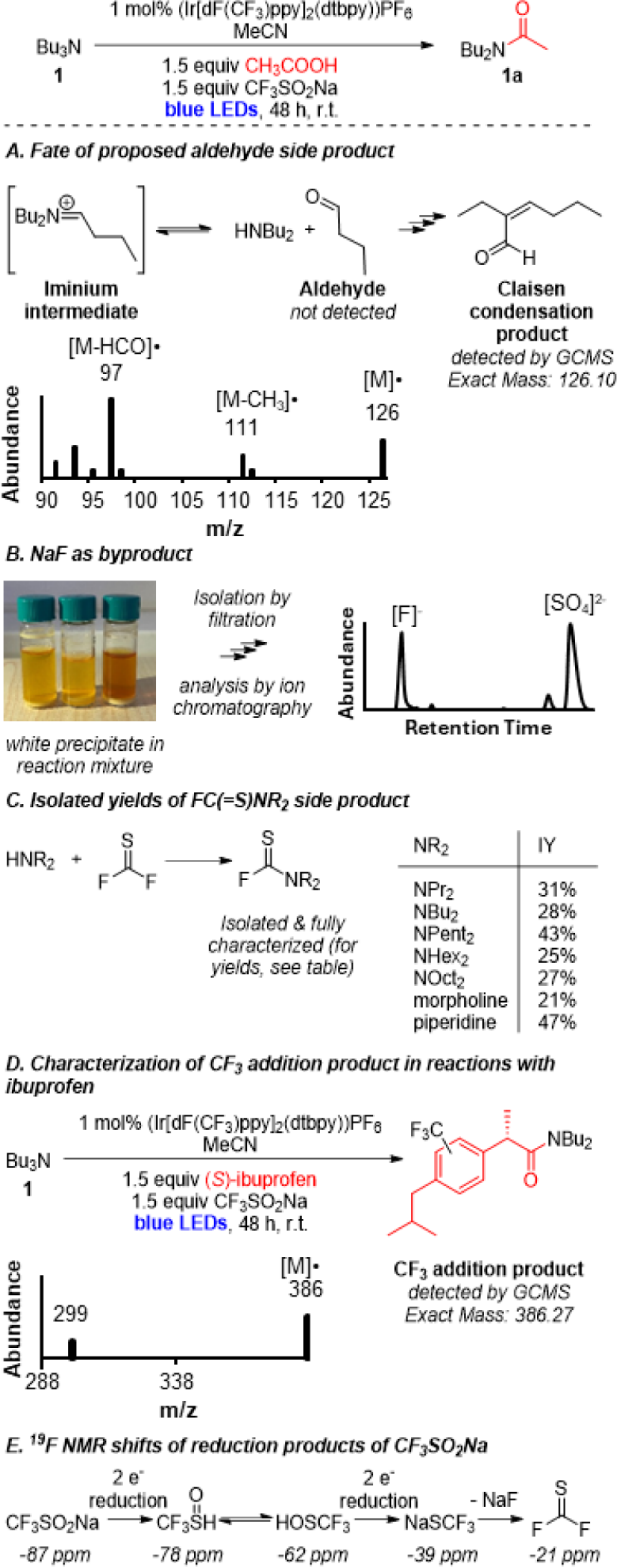
Experimental Support for Mechanistic Proposal

Another key mechanistic proposal (Scheme 7B) is the formation of F_2_C=S as acid-activating intermediate through a reductive pathway from CF_3_SO_2_Na.^31^ Potential reductants to achieve this tranformation include the reduced [Ir^II^] photocatalyst or the O_2_^•–^ radical formed in the photoredox cycle. Alternatively, H_2_O_2_ (from HO_2_) has previously also been proposed to act as a reductant.^32^ The pathway proposed in Scheme 7B suggests that the formation of one equivalent of NaF should occur to successfully access F_2_C=S. NaF would be expected to be insoluble at high concentrations in the reaction medium MeCN. Indeed, a white precipitate is observed in each reaction after the reaction time is complete (see Scheme 8B for a picture). After isolation, this white precipitate, which dissolves readily in water, was analyzed by ion chromatography, confirming NaF as one major component of this precipitate. In addition, the ion chromatogram shows the presence of SO4^2–^, likely formed through air oxidation of SO_2_ and/or SO_3_.^33–36^ This implies (i) that NaSCF_3_ (formed via reduction of CF_3_SO_2_Na) is a reasonable putative precursor for F_2_C=S in the reaction mixture, which can be formed by NaF elimination.

Once F_2_C=S is formed, it can either react with the acid substrate or the secondary amine (Scheme 7C). The latter pathway would lead to the common FC­(=S)­NR_2_ side product, which has been isolated and fully characterized for several amine/acid combinations (e.g., see Scheme 8C). The desired pathway, acid activation by F_2_C=S, would alternatively lead to the formation of an acyl fluoride intermediate, which in turn can react with HNR_2_ to lead to the desired amide product. This hypothesis is consistent with the dependence of the reactivity path on the nature of the acid, as more R_2_NC­(=S)F byproducts should be formed when acid activation is slow. Acid activation by F_2_C=S has previously been postulated,^30,31^ suggesting that it could be a feasible pathway for this reaction as well. The proposed competition between FC­(=S)­NR_2_ and amide formation is key to explaining why an increase in acid concentration (see Figure 1 above) leads to higher yields of the amide product: Higher acid concentrations are expected to favor the desired acid activation/acyl fluoride formation pathway, while concurrently starving the reaction mixture of F_2_C=S available for side product formation.

In addition to the mechanistic proposal outlined in Scheme 7A through 7C, several experimental observations suggest other pathways for [Ir] in the reaction mixture. Those include (Scheme 7D) (i) the direct generation of CF_3_• radicals by [Ir^III^]*, similar to what has been proposed in previous studies;^37–42^ and the (ii) reduction of a fluorinated species by [Ir^II^] (CF_3_• radical or F_3_CSOH or other species from Scheme 7B).

Experimental support for these pathways includes the observation of CF_3_-modified product in reactions with ibuprofen as acid (see Scheme 8D). Traces the CF_3_-modified amide are detected by GCMS, implying that CF_3_• radical formation occurs in small amounts in the reaction mixture. Furthermore, product formation (56%) is observed under N_2_ (see Table 2 above, entry 10) in the absence of oxygen. This suggests that the oxidation of the amine and the regeneration of the [Ir^III^] catalyst can occur without the involvement of O_2_ postulated in Scheme 7A, even though the reaction is more sluggish. We propose that reduced [Ir^II^] catalyst can be regenerated in the absence of abundant O_2_, for through the reduction of CF_3_SO_2_Na or related species, as shown in Scheme 7D.

To gain further insight into the presence of fluorinated, reduced species (CF_3_SO_2_Na and related compounds) in the reaction mixture, we postulated that ^19^F NMR would be a useful tool. Thankfully, the ^19^F NMR shifts of the proposed intermediates (see Scheme 8E) are documented in the literature.^43^ To this end, we designed a suite of control reactions, systematically the proposed acid activator CF_3_SO_2_Na to varying reaction conditions (Table 3).

**Table 3:**
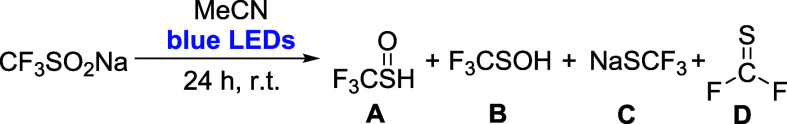
Summary of Control Reactions with CF_3_SO_2_Na to Elucidate Reduction Pathway in Reaction Mixtures Relevant to C–N Bond Cleavage Conditions

Entry	Conditions^a^	Species detected by ^19^F NMR
1	-	-
2	1 equiv CH_3_COOH	-
3	0.66 mol % [Ir]^b^	**A** (28%), **B** (72%)^c^
4	1 equiv CH_3_COOH, 0.66 mol % [Ir]^b^	**A** (31%), **B** (63%), C (6%)
5	1 equiv CH_3_COOH, 0.66 mol % [Ir]^b^, 0.66 equiv NBu_3_	**A** (33%), **B** (40%), **C** (5%), **D** (22%)

a Standard conditions: 0.41 mmol CF_3_SO_2_Na (1 equiv), 3 mL MeCN, air atmosphere, RT, 48 h, blue LEDs.

b [Ir] = (Ir­[dF­(CF_3_)­ppy]_2_(dtbpy))­PF_6_.

c Measured integral ratios were divided by the number of fluorine atoms per compound; the percentages reported here are therefore representative of the ratios of compounds. Percentages were calculated relative to each other, neglecting other peaks.

Interestingly irradiating CF_3_SO_2_Na in the presence or absence of acid substrate did not result in the formation of any reduction products (Table 3, entries 1/2). Adding the Ir photocatalyst to the mixture (entry 3) resulted in a mixture of F_3_CS­(=O)­H (**A**) and F_3_CSOH (**B**), which are tautomeric products from 2-electron reduction of CF_3_SO_2_Na. Adding both Ir catalyst and propionic acid resulted in the formation of **A**, **B**, and the 4-electron reduction product NaSCF_3_ (**C**). Importantly, adding NBu_3_ in the presence of Ir catalyst and propionic acid (entry 5; conditions most relevant to reaction conditions) allowed the detection of **A**, **B**, **C**, and F_2_C=S (**D**) in the mixture. Overall, the direct detection of the reduced species **A**, **B**, **C**, and **D** under catalytically relevant conditions supports the hypothesis that these intermediates play a role in the reaction mechanism, as detailed in Scheme 7 above.

Having established that ^19^F NMR can be a useful tool for mechanistic studies, we postulated that we should be able to detect the relevant fluorinated reduction products in a reaction mixture as well. To this end, we prepared a reaction between NBu_3_ and AcOH under optimized conditions and analyzed the crude mixture after 48 h; the relevant parts of the resulting ^19^F NMR analysis are shown in Figure 2 (for the complete spectrum, see the Supporting Information). The spectrum shows peaks for the side product **1c**, as well as peaks for all other major proposed fluorinated intermediate species (F_2_C=S, NaSCF_3_, F_3_CSOH, F_3_CS­(=O)­H, and NaF), supporting their proposed presence in the reaction mixture.

**Figure 2:**
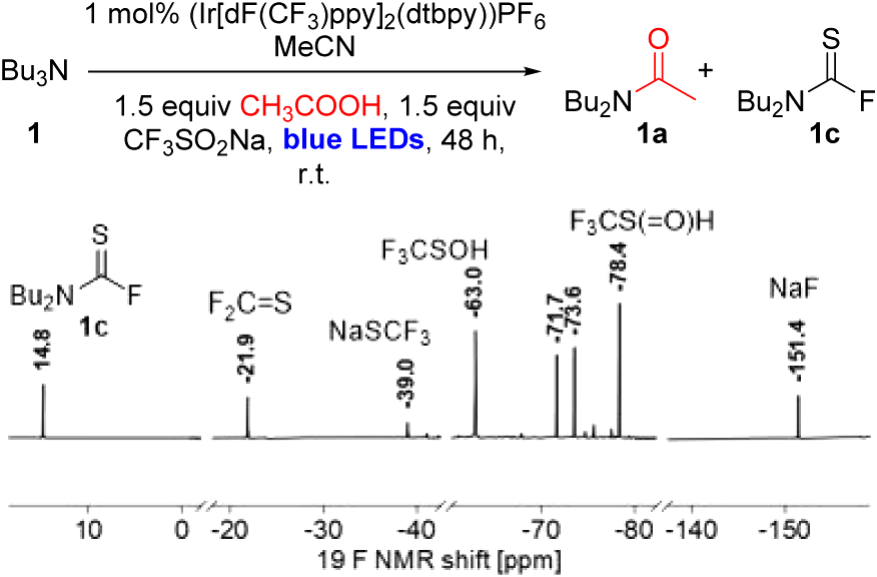
^19^F NMR spectrum of representative reaction mixture after 48 h. Conditions: NBu_3_ (0.27 mmol, 64 μL, 1.0 equiv), 3 mL MeCN, (Ir­[dF­(CF_3_)­ppy]_2_(dtbpy))­PF_6_ (0.0027 mmol, 0.003 g, 0.01 equiv), CF_3_SO_2_Na (0.41 mmol, 0.063 g, 1.5 equiv), CH_3_COOH (0.41 mmol, 24 μL, 1.5 equiv), r.t., 48 h, blue LEDs.

In our final mechanistic investigation, we were curious if the reaction can proceed with a secondary amine substrate instead of a tertiary amine substrate. Based on our mechanistic hypothesis, this reactivity should be possible; however, one would expect formation of [Ir^II^] to proceed less readily, which should lead to less efficient reductive pathways, influencing the efficiency of F_2_C=S formation and, thus, acid activation.

First, we tested if any desired amide product could be obtained when the reaction was executed under the best conditions, but simply with HN^*n*^Bu_2_ instead of N^*n*^Bu_3_ (Table 4, entry 1); this afforded 52% amide product 1a and 17% FC­(=S)­NBu_2_ (**1c**) as side product as well as 26% HNBu_2_ as remaining starting material. As expected, no amide product is formed in the absence of acid substrate (Table 4, entry 2), but 47% **1c** is still obtained, suggesting that the reduction of CF_3_SO_2_Na still proceeds efficiently under these conditions. When the reaction was carried out with HNBu_2_ in the absence of CF_3_SO_2_Na, no amide product or **1c** was obtained (entry 3), which is consistent with CF_3_SO_2_Na acting as acid activator. Finally, no reaction is observed in the absence of Ir catalyst (entries 4, 5, and 6).

**Table 4:**
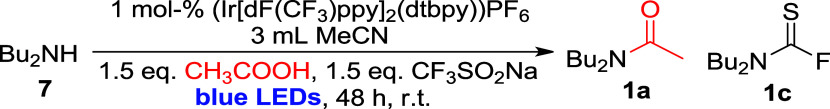
Summary of Control Reactions with HNBu_2_ ^a^

Entry	Changes to conditions	Yield^b^
1	-	52% **1a,** 17% **1c**
2	No CH_3_COOH	47% **1c**
3	No CF_3_SO_2_Na	N.R.^c^
4	No CH_3_COOH, no [Ir]	N.R.^c^
5	No CH_3_COOH, no LEDs, no [Ir]	N.R.^c^
6	No [Ir]	N.R.^c^

a Conditions: HNBu_2_ (0.27 mmol, 45 μL, 1.0 equiv), 3 mL MeCN, (Ir­[dF­(CF_3_)­ppy]_2_(dtbpy))­PF_6_ (0.0027 mmol, 0.003 g, 0.01 equiv), CF_3_SO_2_Na (0.41 mmol, 0.063 g, 1.5 equiv), CH_3_COOH (0.41 mmol, 24 μL, 1.5 equiv), r.t., 48 h, blue LEDs.

b Yields were determined by quantitative, crude by GC using decane as internal standard.

c N.R.: no reaction.

Comparing these results with the yields of amide obtained with NBu_3_ under standard conditions (94% yield; see Table 2 above, entry 1) suggests that cleavage of the C–N bond favors amide product formation. This could be due to two reasons: (i) Limiting the amount of HNBu_2_ in the reaction mixture (i.e., when HNBu_2_ is only present as an intermediate in low concentrations) is expected to slow down the formation of the undesired side product FC­(=S)­NBu_2_ and thus nonproductive consumption of F_2_C=S. (ii) Additionally, the reduction equivalents liberated by oxidative C–N bond cleavage may be able to accelerate the formation of F_2_C=S, in turn accelerating acid activation.

In summary, the above summarized data support three key mechanistic proposals: (i) The formation of secondary amine intermediates through an oxidative, photoredox-catalyzed pathway; (ii) the formation of F_2_C=S as acid activating reagent through a series of reductive steps; (iii) other potential one electron oxidation and reduction pathways that are available under modified conditions, including [Ir] turnover in the absence of O_2_ and potential CF_3_• radical formation.

## Summary and Conclusions

Overall, this manuscript describes the establishment of versatile conditions for the synthesis of amides via C–N bond cleavage of tertiary amines. Both the tertiary amine scope and the acid scope significantly advance the state of the art: The conditions are shown to be operational with previously unreactive amine substrates (tri-*n*-butyl amine, *N*-alkyl morpholine) and are operational with a wide variety of complex acid reactants. Interestingly, our studies suggest that the additive CF_3_SO_2_Na is crucially involved in carboxylic acid activation through an acyl fluoride intermediate. Overall, this work expands available options for carboxylic acid activation and provides new insights into amide formations via C–N bond cleavage mechanisms.

## Experimental Section

### General Information

All chemicals were purchased from commercial suppliers (e.g., Alfa Aesar, TCI America, Sigma-Aldrich) and used as received unless otherwise stated in the procedure. Deuterated solvents used for NMR measurements were purchased from Cambridge Isotopes or Sigma-Aldrich.

The light source is from the company Solid Apollo. The model “Blue 5050 72W” is a blue colored LED strip with a wavelength of 465 nm. This strip was glued to a glass container and the vials in which the reactions were carried out were placed in this container (see Supporting Information for details). The 4 mL vials used in the reactions were purchased from Chemglass (CG-4904-06).

All NMR experiments were carried out on Bruker BioSpin 400 MHz Avance III Digital NMR spectrometers. All quantitative ^1^H NMR measurements were performed using an adjusted method (15 s relaxation time, NS = 32) with 1,3-dinitrobenzene or *p*-xylene as internal standards. All NMR spectra were recorded at room temperature unless otherwise noted. The signal for nondeuterated solvent (δ 7.26 ppm for CDCl_3_) was used as an internal reference for ^1^H NMR spectra. For ^13^C­{H} NMR spectra, chemical shifts are reported relative to the solvent resonance of CDCl_3_ at δ 77.0 ppm.

High-resolution mass spectrometry (HR-MS) was carried out by the mass spectrometry service of the Central Laboratory at Middle East Technical University, Turkey. Masses are reported in *m*/*z* units as the molecule ion as [M + H]^+^ or [M + Na]^+^.

### General Procedure

A tertiary amine (0.27 mmol, 1.0 equiv) was dissolved in 3 mL MeCN and then RCOOH (0.41 mmol, 1.5 equiv), CF_3_SO_2_Na (0.41 mmol, 0.063 g, 1.5 equiv) and [Ir­(dF­(CF_3_)­ppy)_2_(dtbbpy)]­PF_6_ (0.0027 mmol, 0.003 g, 1.0 mol %) was added to the stirred solution under an air atmosphere. The vial was then sealed tightly with a Teflon-lined vial cap and the mixture was stirred at room temperature under irradiation with blue LEDs for 48 h. Reactions were monitored by TLC. After the reaction time was completed, the solvent was removed under reduced pressure.

To determine crude assay yields by GC, decane or dodecane was added to the reaction mixture. The mixture was sampled by diluting an aliquot with MeOH, followed by filtration, and analysis of the filtrate by GC-FID.

To determine crude assay yields by quantitative ^1^H NMR, the reaction mixture was evaporated. Then, CDCl_3_ and 1,1,2-trichloroethane (3.47 μL, 37.4 μmol; 0.14 equiv) or *p*-xylene (11 μL, 0.27 mmol; 1.0 equiv) as internal standard were added. The resulting suspension was mixed well, and analyzed by quantitative ^1^H NMR.

### Characterization and Spectral Data

#### *N,N*-Dibutylacetamide (**1a**)

Purification solvent: 1:1 EtOAc/Hexane, light yellow oil, yield 40.7 mg (88%).^1^H NMR (400 MHz, CDCl_3_): δ 3.33–3.27 (m, 2H), 3.24–3.17 (m, 2H), 2.07 (s, 3H), 1.59–1.45 (m, 4H), 1.39–1.24 (m, 4H), 0.95 (t, *J* = 6.4 Hz, 3H), 0.92 (t, *J* = 6.4 Hz, 3H) ppm. The spectral data were in agreement with literature data.^18,44^

### Gram Scale Reaction with Tri-*N*-butylamine (**1**)

NBu_3_ (1 mmol, 237 μL, 1.0 equiv), 12 mL MeCN, Ir­[dF­(CF_3_)­ppy]_2_(dtbpy))­PF_6_ (0.01 mmol, 0.011 g, 0.01 equiv), CH_3_COOH (1.52 mmol, 89 μL, 1.5 equiv) and CF_3_SO_2_Na (1.52 mmol, 0.23 g, 1.5 equiv) were mixed into a 20 mL pressure vessel glass (Chemglass) with a stir bar and internal thread cap. The reaction mixture was stirred for 48 h at room temperature while irradiating with blue LEDs. Yield: 84%.

#### *N,N*-Dipropylacetamide (**3a**)

Purification solvent: 1:1 EtOAc/Hexane, colorless oil, yield 32.3 mg (85%). ^1^H NMR (400 MHz, CDCl_3_): δ 3.32–3.24 (m, 2H), 3.23–3.15 (m, 2H), 2.08 (s, 3H), 1.66–1.50 (m, 4H), 0.96–0.85 (m, 6H) ppm. The spectral data were in agreement with literature data.^45^

#### *N,N*-Dipentylacetamide (**4a**)

Purification solvent: 1:1 EtOAc/Hexane, colorless oil, yield 45.8 mg (86%). ^1^H NMR (400 MHz, CDCl_3_) δ 3.32–3.26 (m, 2H), 3.24–3.16 (m, 2H), 2.07 (s, 3H), 1.54 (tt, *J* = 15.4, 7.6 Hz, 4H), 1.39–1.21 (m, 8H), 0.96–0.86 (m, 6H) ppm. The spectral data were in agreement with literature data.^46^

#### *N,N*-Dihexylacetamide (**5a**)

Purification solvent: 1:3 EtOAc/Hexane, colorless oil, yield 52.1 mg (85%). ^1^H NMR (400 MHz, CDCl_3_) δ 3.32–3.25 (m, 2H), 3.24–3.17 (m, 2H), 2.07 (s, 3H), 1.59–1.46 (m, 4H), 1.37–1.22 (m, 12H), 0.94–0.85 (m, 6H) ppm. The spectral data were in agreement with literature data.^47^

#### *N,N*-Dioctylacetamide (**6a**)

Purification solvent: 1:4 EtOAc/Hexane, light yellow oil, yield 66.1 mg (87%). ^1^H NMR (400 MHz, CDCl_3_) δ 3.32–3.26 (m, 2H), 3.23–3.17 (m, 2H), 2.07 (s, 3H), 1.60–1.44 (m, 4H), 1.32–1.23 (m, 20H), 0.93–0.85 (m, 6H) ppm. The spectral data were in agreement with literature data.^48^

#### 1-Morpholinoethanone (**7a**)

Purification solvent: 1:3 EtOAc/Hexane, light yellow oil, yield 22.1 mg (64%). ^1^H NMR (400 MHz, CDCl_3_) δ 4.01–3.97 (m, 2H), 3.84–3.79 (m, 2H), 3.78–3.69 (m, 4H), 1.56 (s, 3H) ppm. The spectral data were in agreement with literature data.^44^

#### 1-(Piperidin-1-yl)­ethanone (**8a**)

Purification solvent: 1:5 EtOAc/Hexane, light yellow oil, yield 22.8 mg (67%). ^1^H NMR (400 MHz, CDCl_3_) δ 3.58–3.51 (m, 2H), 3.42–3.36 (m, 2H), 2.08 (s, 3H), 1.66–1.60 (m, 2H), 1.56–1.48 (m, 4H) ppm. The spectral data were in agreement with literature data.^44,48^

#### *N,N*-Dibutylpropionamide (**1b**)

Purification solvent: EtOAc, yellow oil, yield 30.2 mg (61%). ^1^H NMR (400 MHz, CDCl_3_): ^1^H NMR (400 MHz, CDCl_3_) δ 3.30 (t, *J* = 7.7 Hz, 2H), 3.22 (t, *J* = 7.6 Hz, 2H), 2.32 (dd, *J* = 14.8, 7.4 Hz, 2H), 1.52 (tt, *J* = 15.2, 7.6 Hz, 4H), 1.32 (tt, *J* = 15.2, 7.6 Hz, 4H), 1.14 (t, *J* = 7.4 Hz, 3H), 0.94 (dt, *J* = 11.3, 7.3 Hz, 6H) ppm. ^13^C­{H} NMR (101 MHz, CDCl_3_) δ 173.24, 47.61, 45.65, 31.18, 29.94, 26.26, 20.27, 20.12, 13.87, 9.62 ppm. The spectral data were in agreement with literature data.^49^

#### *N,N-*Dipropylpropionamide (**3b**)

Purification solvent: 1:3 EtOAc/Hexane, colorless oil, yield 23.8 mg (56%). ^1^H NMR (400 MHz, CDCl_3_) δ 3.30–3.25 (m, 2H), 3.21–3.15 (m, 2H), 2.33 (q, *J* = 7.4 Hz, 2H), 1.64–1.50 (m, 4H), 1.14 (t, *J* = 7.5 Hz, 3H), 0.91 (t, *J* = 6.6 Hz, 3H), 0.88 (t, *J* = 6.6 Hz, 3H) ppm. ^13^C­{H} NMR (101 MHz, CDCl_3_) δ 210.85, 49.56, 47.55, 26.31, 22.25, 20.99, 11.24, 9.69 ppm. The spectral data were in agreement with literature data.^49^

#### *N,N-*Dipentylpropionamide (**4b**)

Purification solvent: 1:3 EtOAc/Hexane, light yellow oil, yield 28.2 mg (49%). ^1^H NMR (400 MHz, CDCl_3_) δ 3.34–3.26 (m, 2H), 3.24–3.16 (m, 2H), 2.31 (q, *J* = 7.4 Hz, 2H), 1.56–1.47 (m, 4H), 1.39–1.28 (m, 8H), 1.14 (t, *J* = 7.4 Hz, 3H), 0.94–0.85 (m, 6H) ppm. ^13^C­{H} NMR (101 MHz, CDCl_3_) δ 173.24, 53.39, 47.86, 45.90, 29.32, 29.25, 29.09, 28.79, 27.50, 26.30, 22.44, 14.01, 13.96, 9.66 ppm. HRMS (ESI), *m*/*z*: calculated for C_13_H_27_NO [M + H]^+^: 213.2092, found: 213.2091.

#### *N,N-*Dihexylpropionamide (**5b**)

Purification solvent: 1:3 EtOAc/Hexane, viscous oil, yield 44.3 mg (68%). ^1^H NMR (400 MHz, CDCl_3_): ^1^H NMR (400 MHz, CDCl_3_) δ 3.32–3.25 (m, 2H), 3.23–3.16 (m, 2H), 2.31 (q, *J* = 7.4 Hz, 2H), 1.56–1.46 (m, 4H), 1.36–1.24 (m, 12H), 1.14 (t, *J* = 7.4 Hz, 3H), 0.93–0.84 (m, 6H) ppm. ^13^C­{H} NMR (101 MHz, CDCl_3_) δ 173.23, 47.90, 45.94, 31.64, 31.55, 29.09, 27.79, 26.74, 26.61, 26.30, 22.59, 14.01, 13.96, 9.67 ppm. The spectral data were in agreement with literature data.^49^

#### *N,N-*Dioctylpropionamide (**6b**)

Purification solvent: 1:3 EtOAc/Hexane, viscous oil, yield 47.4 mg (59%).^1^H NMR (400 MHz, CDCl_3_) δ 3.32–3.26 (m, 2H), 3.23–3.16 (m, 2H), 2.31 (q, *J* = 7.3 Hz, 2H), 1.56–1.45 (m, 4H), 1.27 (bs, 20H), 1.14 (t, *J* = 7.4 Hz, 3H), 0.92–0.83 (m, 6H) ppm. The spectral data were in agreement with literature data.^50^

#### 1-(Piperidin-1-yl)­propan-1-one (**8b**)

Purification solvent: 1:1 EtOAc/Hexane, light yellow oil, yield 24.8 mg (65%).^1^H NMR (400 MHz, CDCl_3_): ^1^H NMR (400 MHz, CDCl_3_) δ 3.59–3.52 (m, 2H), 3.43–3.36 (m, 2H), 2.34 (q, *J* = 7.5 Hz, 2H), 1.68–1.51 (m, 6H), 1.14 (t, *J* = 7.5 Hz, 3H) ppm. ^13^C­{H} NMR (101 MHz, CDCl_3_) δ 172.14, 46.53, 42.67, 29.32, 26.56, 25.57, 24.62, 9.63 ppm. The spectral data were in agreement with literature data.^51^

#### Dibutylcarbamothioic Fluoride (**1c**)

Purification solvent: 1:1 EtOAc/Hexane.^1^H NMR (400 MHz, CDCl_3_): ^1^H NMR (400 MHz, CDCl_3_) δ 3.69–3.62 (m, 2H), 3.41 (td, *J* = 7.5, 1.8 Hz, H), 1.76 – 1.67 (m, 2H), 1.61 (dt, *J* = 12.8, 7.6 Hz, 2H), 1.36 (sext, *J* = 7.4 Hz, 4H), 0.99–0.92 (m, 6H) ppm. The spectral data were in agreement with literature data.^52^

#### Diethylcarbamothioic Fluoride (**2c**)

Purification solvent: 1:3 EtOAc/Hexane. ^1^H NMR (400 MHz, CDCl_3_) δ 3.75 (q, *J* = 7.2 Hz, 2H), 3.50 (qd, *J* = 7.2, 1.9 Hz, 2H), 1.31 (td, *J* = 7.2, 1.0 Hz, 3H), 1.26 (t, *J* = 7.2 Hz, 3H) ppm. The spectral data were in agreement with literature data.^53^

#### Dipropylcarbamothioic Fluoride (**3c**)

Purification solvent: 1:4 EtOAc/Hexane. ^1^H NMR (400 MHz, CDCl_3_) δ 3.71–3.54 (m, 2H), 3.40 (t, *J* = 7.5 Hz, 2H), 1.77 (dq, *J* = 15.0, 7.4 Hz, 2H), 1.67 (td, *J* = 14.8, 7.4 Hz, 2H), 1.02–0.88 (m, 6H) ppm. ^13^C­{H} NMR (101 MHz, CDCl_3_) δ 183.17, 179.98, 55.49 (d, *J* = 5.9 Hz), 51.61 (d, *J* = 5.0 Hz), 21.56 (s), 19.32 (s), 11.16 (s), 11.08 (s) ppm. ^19^F NMR (377 MHz, CDCl_3_) δ 15.17 ppm.

#### Dipentylcarbamothioic Fluoride (**4c**)

Purification solvent: 1:9 EtOAc/Hexane. ^1^H NMR (400 MHz, CDCl3) δ 3.68–3.61 (m, 2H), 3.44–3.38 (m, 2H), 1.73 (dt, *J* = 15.0, 7.6 Hz, 2H), 1.63 (dt, *J* = 15.0, 7.6 Hz, 2H), 1.41–1.26 (m, 8H), 0.95–0.86 (m, 6H) ppm. ^13^C­{H} NMR (101 MHz, CDCl_3_) δ 179.81, 175.69, 53.93 (d, *J* = 5.9 Hz), 50.01 (d, *J* = 5.0 Hz), 28.87 (s), 28.77 (s), 27.91 (s), 25.58 (s), 22.31 (s), 22.23 (s), 13.90 (s), 13.83 (s) ppm. ^19^F NMR (377 MHz, CDCl_3_) δ 14.94 ppm.

#### Dihexylcarbamothioic Fluoride (**5c**)

Purification solvent: 1:9 EtOAc/Hexane. ^1^H NMR (400 MHz, CDCl_3_) δ 3.68–3.61 (m, 2H), 3.41 (t, *J* = 7.0 Hz, 2H), 1.76–1.67 (m, 2H), 1.66–1.59 (m, 2H), 1.37–1.25 (m, 12H), 0.93–0.86 (m, 6H) ppm. The spectral data were in agreement with literature data.^52^

#### Dioctylcarbamothioic Fluoride (**6c**)

Purification solvent: 1:9 EtOAc/Hexane.^1^H NMR (400 MHz, CDCl_3_) δ 3.68–3.60 (m, 2H), 3.45–3.36 (m, 2H), 1.76–1.66 (m, 2H), 1.66–1.59 (m, 2H), 1.37–1.20 (m, 20H), 0.88 (t, *J* = 6.8 Hz, 6H) ppm. The spectral data were in agreement with literature data.^54^

#### Morpholine-4-carbothioyl Fluoride (**7c**)

Purification solvent: 1:1 EtOAc/Hexane.^1^H NMR (400 MHz, CDCl_3_) δ 4.01–3.97 (m, 2H), 3.83–3.80 (m, 2H), 3.77–3.70 (m, 4H) ppm. The spectral data were in agreement with literature data.^52^

#### Piperidine-1-carbothioyl Fluoride (8c)

Purification solvent: 1:3 EtOAc/Hexane.^1^H NMR (400 MHz, CDCl_3_) δ 3.96–3.90 (m, 2H), 3.71–3.64 (m, 2H), 1.78–1.61 (m, 6H) ppm. The spectral data were in agreement with literature data.^52^

#### (*S*)-*N,N-*Dibutyl-2-(4-isobutylphenyl)­propanamide (**1d**)

Purification solvent: 1:1 EtOAc/Hexane, yield 57.3 mg (67%). ^1^H NMR (400 MHz, CDCl_3_) δ 7.16 (d, *J* = 8.1 Hz, 2H), 7.07 (d, *J* = 8.1 Hz, 2H), 3.79 (q, *J* = 6.9 Hz, 1H), 3.43 (ddd, *J* = 15.0, 10.4, 5.5 Hz, 1H), 3.27–3.12 (m, 2H), 3.08–2.97 (m, 1H), 2.43 (d, *J* = 7.2 Hz, 2H), 1.83 (sept, *J* = 6.7 Hz, 1H), 1.49–1.38 (m, 7H), 1.31–1.21 (m, 4H), 0.94–0.85 (m, 12H) ppm. ^13^C­{H} NMR (101 MHz, CDCl_3_) δ 173.36, 140.00, 139.73, 129.43, 126.98, 53.39, 47.43, 45.86, 45.04, 42.83, 31.14, 30.16, 29.69, 22.35, 20.97, 20.24, 20.09, 13.83, 13.76 ppm. HRMS (ESI), *m*/*z*: calculated for C_21_H_35_NO [M + H]^+^: 318.2797, found: 318.2797.

#### (*S*)-*N,N*-Diethyl-2-(4-isobutylphenyl)­propanamide (**2d**)

Purification solvent: 1:3 EtOAc/Hexane, yield 39.5 mg (56%). ^1^H NMR (400 MHz, CDCl_3_) δ 7.22 (d, *J* = 8.1 Hz, 2H), 7.10 (d, *J* = 8.0 Hz, 2H), 3.71 (q, *J* = 7.2 Hz, 1H), 3.53–3.41 (m, 1H), 3.35–3.21 (m, 1H), 3.13 (dd, *J* = 14.8, 7.2 Hz, 1H), 2.44 (d, *J* = 7.2 Hz, 2H), 1.91–1.79 (m, 1H), 1.50 (d, *J* = 7.2 Hz, 3H), 0.93–0.83 (m, 12H) ppm. The spectral data were in agreement with literature data.^55^

#### (*S*)-2-(4-Isobutylphenyl)-*N,N*-dipentylpropanamide (**3d**)

Purification solvent: 1:4 EtOAc/Hexane, yield 52.3 mg (67%). ^1^H NMR (400 MHz, CDCl_3_) δ 7.16 (d, *J* = 8.1 Hz, 2H), 7.07 (d, *J* = 8.1 Hz, 2H), 3.81 (q, *J* = 6.8 Hz, 1H), 3.41 (ddd, *J* = 13.4, 9.3, 6.2 Hz, 1H), 3.26–3.08 (m, 2H), 3.04–2.95 (m, 1H), 2.43 (d, *J* = 7.2 Hz, 2H), 1.83 (sept, *J* = 6.8 Hz, 1H), 1.56–1.45 (m, 4H), 1.42 (d, *J* = 6.9 Hz, 3H), 0.94–0.76 (m, 12H) ppm. ^13^C­{H} NMR (101 MHz, CDCl_3_) δ 173.51, 140.01, 139.72, 129.43, 127.01, 49.29, 47.72, 45.04, 42.83, 30.16, 22.33, 22.19, 20.92, 20.80, 11.35, 11.15 ppm. HRMS (ESI), *m*/*z*: calculated for C_19_H_31_NO [M + H]^+^: 290.2484, found: 290.2469.

#### (*S*)-2-(4-Isobutylphenyl)-*N,N*-dipentylpropanamide (**4d**)

Purification solvent: 1:9 EtOAc/Hexane, yield 48.5 mg (52%). ^1^H NMR (400 MHz, CDCl_3_) δ 7.16 (d, *J* = 8.1 Hz, 2H), 7.07 (d, *J* = 8.0 Hz, 2H), 3.79 (q, *J* = 6.8 Hz, 1H), 3.48 – 3.39 (m, 1H), 3.30 – 3.18 (m, 1H), 3.14 (ddd, *J* = 13.3, 8.7, 6.4 Hz, 1H), 3.00 (ddd, *J* = 15.0, 10.3, 5.0 Hz, 1H), 2.43 (d, *J* = 7.2 Hz, 2H), 1.83 (sept, *J* = 6.6 Hz, 1H), 1.55–1.45 (m, 4H), 1.41 (d, *J* = 6.9 Hz, 3H), 1.35–1.19 (m, 8H), 0.96–0.82 (m, 12H) ppm. ^13^C­{H} NMR (101 MHz, CDCl_3_) δ 173.36, 139.99, 139.73, 129.42, 126.99, 53.38, 47.61, 46.00, 45.05, 42.81, 30.15, 29.20, 29.06, 28.74, 27.24, 22.45, 22.37, 20.96, 14.01, 13.93 ppm. HRMS (ESI), *m*/*z*: calculated for C_23_H_39_NO [M + H]^+^: 346.3110, found: 346.3116.

#### (*S*)-*N,N*-Dihexyl-2-(4-isobutylphenyl)­propanamide (**5d**)

Purification solvent: 1:9 EtOAc/Hexane, yield 68.1 mg (70%). ^1^H NMR (400 MHz, CDCl_3_) δ 7.16 (d, *J* = 8.1 Hz, 2H), 7.06 (d, *J* = 8.1 Hz, 2H), 3.79 (q, *J* = 6.8 Hz, 1H), 3.49–3.38 (m, 1H), 3.30–3.19 (m, 1H), 3.14 (ddd, *J* = 13.3, 8.9, 6.2 Hz, 1H), 3.00 (ddd, *J* = 14.9, 10.3, 4.7 Hz, 1H), 2.43 (d, *J* = 7.2 Hz, 2H), 1.84 (sept, *J* = 6.7 Hz, 1H), 1.55–1.45 (m, 4H), 1.41 (d, *J* = 6.8 Hz, 3H), 1.31–1.16 (m, 12H), 0.94–0.83 (m, 12H) ppm. ^13^C­{H} NMR (101 MHz, CDCl_3_) δ 173.36, 139.99, 139.73, 129.43, 126.99, 47.65, 46.05, 45.05, 42.82, 31.61, 31.52, 30.15, 29.01, 27.54, 26.69, 26.58, 22.60, 22.55, 22.36, 20.97, 13.95 ppm. HRMS (ESI), *m*/*z*: calculated for C_25_H_43_NO [M + H]^+^: 374.3423, found: 324.3422.

#### (*S*)-2-(4-Isobutylphenyl)-*N,N*-dioctylpropanamide (**6d**)

Purification solvent: 1:9 EtOAc/Hexane, yield 77.5 mg (69%). ^1^H NMR (400 MHz, CDCl_3_) δ 7.15 (d, *J* = 8.1 Hz, 2H), 7.06 (d, *J* = 8.1 Hz, 2H), 3.79 (q, *J* = 6.9 Hz, 1H), 3.48–3.39 (m, 1H), 3.28 – 3.18 (m, 1H), 3.18–3.09 (m, 1H), 2.99 (ddd, *J* = 15.0, 10.4, 4.8 Hz, 1H), 2.43 (d, *J* = 7.2 Hz, 2H), 1.84 (sept, *J* = 6.7 Hz, 1H), 1.54–1.44 (m, 4H), 1.41 (d, *J* = 6.8 Hz, 3H), 1.32–1.18 (m, 20H), 0.94–0.83 (m, 12H) ppm. ^13^C­{H} NMR (101 MHz, CDCl_3_) δ 173.36, 139.99, 139.75, 129.43, 126.99, 53.38, 47.64, 46.06, 45.06, 42.81, 31.80, 30.15, 29.29, 27.60, 27.06, 26.92, 22.63, 22.37, 20.97, 14.05 ppm. HRMS (ESI), *m*/*z*: calculated for C_29_H_51_NO [M + H]^+^: 430.4049, found: 430.4057.

#### (*S*)-2-(4-Isobutylphenyl)-1-morpholinopropan-1-one (**7d**)

Purification solvent: 1:1 EtOAc/Hexane, yield 55.1 mg (74%). ^1^H NMR (400 MHz, CDCl_3_) δ 7.22 (d, *J* = 8.1 Hz, 2H), 7.10 (d, *J* = 8.1 Hz, 2H), 4.05–3.95 (m, 2H), 3.84–3.79 (m, 2H), 3.77–3.69 (m, 5H), 2.45 (d, *J* = 7.2 Hz, 2H), 1.84 (sept, *J* = 6.7 Hz, 1H), 1.51 (d, *J* = 7.2 Hz, 3H), 0.89 (d, *J* = 6.6 Hz, 6H) ppm. ^13^C­{H} NMR (101 MHz, CDCl_3_) δ 178.76, 140.85, 137.10, 129.41, 127.26, 65.79, 45.06, 44.73, 30.14, 22.38, 18.19 ppm. HRMS (ESI), *m*/*z*: calculated for C_17_H_25_NO_2_ [M + H]^+^: 276.1964, found: 276.1973.

#### (*S*)-2-(4-Isobutylphenyl)-1-(piperidin-1-yl)­propan-1-one (**8d**)

Purification solvent: 1:3 EtOAc/Hexane, yield 35.4 mg (48%). ^1^H NMR (400 MHz, CDCl_3_) δ 7.22 (d, *J* = 8.1 Hz, 2H), 7.11 (d, *J* = 8.1 Hz, 2H), 4.04–3.99 (m, 1H), 3.75–3.70 (m, 1H), 3.62–3.58 (m, 1H), 2.63 (d, *J* = 7.6 Hz, 1H), 2.45 (d, *J* = 7.2 Hz, 2H), 2.26–2.19 (m, 1H), 1.84 (sept, *J* = 6.7 Hz, 1H), 1.80–1.58 (m, 6H), 1.51 (d, *J* = 7.2 Hz, 3H), 0.90 (d, *J* = 6.6 Hz, 6H) ppm. ^13^C­{H} NMR (101 MHz, CDCl_3_) δ 185.82, 140.85, 137.10, 129.41, 127.26, 53.40, 45.06, 44.74, 30.14, 26.74, 24.83, 22.38, 18.19 ppm. HRMS (ESI), *m*/*z*: calculated for C_18_H_27_NO [M + H]^+^: 274.2171, found: 274.2172.

#### *N,N*-Dibutylbenzamide (**10**)

Purification solvent: 1:9 EtOAc/Hexane, yield 34.6 mg (55%).^1^H NMR (400 MHz, CDCl_3_) δ 7.51–7.35 (m, 5H), 3.64–3.40 (m, 2H), 3.30–3.12 (m, 2H), 1.67 (s, 2H), 1.54–1.41 (m, 4H), 1.16 (d, *J* = 5.5 Hz, 2H), 1.11–0.94 (m, 3H), 0.89–0.71 (m, 3H) ppm. The spectral data were in agreement with literature data.^15^

## Supplementary Material



## Data Availability

The data underlying this study are available in the published article and its Supporting Information.

## Notes

Reaction optimization details, synthetic procedures, characterization for new and known compounds (PDF)
