# Supplementary material for: Photoredox Driven Amide Synthesis from Tertiary Amines and Carboxylic Acids via C–N Bond Cleavage
Source: ACS Omega. 2025 Jul 23;10(30):33755–66. doi: 10.1021/acsomega.5c04718 (PMC12332612; doi:10.1021/acsomega.5c04718)

Supporting Information

Photoredox driven amide synthesis from tertiary amines and carboxylic acids via C–N Bond cleavage

Merve Dogan[‡], Irem Akgul[‡], Marion H. Emmert[*§],Ozgur Yilmaz[*‡]

[‡]Department of Chemistry, Faculty of Sciences, Mersin University, 33343 Mersin, Turkey.

[§]Discovery Chemistry, MRL, Merck & Co., Inc., 770 Sumneytown Pike, West Point, PA 19486, USA.

Email: marion.emmert@merck.com

Email: yilmazozgur@mersin.edu.tr

Table of Contents

Optimization of Conditions 4

General Procedure for Amide synthesis after optimization (Table S5, Entry 2) 4

Selected GCMS spectra of crude reaction mixtures during optimization studies 7

Tabular summary of substrate scope results 10

NMR and GCMS spectra of products 19

_N,N_-dibutylacetamide ($C_{10}H_{21}NO$, 1a) 19

_N,N_-diethylacetamide ($C_6H_{13}NO$, 2a) 20

_N,N_-dipropylacetamide ($C_8H_{17}NO$, 3a) 21

_N,N_-dipentylacetamide ($C_{12}H_{25}NO$, 4a) 22

_N,N_-dihexylacetamide ($C_{14}H_{29}NO$, 5a) 23

_N,N_-dioctylacetamide ($C_{18}H_{37}NO$, 6a) 24

1-morpholinoethanone ($C_6H_{11}NO_2$, 7a) 25

1-(piperidin-1-yl)ethanone ($C_7H_{13}NO$, 8a) 26

_N,N_-dibutylpropionamide ($C_{11}H_{23}NO$, 1b) 27

_N,N_-diethylpropionamide ($C_7H_{15}NO$, 2b) 28

_N,N_-dipropylpropionamide ($C_9H_{19}NO$, 3b) 29

_N,N_-dipentylpropionamide ($C_{13}H_{27}NO$, 4b) 30

_N,N_-dihexylpropionamide ($C_{15}H_{31}NO$, 5b) 31

_N,N_-dioctylpropionamide ($C_{19}H_{39}NO$, 6b) 32

1-(piperidin-1-yl)propan-1-one ($C_8H_{15}NO$, 8b) 33

Dibutylcarbamothioic fluoride ($C_9H_{18}FNS$, 1c) 34

Diethylcarbamothioic fluoride ($C_5H_{10}FNS$, 2c) 35

Dipropylcarbamothioic fluoride ($C_7H_{14}FNS$, 3c) 36

Dipentylcarbamothioic fluoride ($C_{11}H_{22}FNS$, 4c) 38

Dihexylcarbamothioic fluoride ($C_{13}H_{26}FNS$, 5c) 40

Dioctylcarbamothioic fluoride ($C_{17}H_{34}F_3N$, 6c) 41

Morpholine-4-carbothioyl fluoride (C_5H_8FNOS, 7c) 42

Piperidine-1-carbothioyl fluoride ($C_6H_{10}FNS$, 8c) 43

(S)-_N,N_-dibutyl-2-(4-isobutylphenyl)propanamide ($C_{21}H_{35}NO$, 1d) 44

(S)-_N,N_-diethyl-2-(4-isobutylphenyl)propanamide ($C_{17}H_{27}NO$, 2d) 46

(S)-2-(4-isobutylphenyl)-_N,N_-dipropylpropanamide ($C_{19}H_{31}NO$, 3d) 47

(S)-2-(4-isobutylphenyl)-_N,N_-dipentylpropanamide ($C_{23}H_{39}NO$, 4d) 49

(S)-_N,N_-dihexyl-2-(4-isobutylphenyl)propanamide ($C_{25}H_{43}NO$, 5d) 51

(S)-2-(4-isobutylphenyl)-_N,N_-dioctylpropanamide ($C_{29}H_{51}NO$, 6d) 53

(S)-2-(4-isobutylphenyl)-1-morpholinopropan-1-one ($C_{17}H_{25}NO$, 7d) ... 55

(S)-2-(4-isobutylphenyl)-1-(piperidin-1-yl)propan-1-one ($C_{18}H_{27}NO$, 8d) ... 57

Comparison of ^{19}F NMR spectra for control reactions ... 59

Comparison of ^{19}F NMR spectra at different reaction times ... 65

Procedure for reactions of complex acids with N-methyl morpholine ... 69

LCMS data for reactions of complex acids with N-methyl morpholine ... 71

1,3-Dimethyl-7-(2-morpholino-2-oxoethyl)-1H-purine-2,6(3H,7H)-dione (11a) ... 71

Anthracen-9-yl(morpholino)methanone (12a) ... 71

(2-Methyl-2,4,5,6-tetrahydrocyclopenta[c]pyrazol-3-yl)(morpholino)methanone (13a) ... 72

Morpholino(2-phenylimidazo[1,2-a]pyridin-3-yl)methanone (14a) ... 72

(2,5-Dichloro-4,6-dimethylpyridin-3-yl)(morpholino)methanone (15a) ... 73

[1,2,4]Triazolo[1,5-a]pyrimidin-6-yl(morpholino)methanone (16a) ... 73

3-(3,4-Dihydro-1H-carbazol-9(2H)-yl)-1-morpholinopropan-1-one (17a) ... 74

1-(3-Chlorophenyl)-6-morpholinohexane-1,6-dione (18a) ... 74

3-(4-(1H-Imidazol-1-yl)phenyl)-1-morpholinopropan-1-one (19a) ... 75

(5-Chlorothieno[3,2-b]pyridin-3-yl)(morpholino)methanone (20a) ... 75

Morpholino(3-propoxypyridin-2-yl)methanone (21a) ... 76

7-(2-Morpholino-2-oxoethyl)-2H-benzo[b][1,4]oxazin-3(4H)-one (22a) ... 76

(4-(Heptyloxy)phenyl)(morpholino)methanone (23a) ... 77

(5-(Benzo[d][1,3]dioxol-5-yl)-1H-pyrazol-3-yl)(morpholino)methanone (24a) ... 77

Optimization of Conditions

The starting conditions for reaction development were chosen as follows:

NBu_3 (0.27 mmol, 64 µL, 1.0 equiv), 3 mL MeCN, CH_3COOH (0.27 mmol, 16 µL, 1.0 equiv), and ($Ir[dF(CF_3)ppy]_2(dtbpy))PF_6$[1] (0.0054 mmol, 0.006 g, 0.02 equiv) were weighed into a 4 mL glass vial equipped with a stir bar. The vial was sealed and the reaction mixture was stirred for 24 h at room temperature under an air atmosphere while irradiating with blue LEDs (465 nm).

In follow-up experiments, the reaction conditions were changed in order to increase the yield; detailed changes and results are tabulated in tables S1 through S5.

General Procedure for Amide synthesis after optimization (Table S5, Entry 2)

NR_3 (0.27 mmol, 64 µL, 1.0 equiv), 3 mL MeCN, ($Ir[dF(CF_3)ppy]_2(dtbpy))PF_6$ (0.0027 mmol, 0.003 g, 0.01 equiv), CH_3COOH (0.41 mmol, 24 µL, 1.5 equiv) and CF_3SO_2Na (0.41 mmol, 0.063 g, 1.5 equiv) were mixed in a 4 mL glass vial equipped with a stir bar. The vial was sealed and the reaction mixture was stirred for 48 h at room temperature while irradiating with blue LEDs.

To determine crude assay yields by GC, decane or dodecane was added to the reaction mixture. The mixture was sampled by diluting an aliquot with MeCN or EtOAc, followed by filtration, and analysis of the filtrate by GC-FID. Yields were determined by use of a previously established calibration curve.

To determine crude assay yields by quantitative ^1H NMR, the reaction mixture was evaporated. Then, $CDCl_3$ and 1,1,2-trichloroethane or *p*-xylene (as internal standards) were added. The resulting suspension was mixed well, filtered, and analyzed by quantitative ^1H NMR.

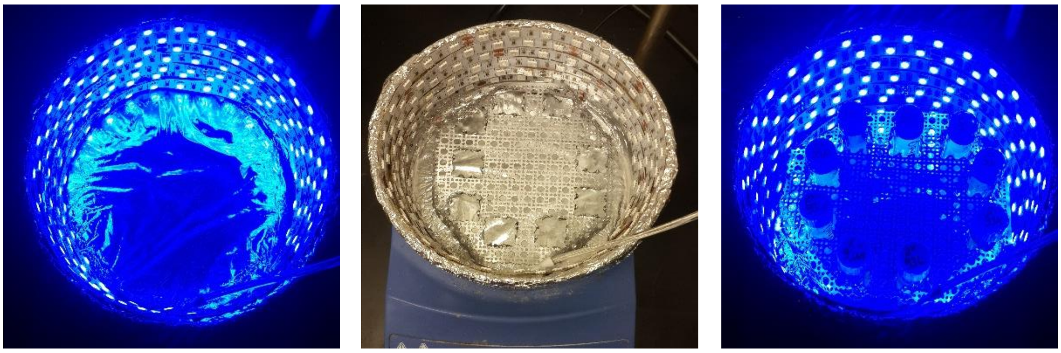

Figure S1. LED reactor and vial setup. Left: The empty reactor upon irradiation, consisting of an aluminum-wrapped oil-bath container made from glass, equipped with LED strips. Middle: LED reactor without irradiation. Right: Reaction vials in metal holder inside of LED reactor with irradiation.

[1] ($Ir[dF(CF_3)ppy]_2(dtbpy))PF_6$ (CAS Number 870987-63-6) used in this study was obtained from Sigma Aldrich (SKU 747793-1G).

Table S1. Optimization of amide synthesis from NBu_3. Starting conditions for development (entry 1), unless otherwise indicated: NBu_3 (0.27 mmol, 64 µL, 1.0 equiv), 3 mL MeCN, CH_3COOH (0.27 mmol, 16 µL, 1.0 equiv) and $(Ir[dF(CF_3)ppy]_2(dtbpy))PF_6$ (0.0054 mmol, 0.006 g, 0.02 equiv), blue leds, r.t., 24 h.

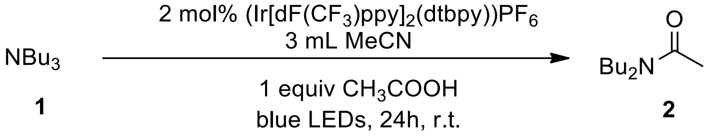

Entry	Changes Compared to Starting Conditions (Entry 1)	Yield (%)[a]
1	--	[b]N.R.
2	1 equiv NaOH	N.R.
3	1 equiv K_3PO_4	N.R.
4	1 equiv K_2CO_3	N.R.
5	1 equiv $TMSCF_3$	[c]N.D.P
6	1 equiv CF_3SO_2Na	46%
7	1.5 equiv CF_3SO_2Na	52%
8	With $Ru(bpy)_3$ instead of $(Ir[dF(CF_3)ppy]_2(dtbpy))PF_6$	<%5
9	With $[Ir(dtbbpy)(ppy)_2]PF_6$ instead of $(Ir[dF(CF_3)ppy]_2(dtbpy))PF_6$	57%
**10**	**1.5 equiv CF_3SO_2Na and 1.5 equiv CH_3COOH**	**86%**

[a]Yields were determined by GC. [b]N.R.= No Reaction. [c]N.D.P.= No Desire Product.

Table S2. Solvent optimization of amide synthesis from NBu_3. Starting conditions for development (Table1/entry 10), unless otherwise indicated: NBu_3 (0.27 mmol, 64 µL, 1.0 equiv), 3 mL MeCN, CH_3COOH (0.41 mmol, 24 µL, 1.5 equiv), CF_3SO_2Na (0.41 mmol, 0.063 g, 1.5 equiv) and $(Ir[dF(CF_3)ppy]_2(dtbpy))PF_6$ (0.0054 mmol, 0.006 g, 0.02 equiv), blue leds, r.t., 24 h.

	Changes Compared to Conditions	Yield (%)[a]
1	with MeOH as solvent	[b]N.R.
2	with DMSO as solvent	N.R.
3	with DMF as solvent	N.R.
4	with IPA as solvent	N.R.

[a]Yields were determined by GC. [b]N.R.= No Reaction

Table S3. Amount of catalyst optimization of amide synthesis from NBu_3. Starting conditions for development (Table1/entry 10), unless otherwise indicated: NBu_3 (0.27 mmol, 64 µL, 1.0 equiv), 3 mL MeCN, CH_3COOH (0.41 mmol, 24 µL, 1.5 equiv), CF_3SO_2Na (0.41 mmol, 0.063 g, 1.5 equiv) and $(Ir[dF(CF_3)ppy]_2(dtbpy))PF_6$ (0.0054 mmol, 0.006 g, 0.02 equiv), blue leds, r.t., 24 h.

	Changes Compared to Conditions	Yield (%)[a]
1	4mol% $(Ir[dF(CF_3)ppy]_2(dtbpy))PF_6$	87%
**2**	**1mol% $(Ir[dF(CF_3)ppy]_2(dtbpy))PF_6$**	**85%**
3	0.5mol% $(Ir[dF(CF_3)ppy]_2(dtbpy))PF_6$	64%

[a]Yields were determined by GC. [b]N.R.= No Reaction

Table S4. Reaction time optimization of of amide synthesis from NBu_3. Starting conditions for development (Table3/entry 2), unless otherwise indicated: NBu_3 (0.27 mmol, 64 µL, 1.0 equiv), 3 mL MeCN, CH_3COOH (0.41 mmol, 24 µL, 1.5 equiv), CF_3SO_2Na (0.41 mmol, 0.063 g, 1.5 equiv) and (Ir[dF(CF_3)ppy]$_2$(dtbpy))PF_6 (0.0054 mmol, 0.006 g, 0.02 equiv), blue leds, r.t., 24 h.

	**Changes Compared to Conditions**	**Yield (%)[a]**
1	12h	72%
2	6h	57%
**3**	**48h**	**94%**
[a]Yields were determined by GC. [b]N.R.= No Reaction		

Table S5. Control reactions for mechanistic studies of N-dealkylation of NBu_3. For the best conditions, unless otherwise indicated: NBu_3 (0.27 mmol, 64 µL, 1.0 equiv), 3 mL MeCN, CH_3COOH (0.41 mmol, 24 µL, 1.5 equiv), CF_3SO_2Na (0.41 mmol, 0.063 g, 1.5 equiv) and (Ir[dF(CF_3)ppy]$_2$(dtbpy))PF_6 (0.0054 mmol, 0.006 g, 0.02 equiv), blue leds, r.t., 48h.

	**Changes Compared to Conditions**	**Yield (%)[a]**
1	---	94%
2	No (Ir[dF(CF_3)ppy]$_2$(dtbpy))PF_6	N.R.
3	No CF_3SO_2Na	N.R.
4	No CH_3COOH	N.R.
5	With 0.5 equiv CF_3SO_2Na	38%
6	50 mol% BHT[b]	82%
7	50 mol% TEMPO	84%
8	3 equiv TEMPO	N.R.
9	No light	N.R.
10	N_2 atmosphere (no air)	14%
[a]Yields were determined by GC. [b]N.R.= No Reaction		

Selected GCMS spectra of crude reaction mixtures during optimization studies

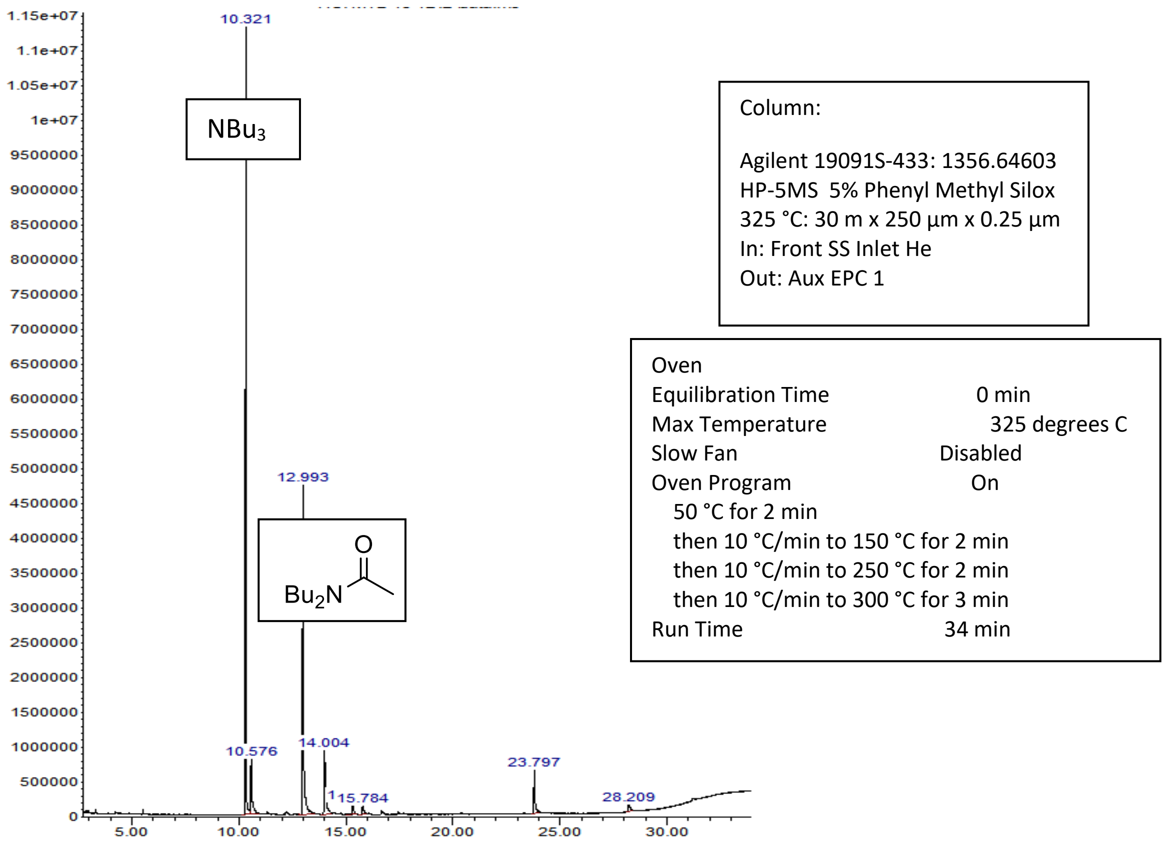

Figure S2. GCMS Chromatogram and peak assignments for crude reaction in Table S1/Entry 6.

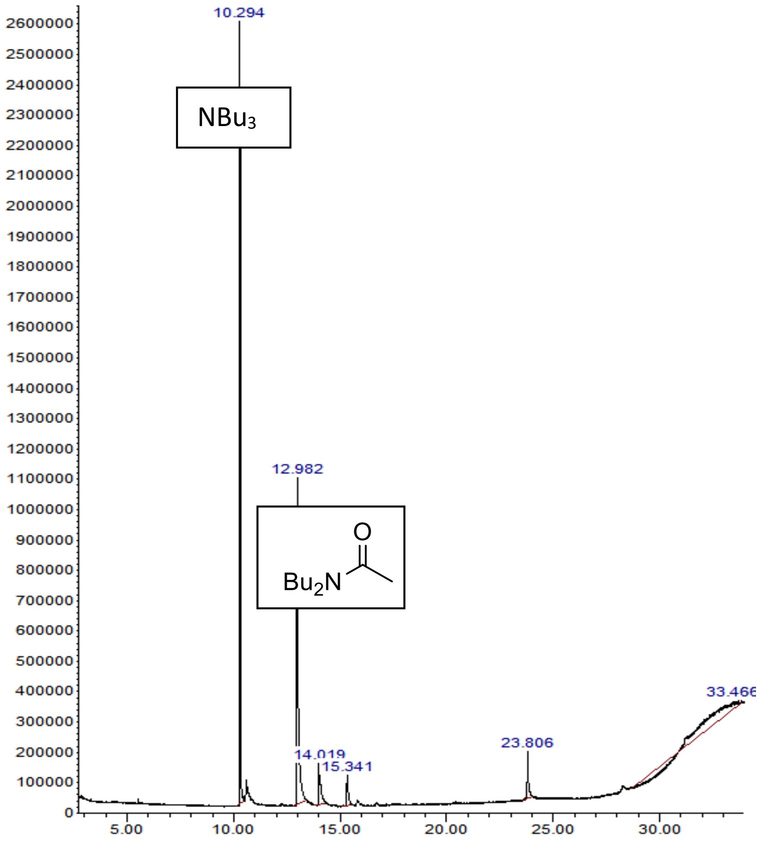

Figure S3. GCMS Chromatogram and peak assignments for crude reaction in Table S1/Entry 7.

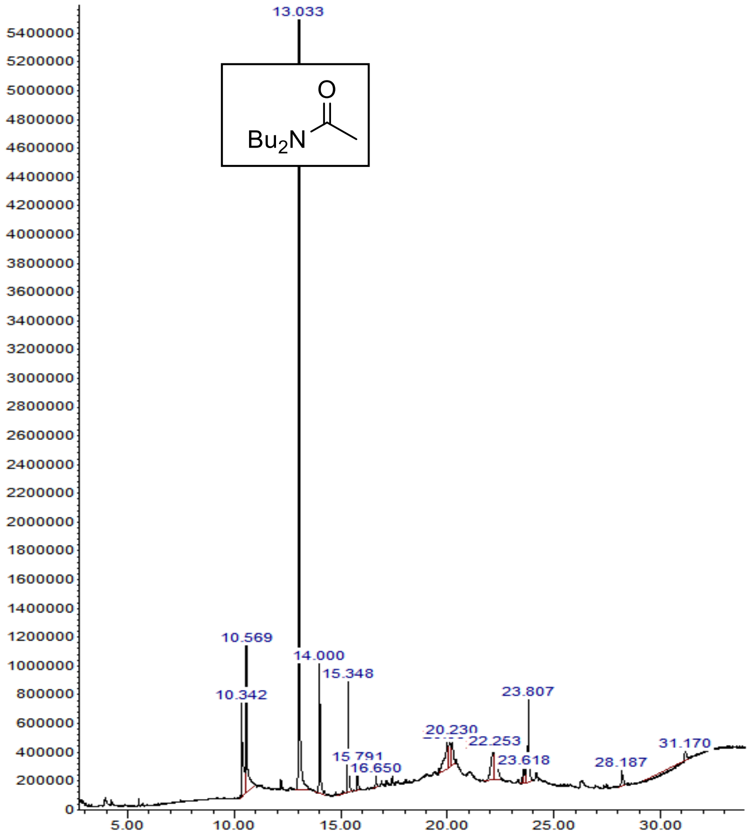

Figure S4. GCMS Chromatogram and peak assignments for crude reaction in Table S3/Entry 3.

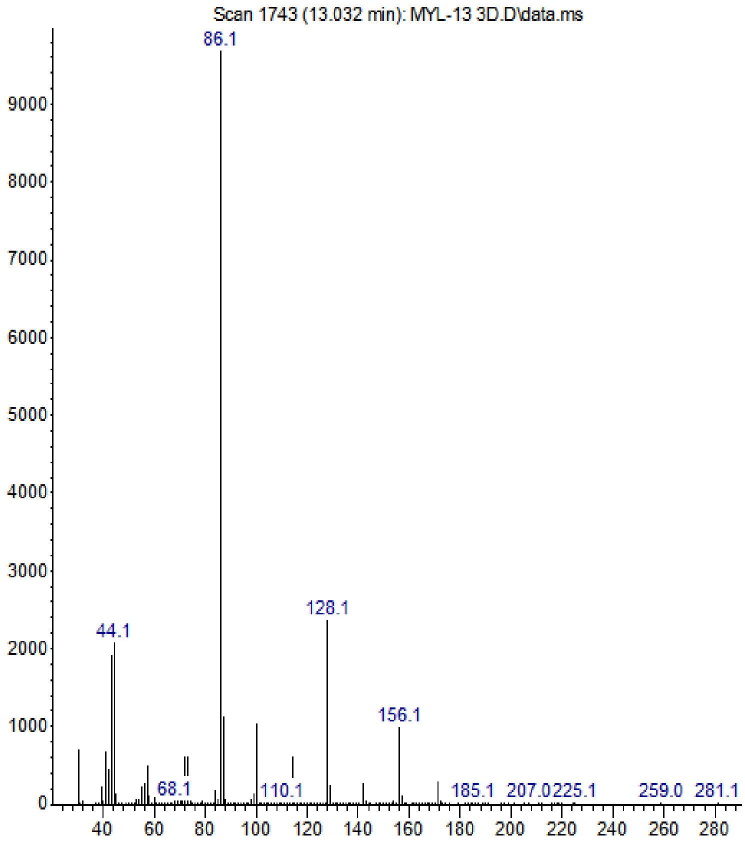

Figure S5. MS Spectrum derived from peak at 13.03 min.

Tabular summary of substrate scope results

Table S6. Substrate scope for reactions of tertiary amines with acetic acid. General Conditions: Amine (0.27 mmol), 3 mL MeCN, ***CH_3COOH*** *(0.41 mmol, 24 µL, 1.5 equiv), CF_3SO_2Na (0.41 mmol, 0.063 g, 1.5 equiv) and (Ir[dF(CF_3)ppy]$_2$(dtbpy))PF_6 (0.0054 mmol, 0.006 g, 0.02 equiv), blue LEDs, r.t., 48 h.*

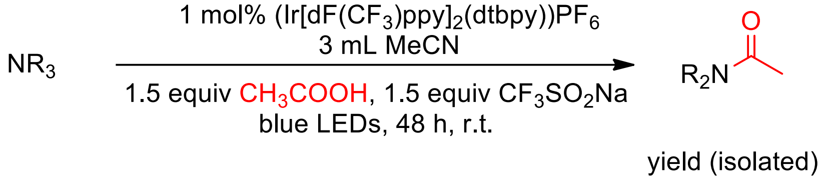

Substrate	Product(s)	Yield[a] (Isolated Yield)
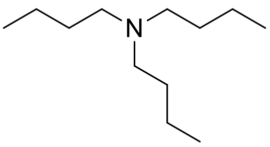 **1**	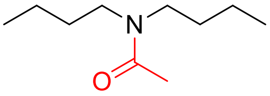 **1a**	94% (88%)
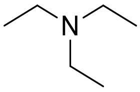 **2**	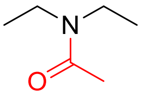 **2a**	92%
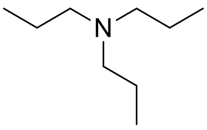 **3**	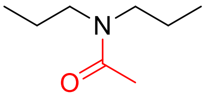 **3a**	89% (85%)
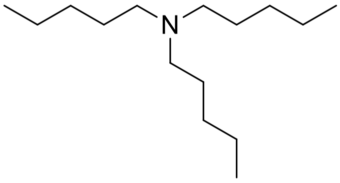 **4**	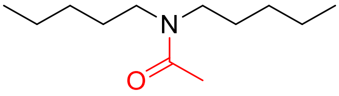 **4a**	88% (86%)
$NHex_3$ **5**	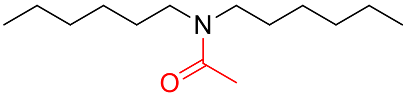 **5a**	91% (85%)
N(n-octyl)$_3$ **6**	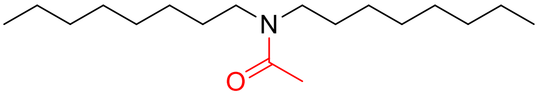 **6a**	90% (87%)

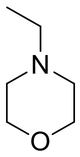 **7**	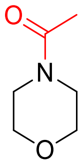 **7a**	67% (64%)
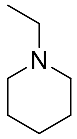 **8**	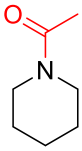 **8a**	74% (71%)

[a]Yields were determined by quantitative, crude ^{1}H NMR using *p*-xylene as internal standard or by GC, using decanal or dodecanal as internal standards.

*Table S7. Substrate scope for reactions of tertiary amines with propanoic acid. General Conditions: Amine (0.27 mmol), 3 mL MeCN, **CH_3CH_2COOH** (0.41 mmol, 30 µL, 1.5 equiv), CF_3SO_2Na (0.41 mmol, 0.063 g, 1.5 equiv) and (Ir[dF(CF_3)ppy]$_2$(dtbpy))PF_6 (0.0054 mmol, 0.006 g, 0.02 equiv), blue LEDs, r.t., 48 h.*

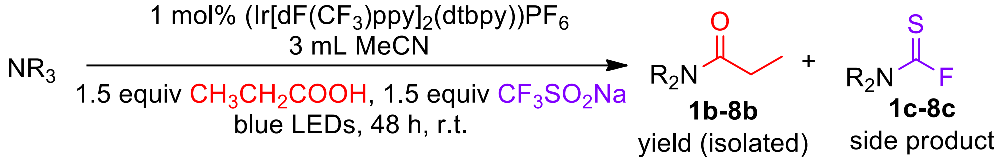

Substrate	Product(s)	Yield[a] (Isolated Yield)
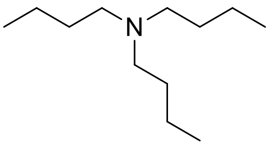 1	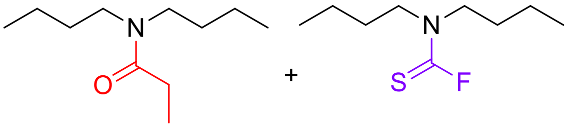 1b + 1c	63% (61%) 78%[b] **1b** 37% (35%) 18%[b] **1c**
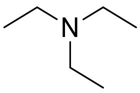 2	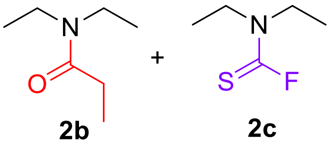 2b + 2c	44% **2b** 49% **2c**
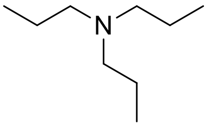 3	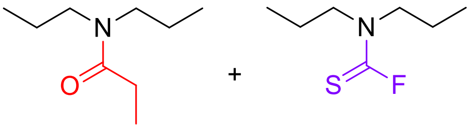 3b + 3c	58% (56%) **3b** 40% (38%) **3c**
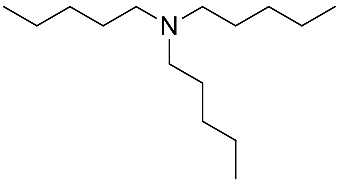 4	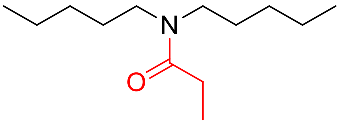 4b + 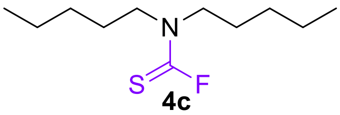 4c	52% (49%) **4b** 46% (43%) **4c**
N(n-Hex)$_3$ **5**	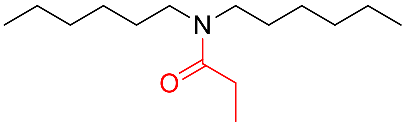 5b + 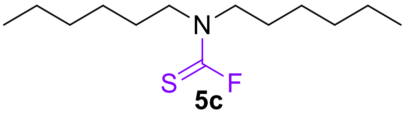 5c	71% (68%) **5b** 26% (24%) **5c**

N(n-octyl)$_3$<br><br>**6**	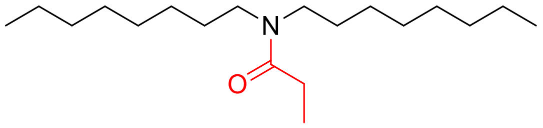<br>**6b**<br>+<br>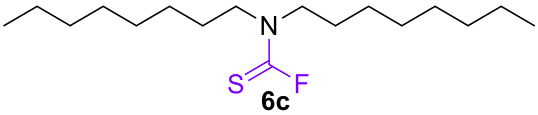<br>**6c**	62% (59%)<br>**6b**<br><br>36% (35%)<br>**6c**
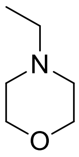<br>**7**	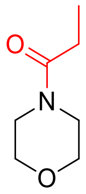 + 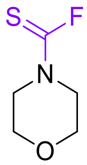<br>**7b** **7c**	No desired product (**7b**) was obtained. A small amount of starting material **7** was observed. Other complex side products.
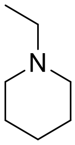<br>**8**	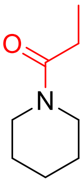 + 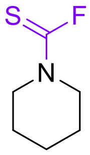<br>**8b** **8c**	69% (65%)<br>**8b**<br><br>29% (27%)<br>**8c**

[a]Yields were determined by quantitative, crude ^{1}H NMR using *p*-xylene as internal standard or by GC, using decanal or dodecanal as internal standards. [b]With 3 equiv CH_3CH_2COOH.

*Table S8. Substrate scope for reactions of tertiary amines with ibuprofen. General Conditions: Amine (0.27 mmol), 3 mL MeCN, **(S)-(+)-Ibuprofen** (0.41 mmol, 0.083 g, 1.5 equiv), CF_3SO_2Na (0.41 mmol, 0.063 g, 1.5 equiv) and (Ir[dF(CF_3)ppy]$_2$(dtbpy))PF_6 (0.0054 mmol, 0.006 g, 0.02 equiv), blue LEDs, r.t., 48h.*

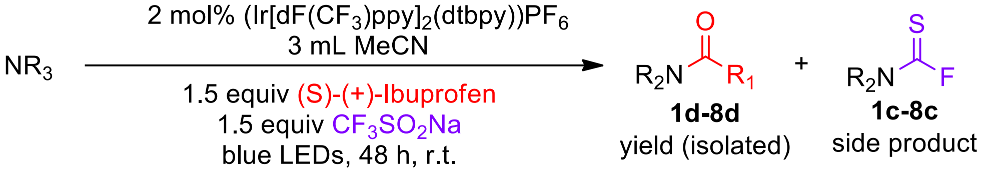

Substrate	Product(s)	Yield[a] (Isolated Yield)
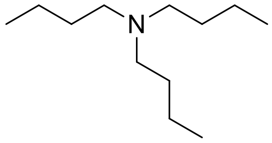 **1**	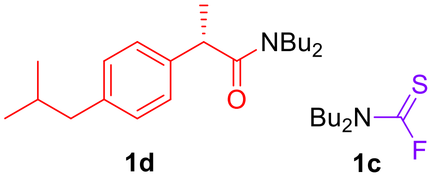 **1d** **1c**	70% (67%) **1d** 28% **1c**
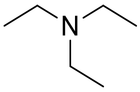 **2**	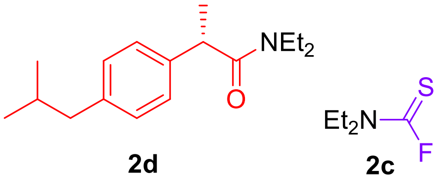 **2d** **2c**	59% (56%) **2d** 39% **1c**
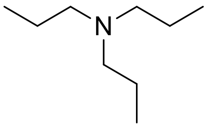 **3**	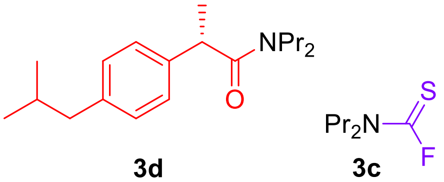 **3d** **3c**	68% (67%) **3d** 31% **3c**
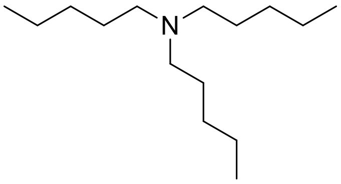 **4**	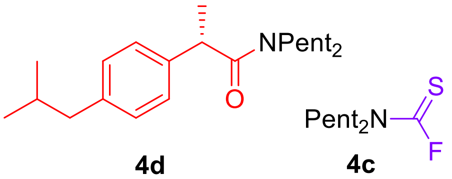 **4d** **4c**	56% (52%) **4d** 43% **4c**
N(n-Hex)$_3$ **5**	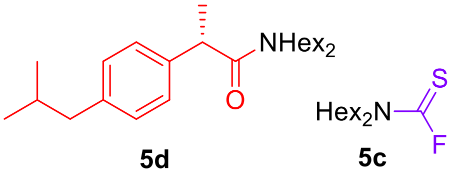 **5d** **5c**	74% (70%) **5d** 25% **5c**
N(n-octyl)$_3$ **6**	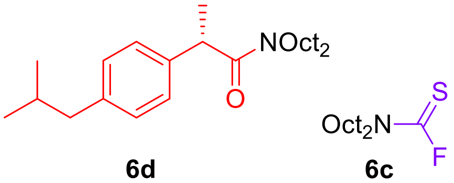 **6d** **6c**	71% (69%) **6d** 27% **6c**

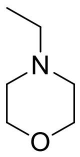 **7**	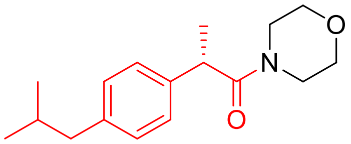 **7d** 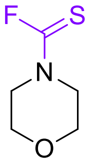 **7c**	77% (74%) **7d**<br>21% **7c**
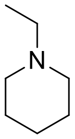 **8**	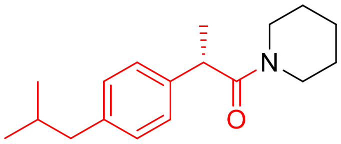 **8d** 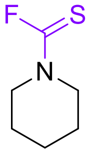 **8c**	51% (48%) **8d**<br>47% **8c**

[a] Yields were determined by quantitative, crude ^{1}H NMR using *p*-xylene as internal standard or by GC, using decanal or dodecanal as internal standards.

Table S9. Substrate scope for reactions of N-methylmorpholine with complex carboxylic acids. The results were investigated via UPLCMS analysis and are documented as liquid chromatography area percent (LCAP) of product, using the extracted UV trace at 254 nm.

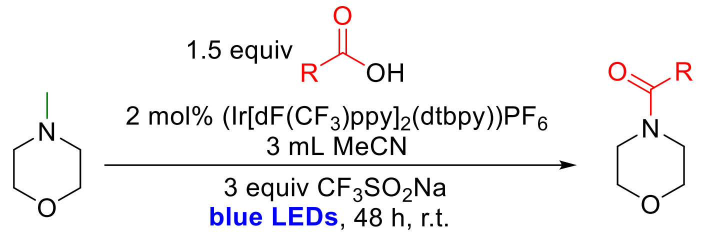

Carboxylic Acid Substrates	Product	Yield[a]
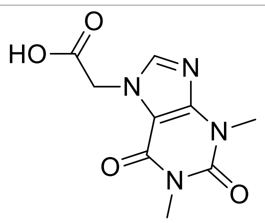 **11**	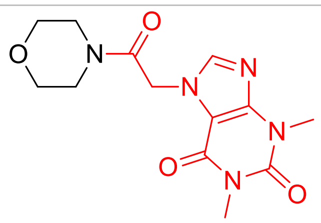 **11a**	22%
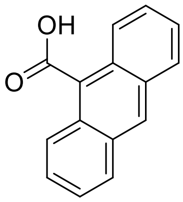 **12**	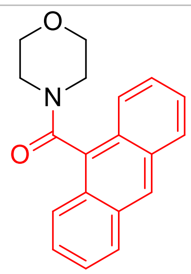 **12a**	18%
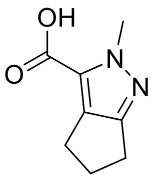 **13**	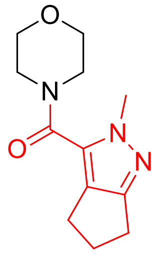 **13a**	24%
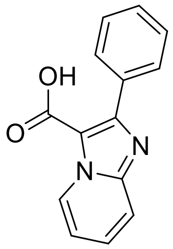 **14**	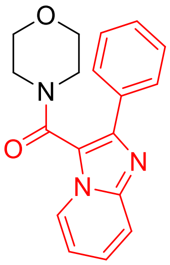 **14a**	29%

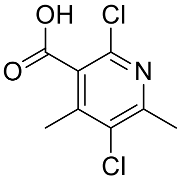 **15**	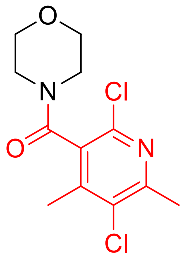 **15a**	57%
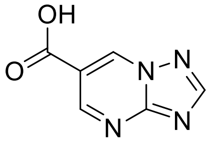 **16**	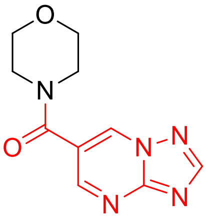 **16a**	9%
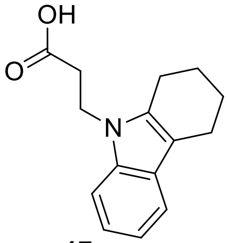 **17**	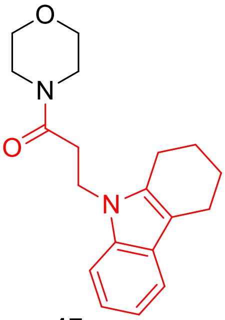 **17a**	28%
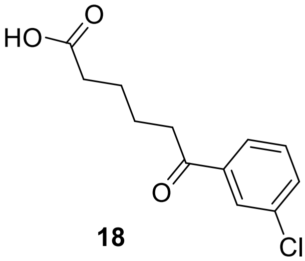 **18**	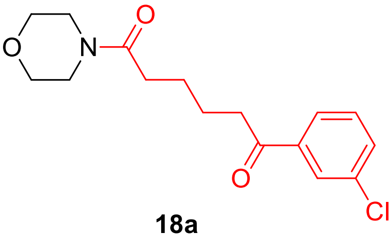 **18a**	71%
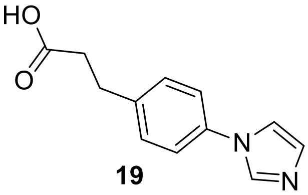 **19**	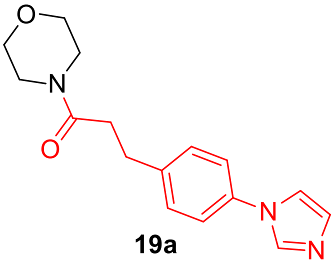 **19a**	73%
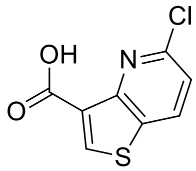 **20**	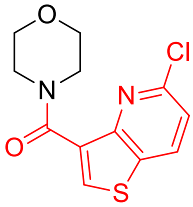 **20a**	66%

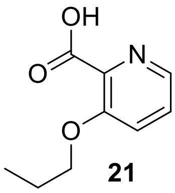 **21**	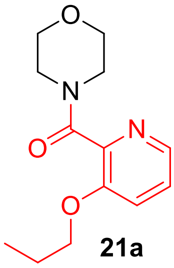 **21a**	25%
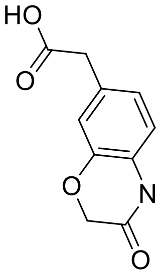 **22**	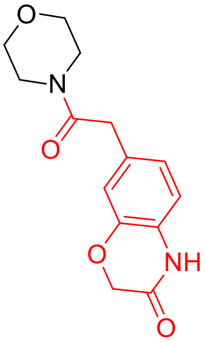 **22a**	15%
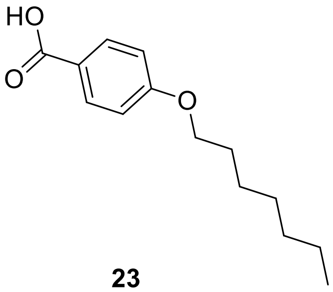 **23**	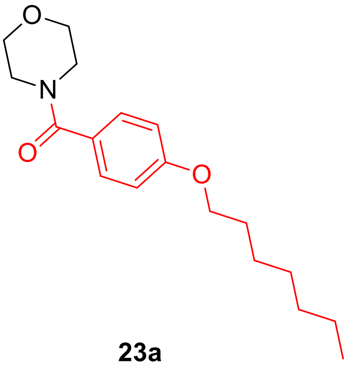 **23a**	67%
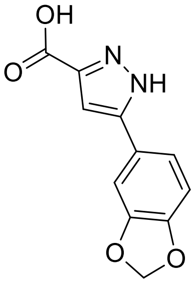 **24**	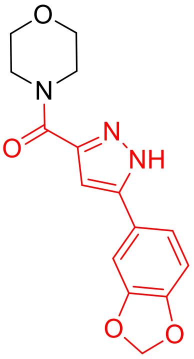 **24a**	66%

[a]Yields are presented as LCAP (liquid chromatography area percent) at 254 nm.

NMR and GCMS spectra of products

Literature-known compounds. Graphics of ^{1}H-NMR spectra are provided below for molecules known in the literature. The data for these ^{1}H NMR spectra are detailed in the Experimental Section of the manuscript. The data were compared with data in the literature and found to be in agreement; relevant literature references for those molecules are also provided directly in the Experimental Section of the manuscript.

Compounds not known in the literature. ^{1}H, ^{13}C, ^{19}F NMR and HRMS measurements were obtained for newly synthesized molecules that are unknown in the literature. The data are available in the manuscript's experimental section. Graphics for NMR as well as HRMS spectra are provided below.

N,N-dibutylacetamide ($C_{10}H_{21}NO$, 1a)

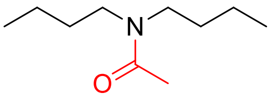

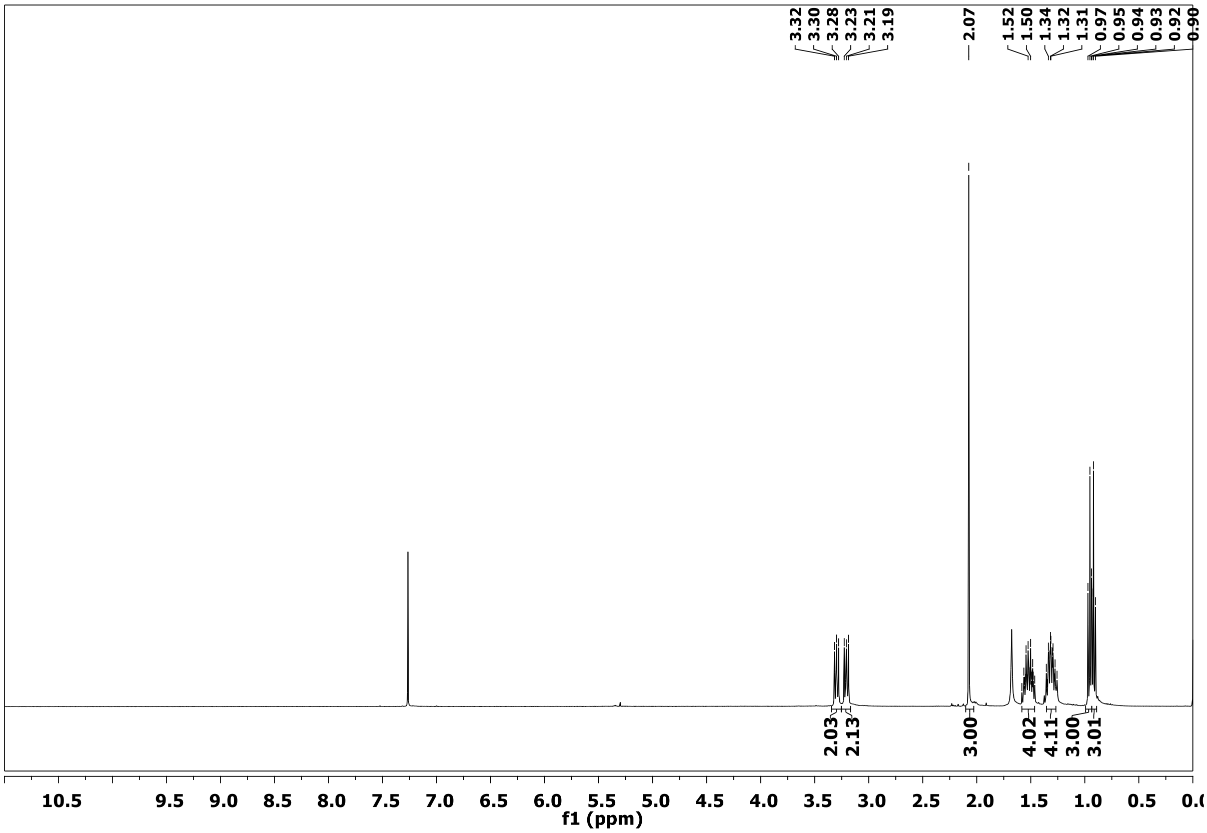

*Figure S6. ^{1}H NMR of N,N-dibutylacetamide **(1a)** ($CDCl_3$), 400 MHz.*

N,N-diethylacetamide ($C_6H_{13}NO$, 2a)

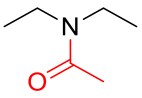

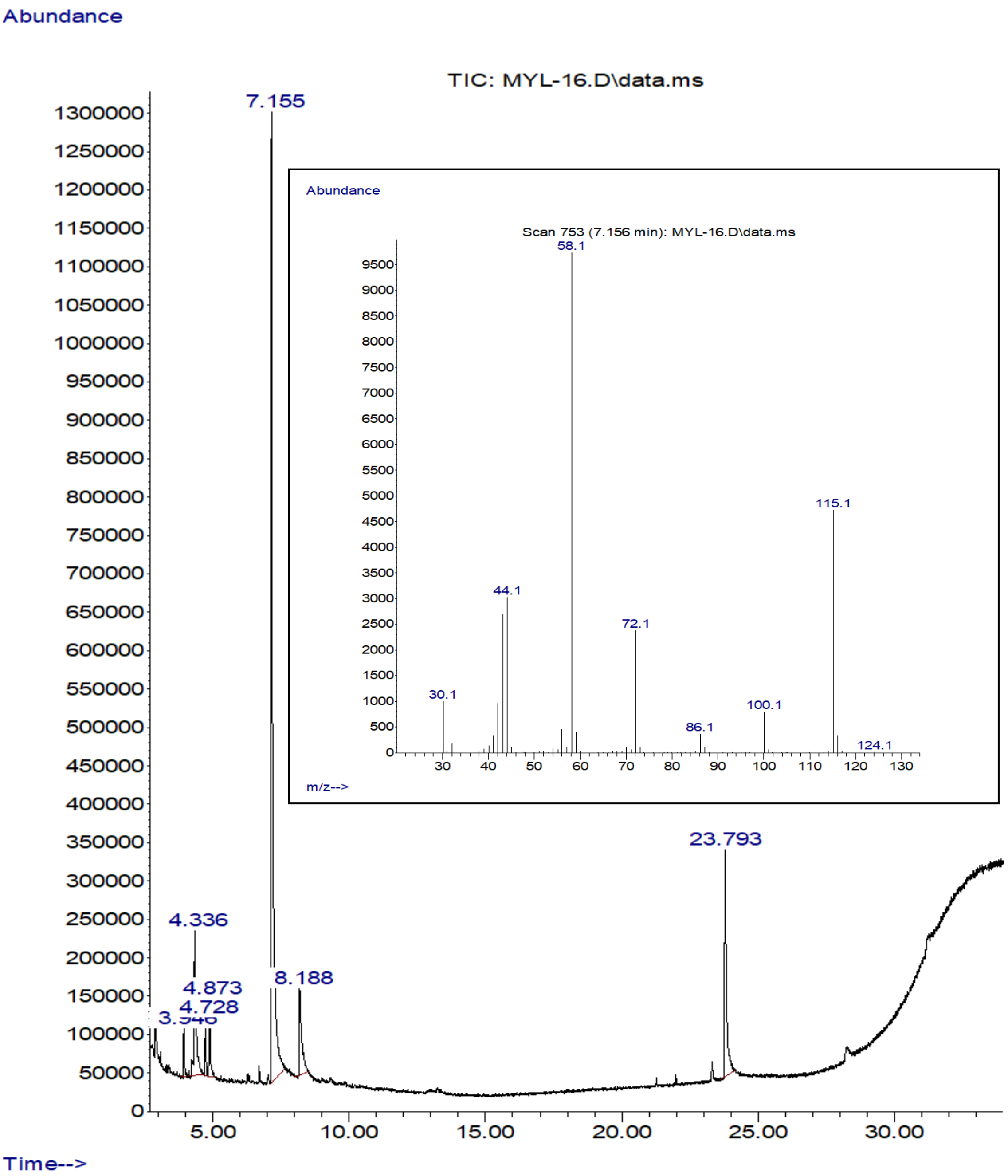

*Figure S7. GCMS Chromatogram and peak assignments for crude reaction NEt_3 (**3**) with CH_3COOH.*

N,N-dipropylacetamide ($C_8H_{17}NO$, 3a)

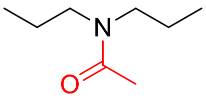

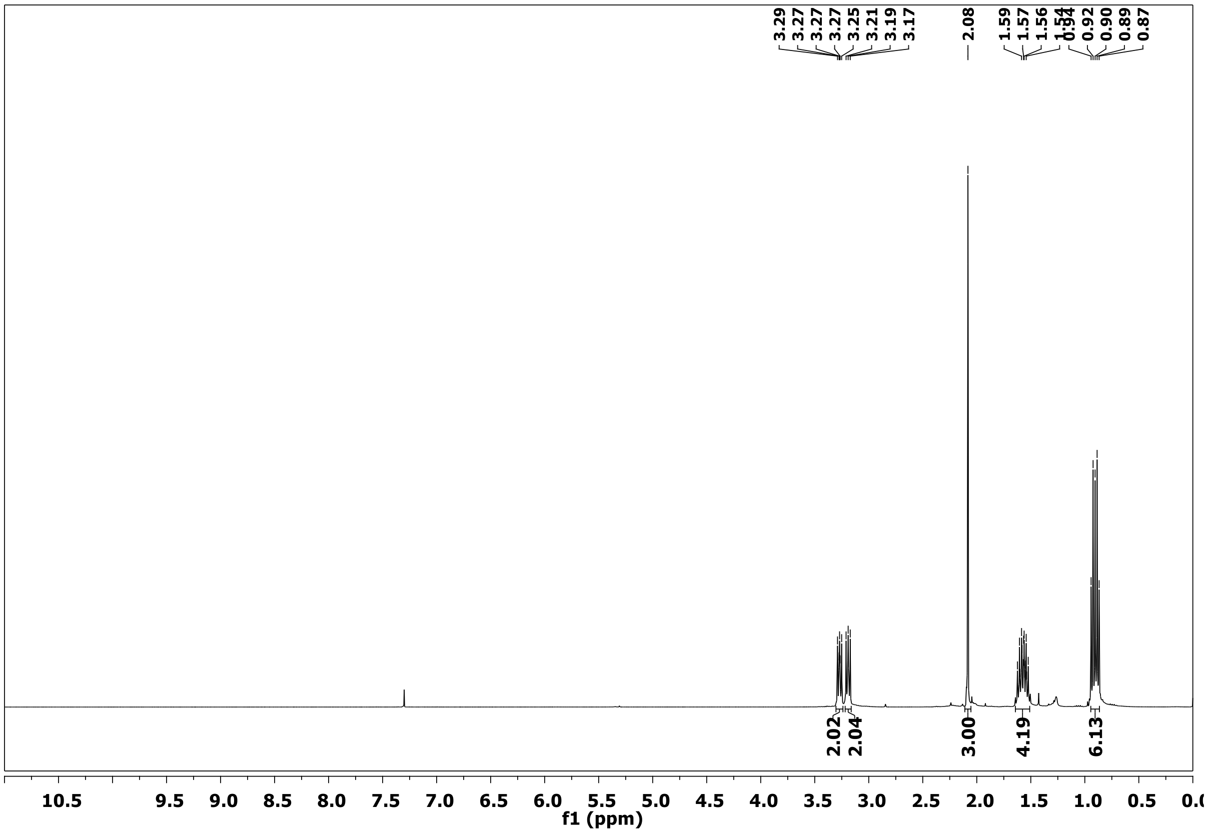

*Figure S8. ^{1}H NMR of N,N-dipropylacetamide **(1a)** ($CDCl_3$), 400 MHz.*

N,N-dipentylacetamide ($C_{12}H_{25}NO$, 4a)

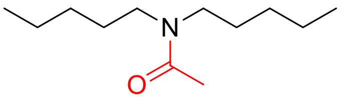

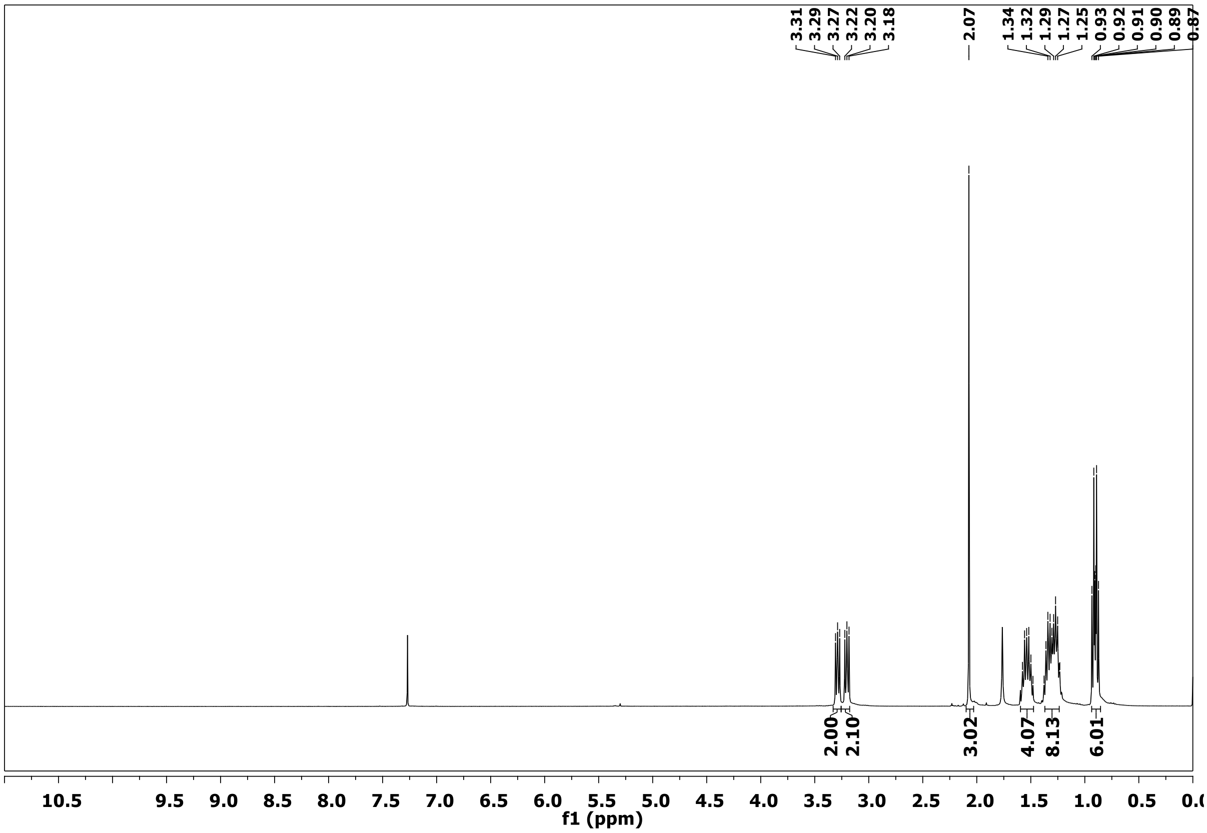

*Figure S9. ¹H NMR of N,N-dipentylacetamide **(4a)** ($CDCl_3$), 400 MHz.*

N,N-dihexylacetamide ($C_{14}H_{29}NO$, 5a)

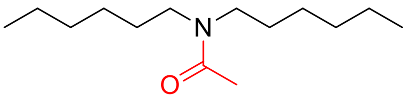

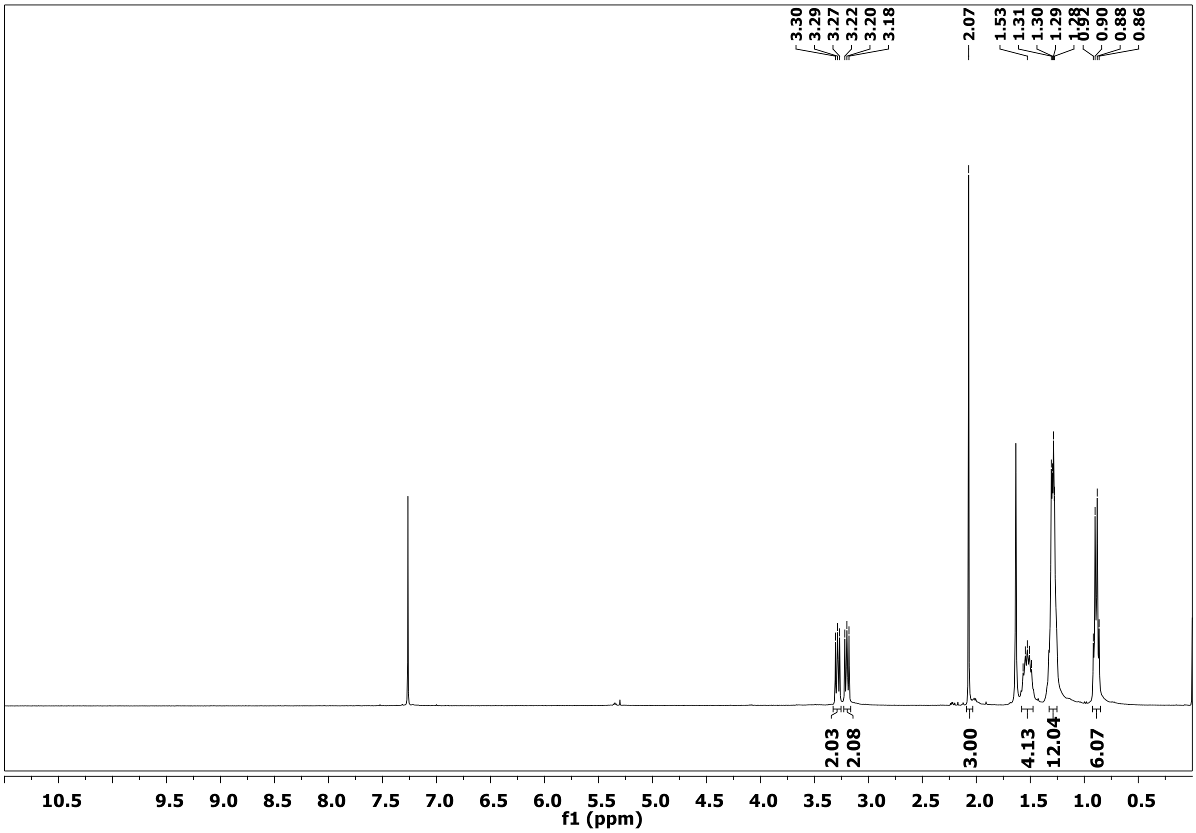

Figure S10. ^{1}H NMR of N,N-dihexylacetamide ***(5a)*** *($CDCl_3$), 400 MHz.*

N,N-dioctylacetamide ($C_{18}H_{37}NO$, 6a)

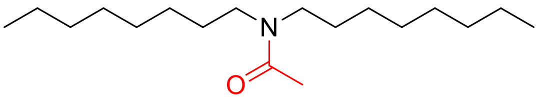

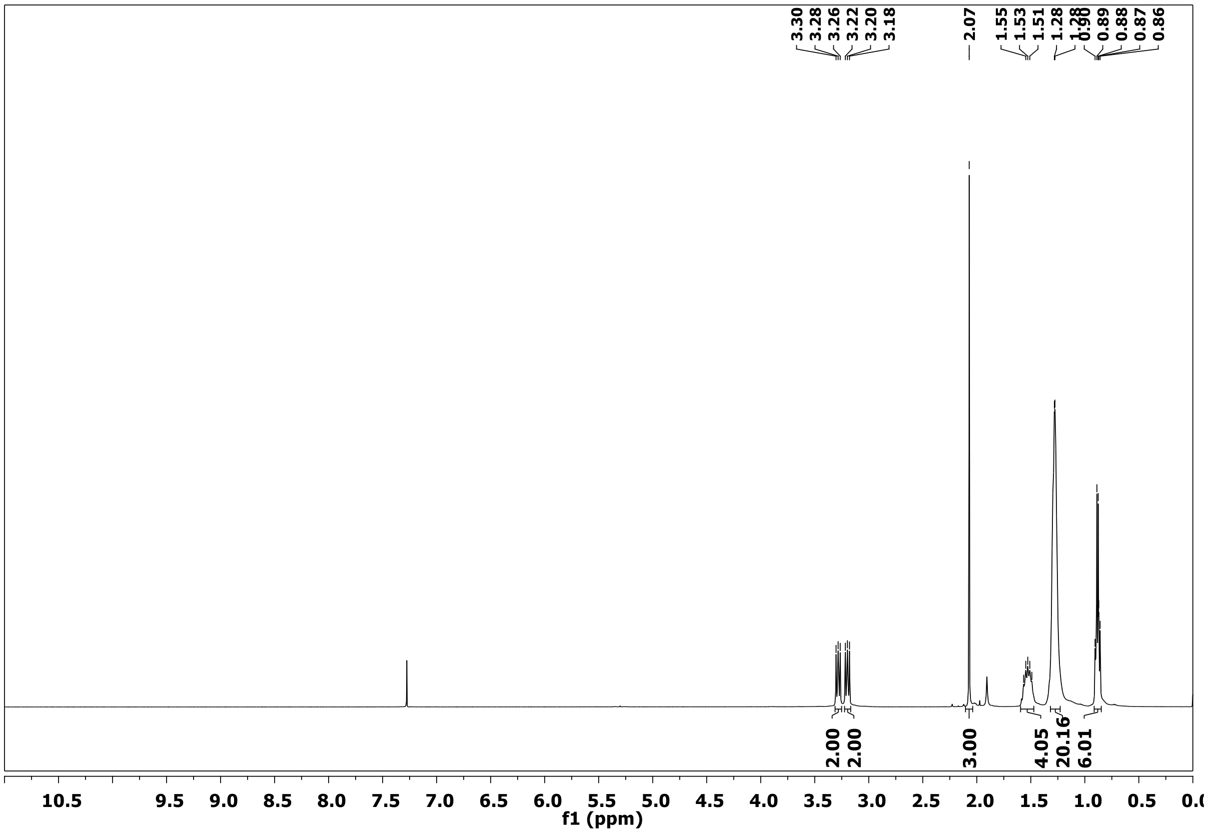

*Figure S11. ^{1}H NMR of N,N-dioctylacetamide **(6a)** (CDCl$_3$), 400 MHz.*

1-morpholinoethanone ($C_6H_{11}NO_2$, 7a)

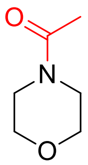

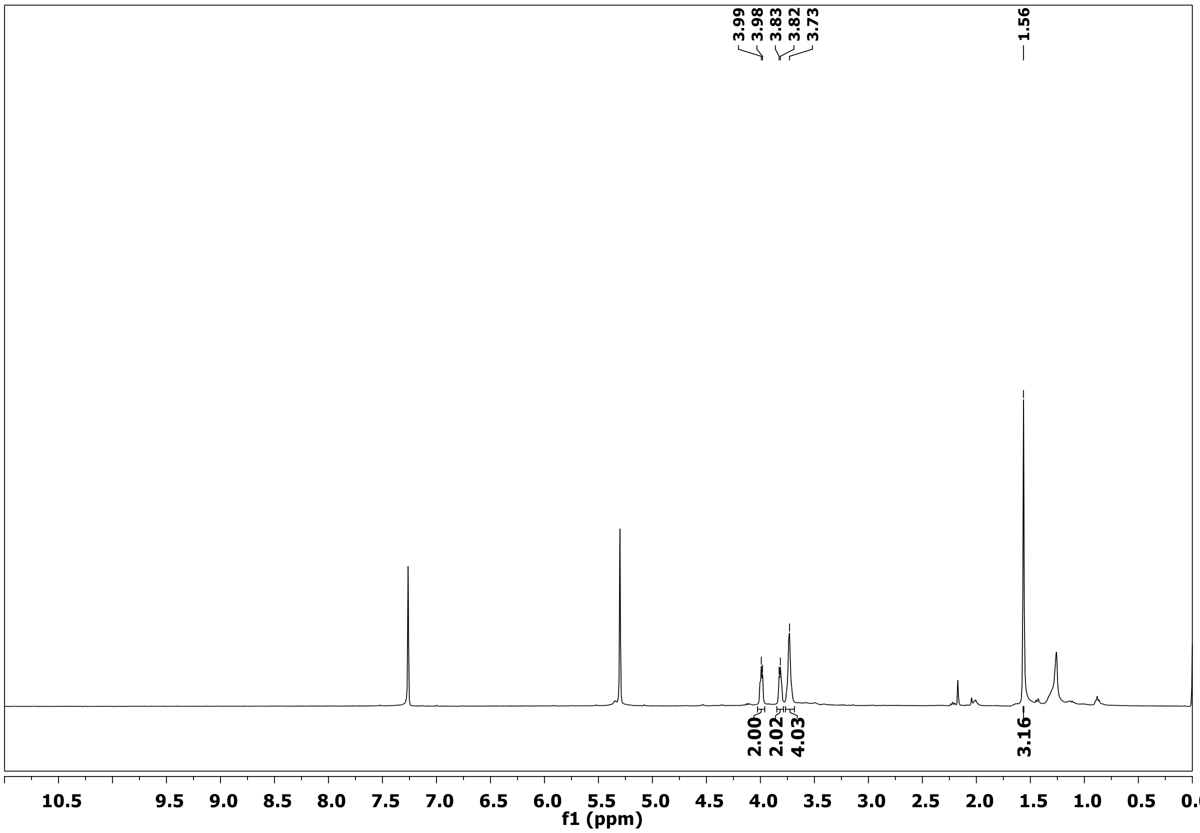

Figure S12. ^{1}H NMR of 1-morpholinoethanone ***(7a)*** *($CDCl_3$), 400 MHz.*

1-(piperidin-1-yl)ethanone ($C_7H_{13}NO$, 8a)

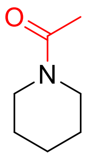

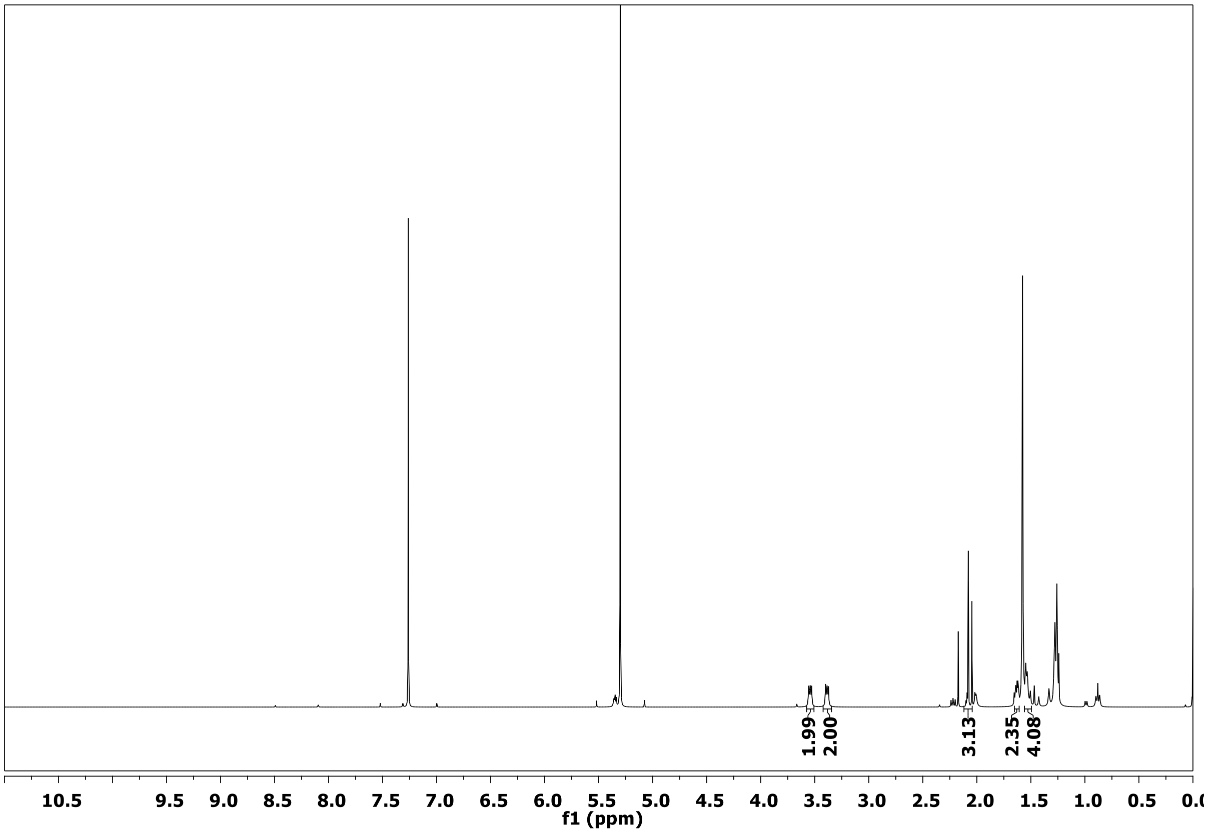

*Figure S13. ^1H NMR of 1-(piperidin-1-yl)ethanone **(8a)** ($CDCl_3$), 400 MHz.*

N,N-dibutylpropionamide ($C_{11}H_{23}NO$, 1b)

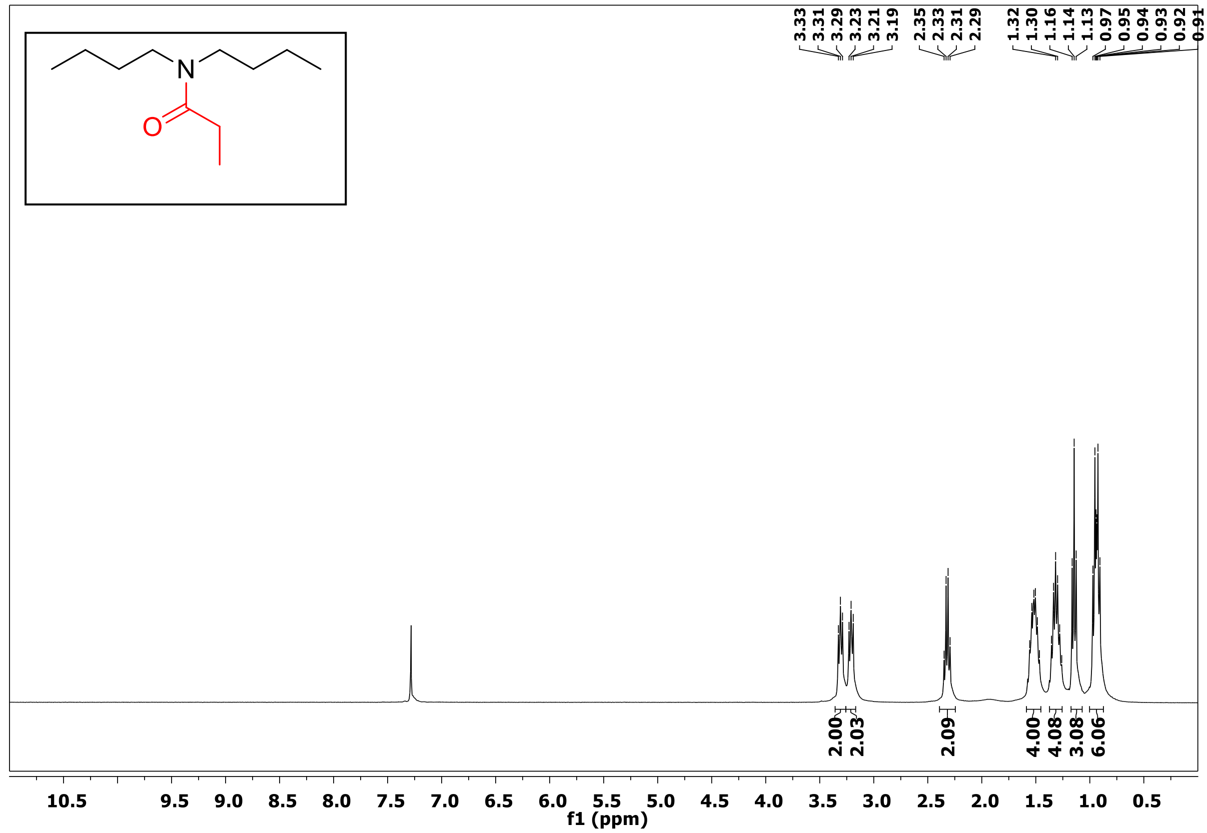

*Figure S14. ^{1}H NMR of N,N-dibutylpropionamide **(1b)** ($CDCl_3$), 400 MHz.*

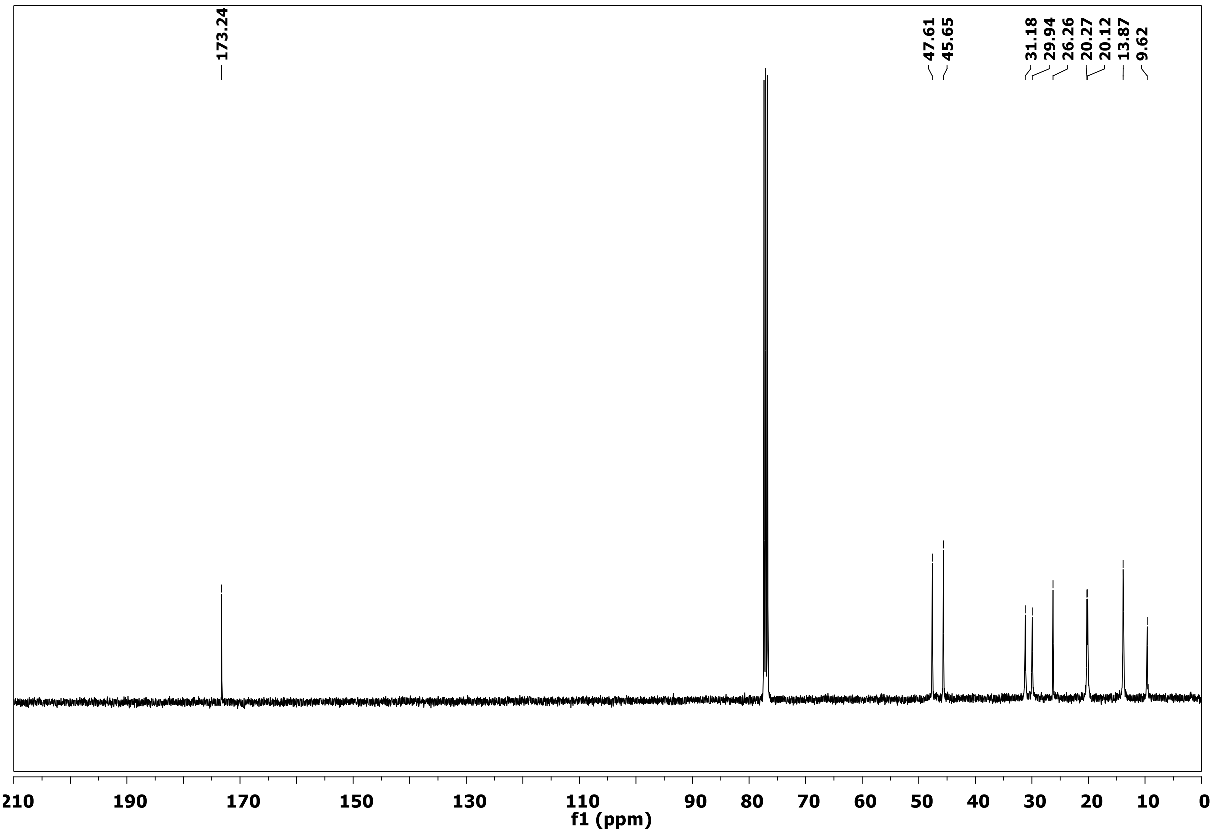

*Figure S15. $^{13}C\{H\}$ NMR of N,N-dibutylpropionamide **(1b)** ($CDCl_3$), 101 MHz.*

N,N-diethylpropionamide ($C_7H_{15}NO$, 2b)

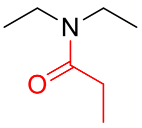

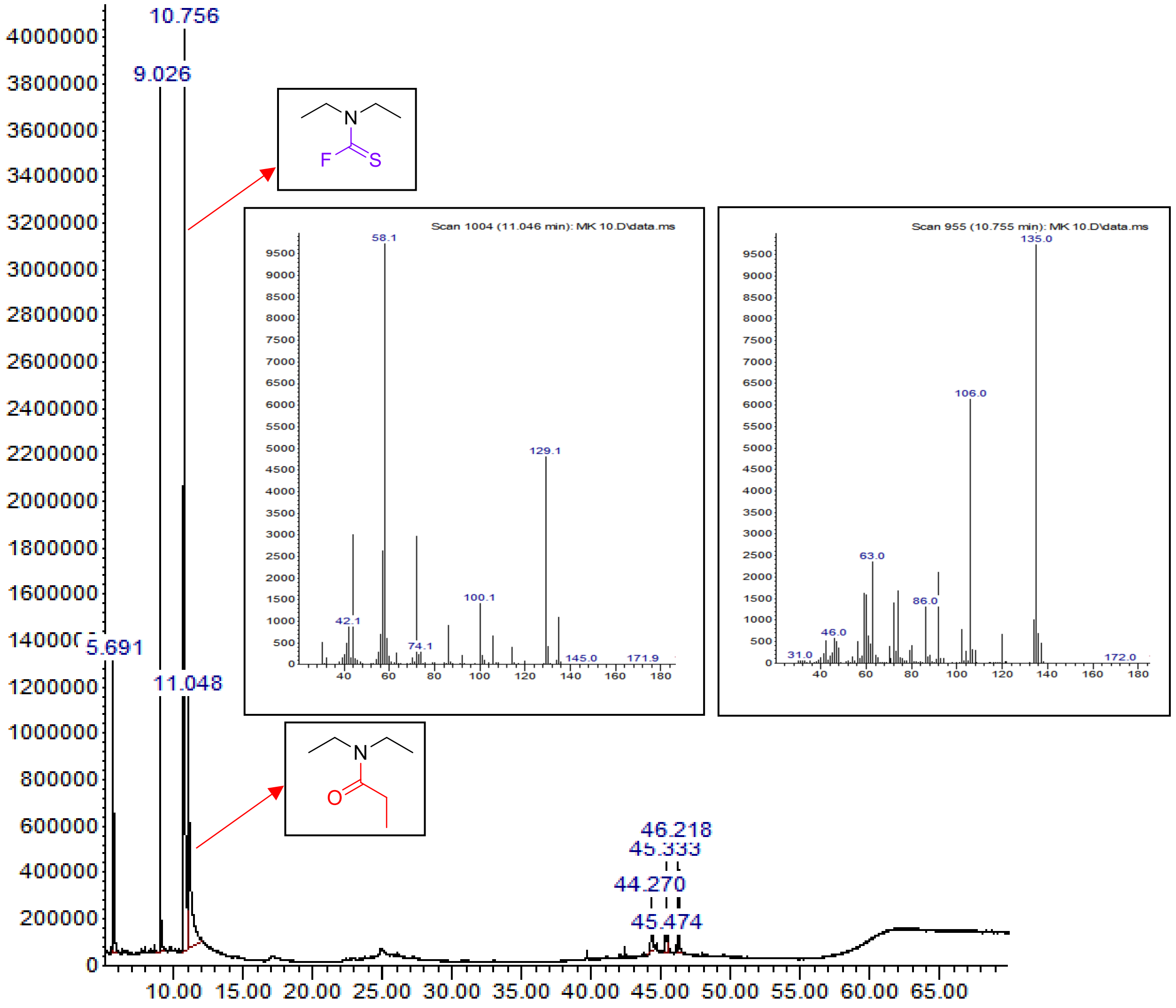

*Figure S16. GCMS Chromatogram and peak assignments for crude reaction NEt_3 (**3**) with propanoic acid.*

N,N-dipropylpropionamide ($C_9H_{19}NO$, 3b)

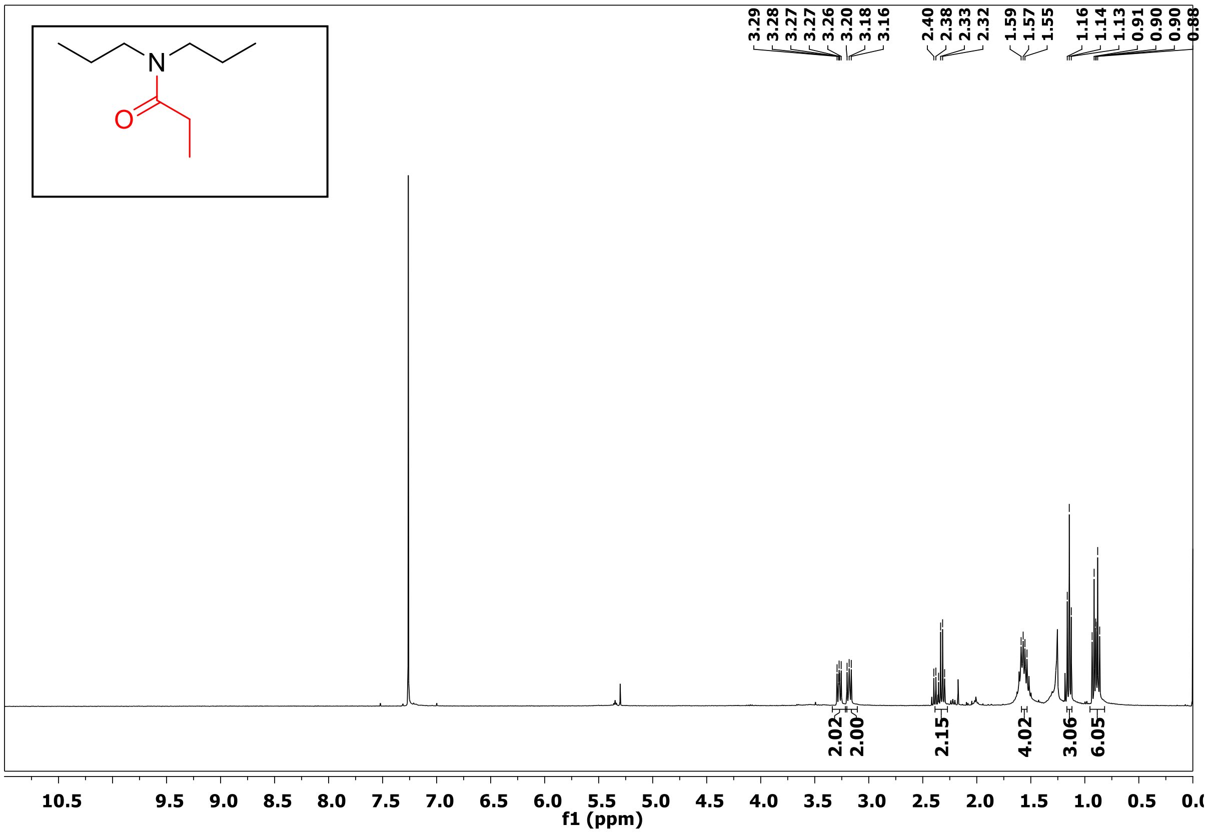

*Figure S17. ¹H NMR of N,N-dipropylpropionamide **(3b)** (CDCl₃), 400 MHz.*

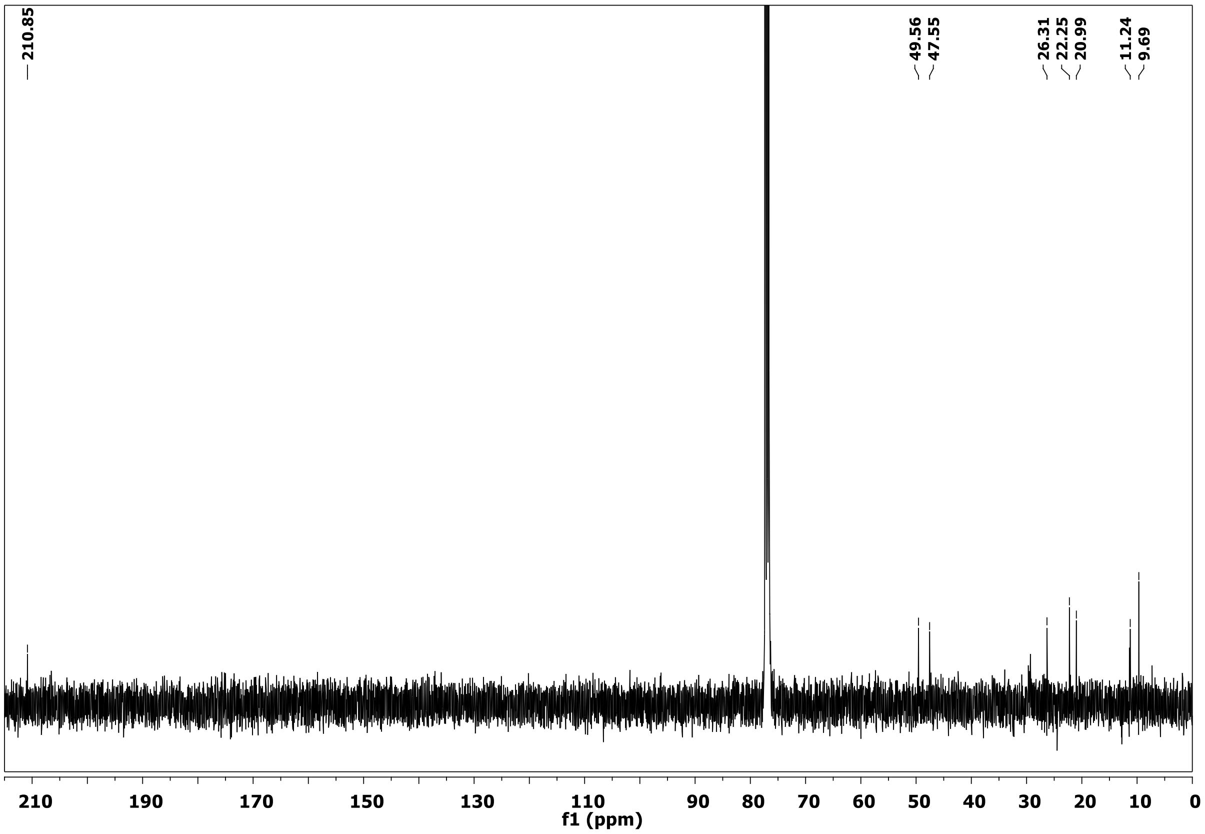

*Figure S18. ^{13}C{H} NMR of N,N-dipropylpropionamide **(3b)** ($CDCl_3$), 101 MHz.*

N,N-dipentylpropionamide ($C_{13}H_{27}NO$, 4b)

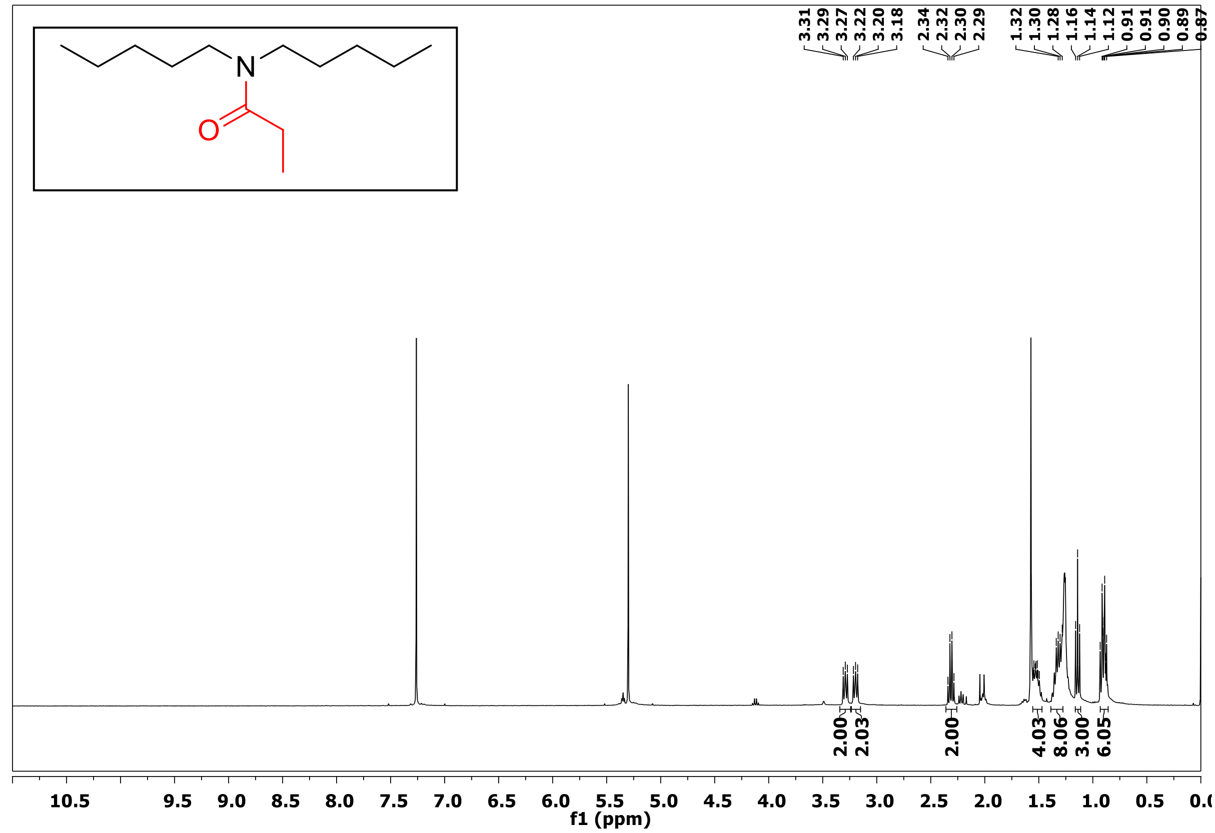

*Figure S19. ^{1}H NMR of N,N-dipentylpropionamide **(4b)** ($CDCl_3$), 400 MHz.*

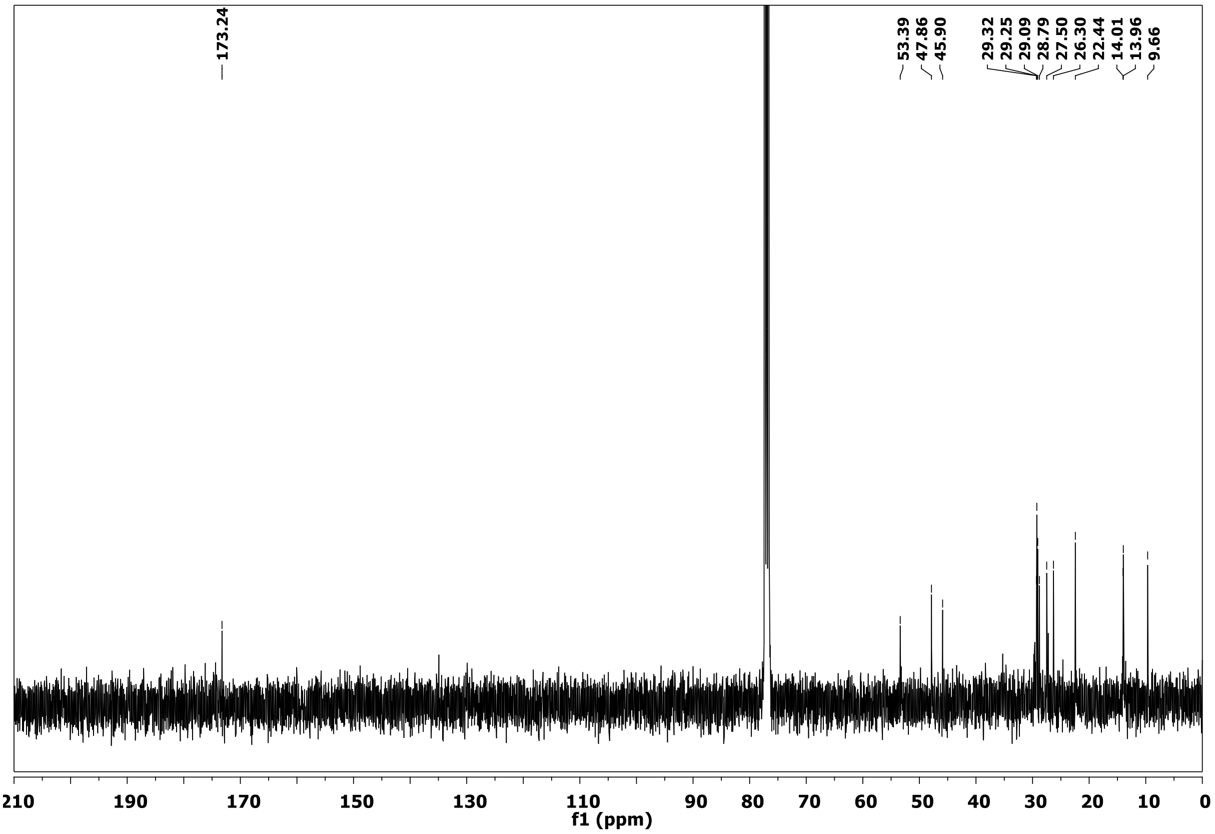

*Figure S20. ^{13}C{H} NMR of N,N-dipentylpropionamide **(4b)** ($CDCl_3$), 101 MHz.*

N,N-dihexylpropionamide ($C_{15}H_{31}NO$, 5b)

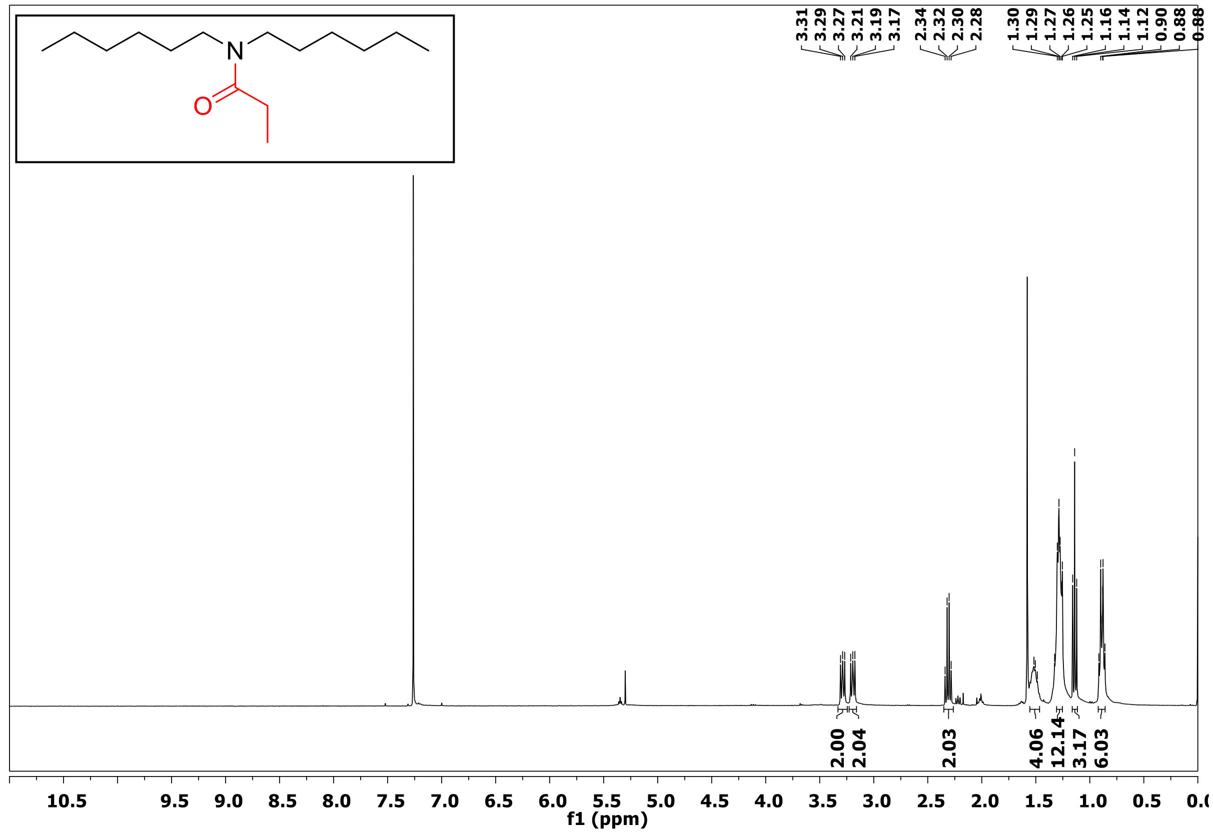

*Figure S21. ^{1}H NMR of N,N-dihexylpropionamide **(5b)** ($CDCl_3$), 400 MHz.*

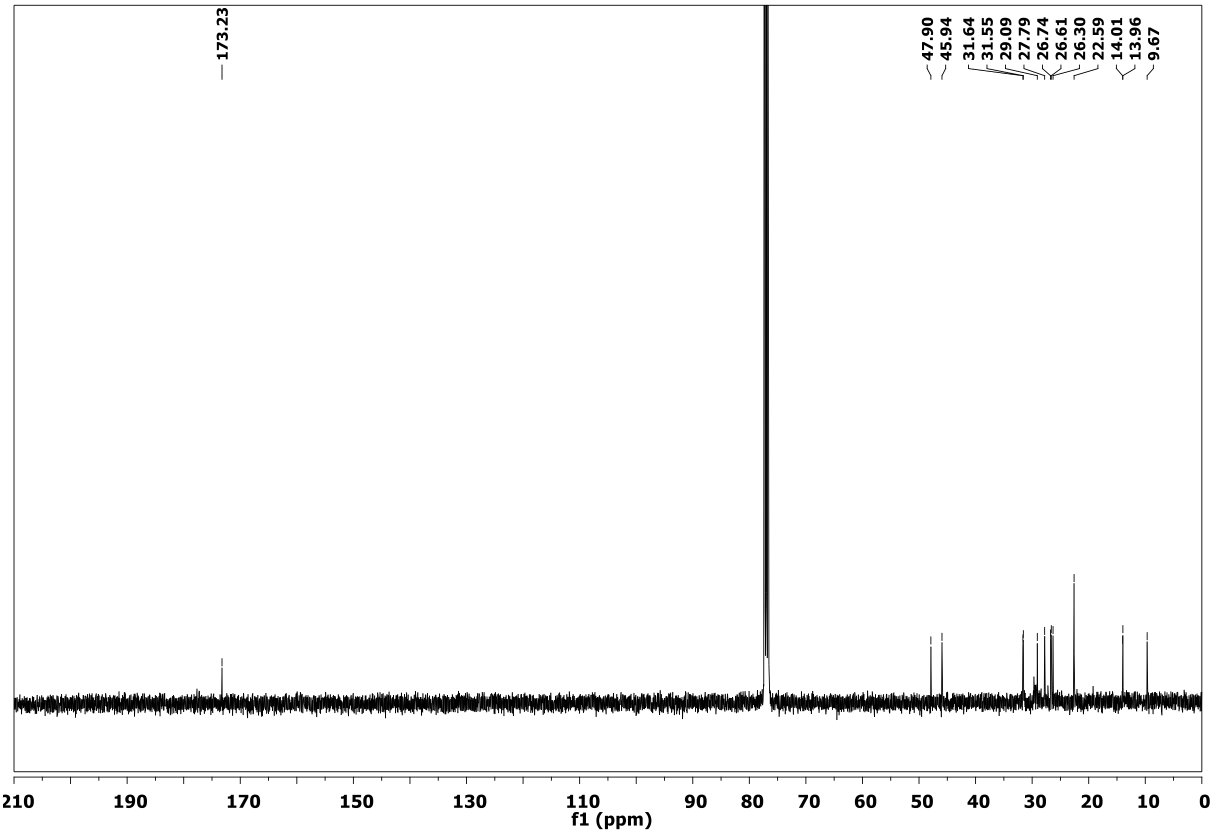

*Figure S22. ^{13}C{H} NMR of N,N-dihexylpropionamide **(5b)** ($CDCl_3$), 101 MHz.*

N,N-dioctylpropionamide ($C_{19}H_{39}NO$, 6b)

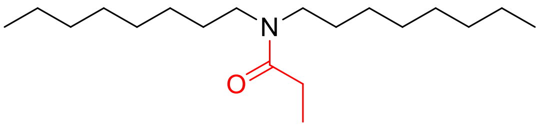

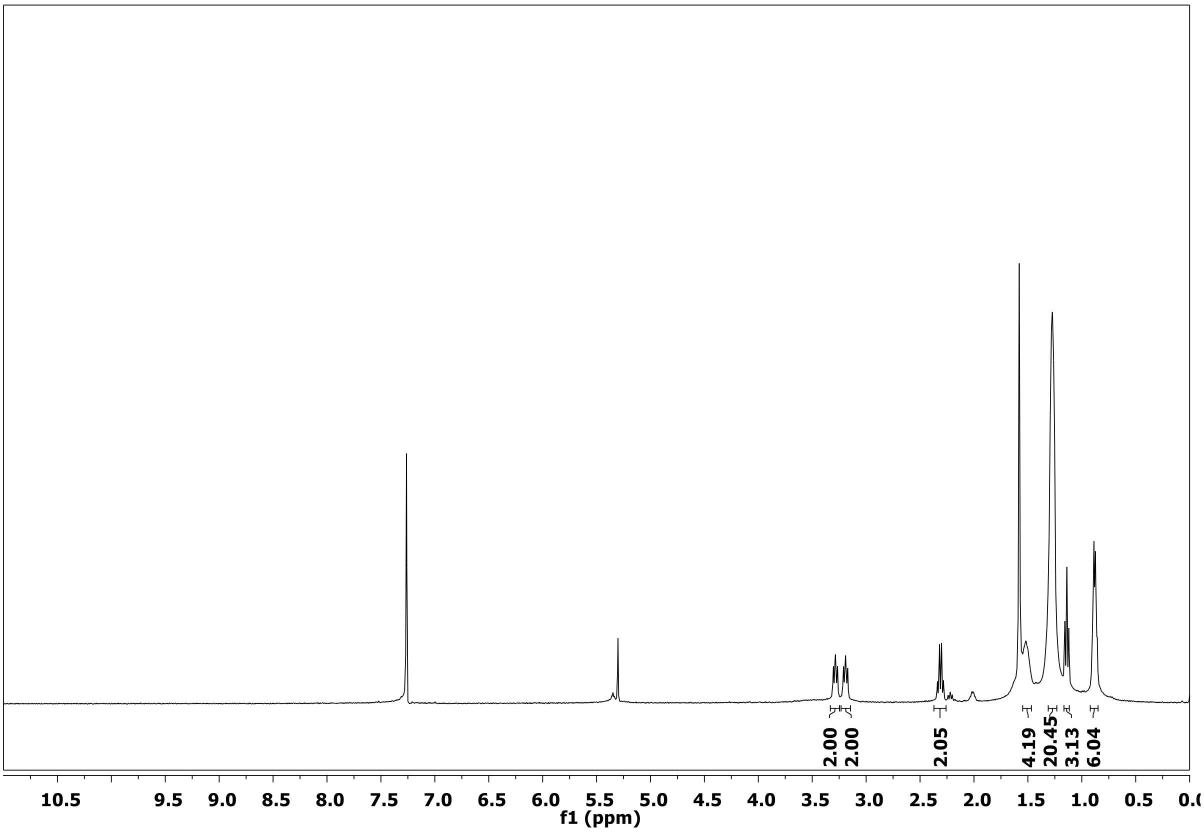

*Figure S23. ^{1}H NMR of N,N-dioctylpropionamide **(6b)** ($CDCl_3$), 400 MHz.*

1-(piperidin-1-yl)propan-1-one ($C_8H_{15}NO$, 8b)

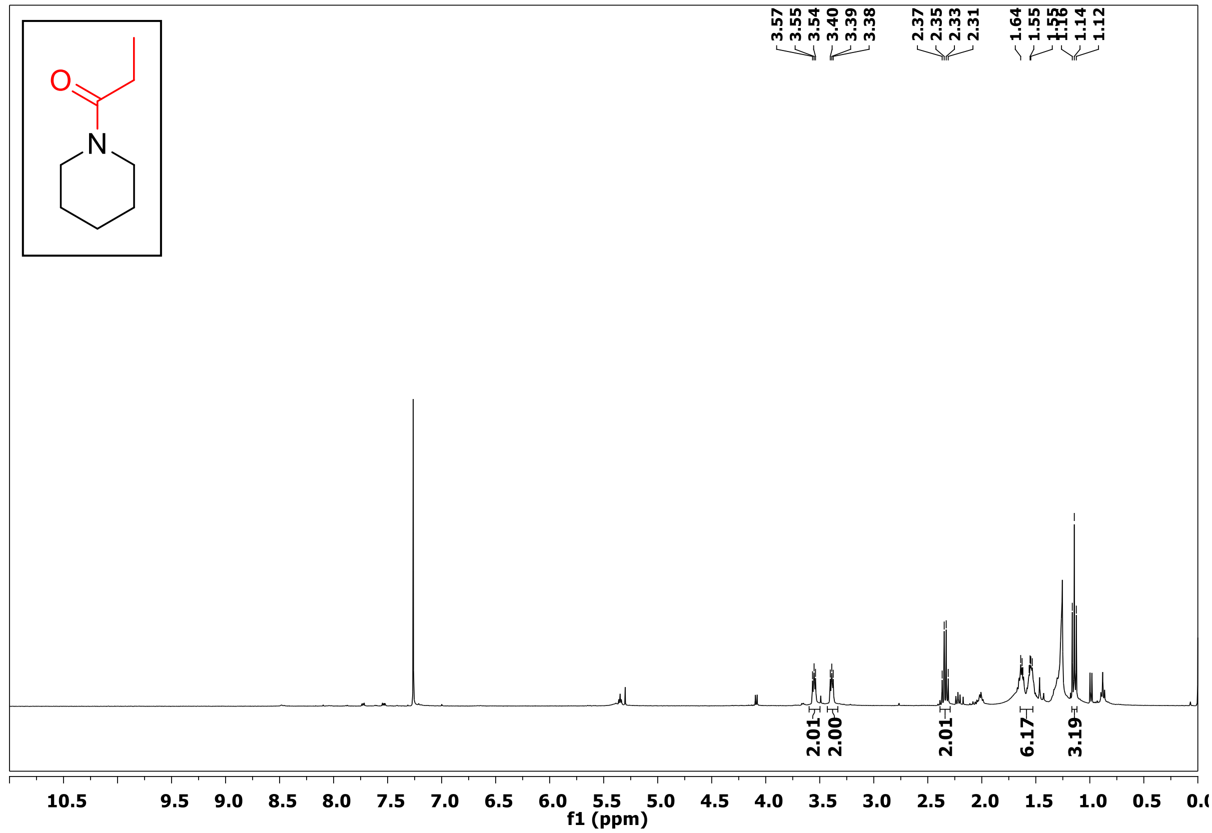

*Figure S24. ¹H NMR of 1-(piperidin-1-yl)propan-1-one **(8b)** (CDCl₃), 400 MHz.*

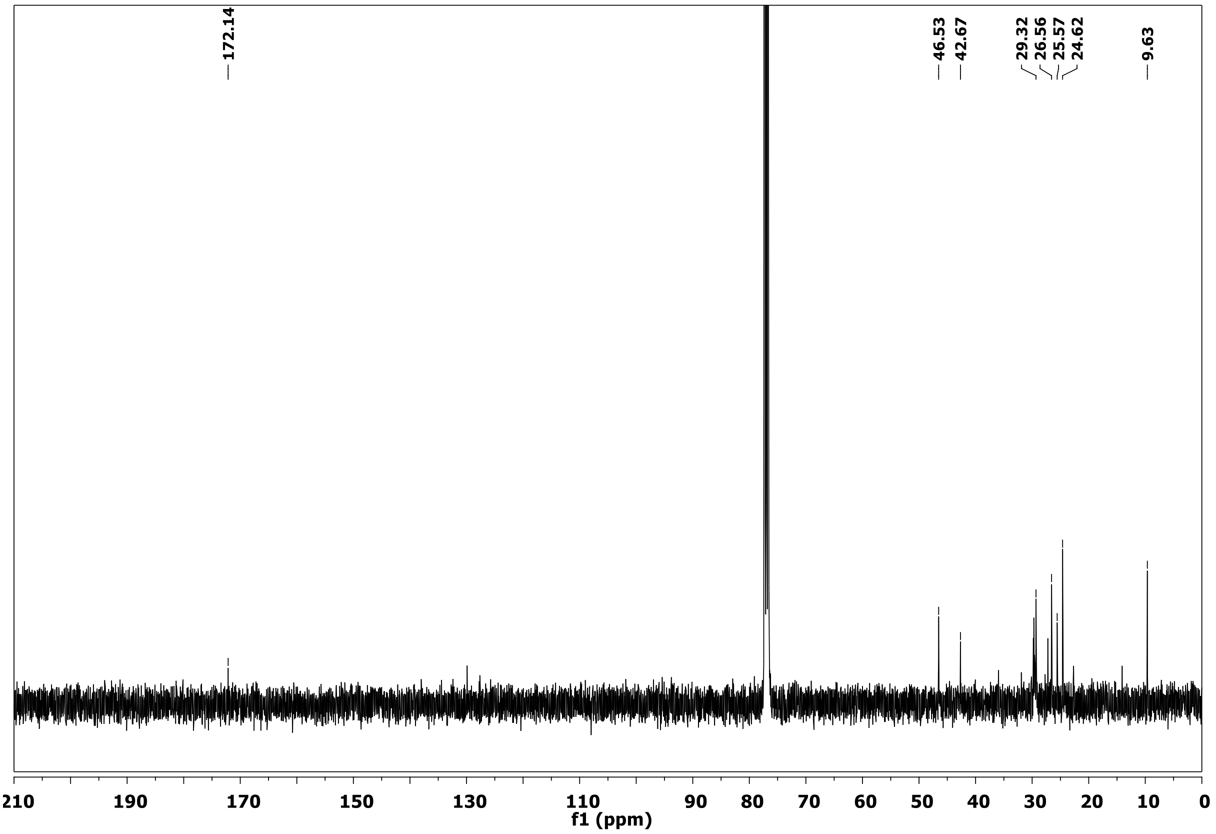

*Figure S25. ¹³C{H} NMR of 1-(piperidin-1-yl)propan-1-one **(8b)** (CDCl₃), 101 MHz.*

Dibutylcarbamothioic fluoride ($C_9H_{18}FNS$, 1c)

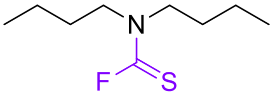

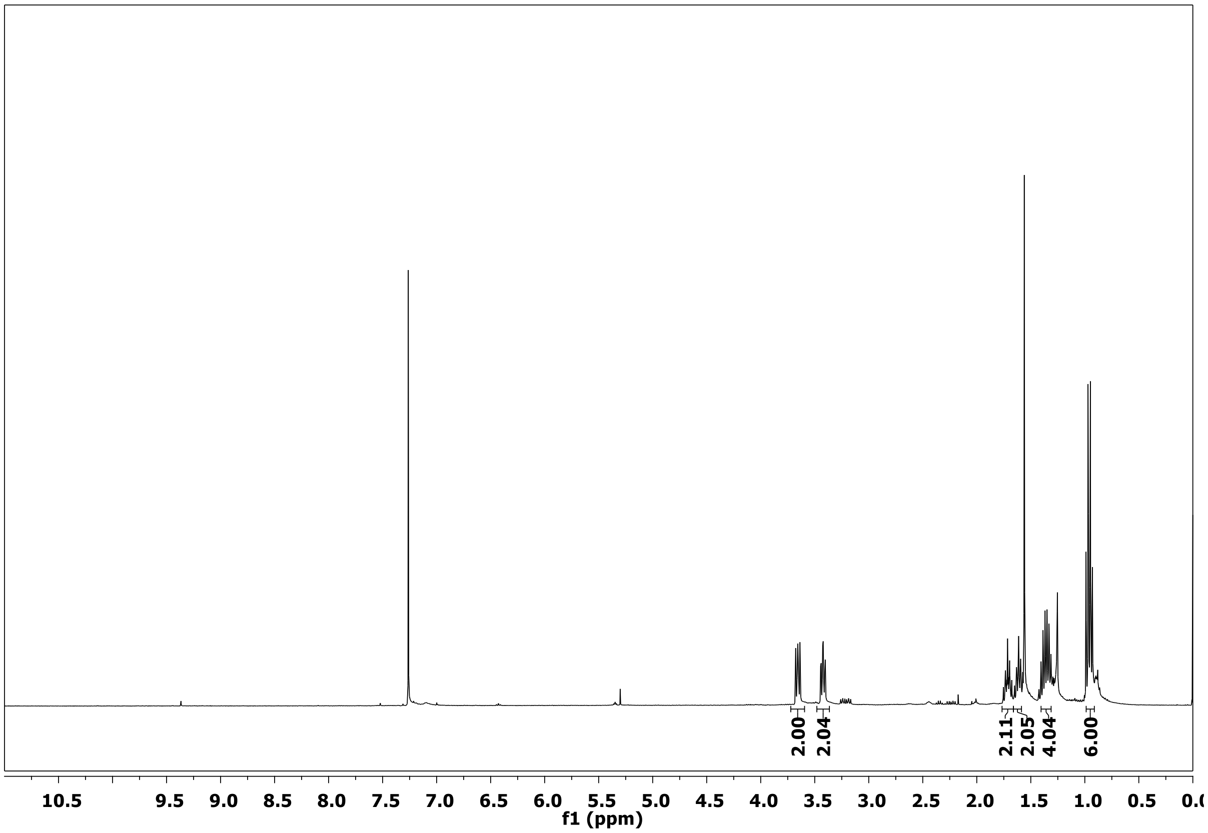

Figure S26. ^{1}H NMR of dibutylcarbamothioic fluoride ***(1c)*** *($CDCl_3$), 400 MHz.*

Diethylcarbamothioic fluoride ($C_5H_{10}FNS$, 2c)

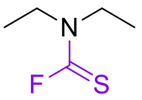

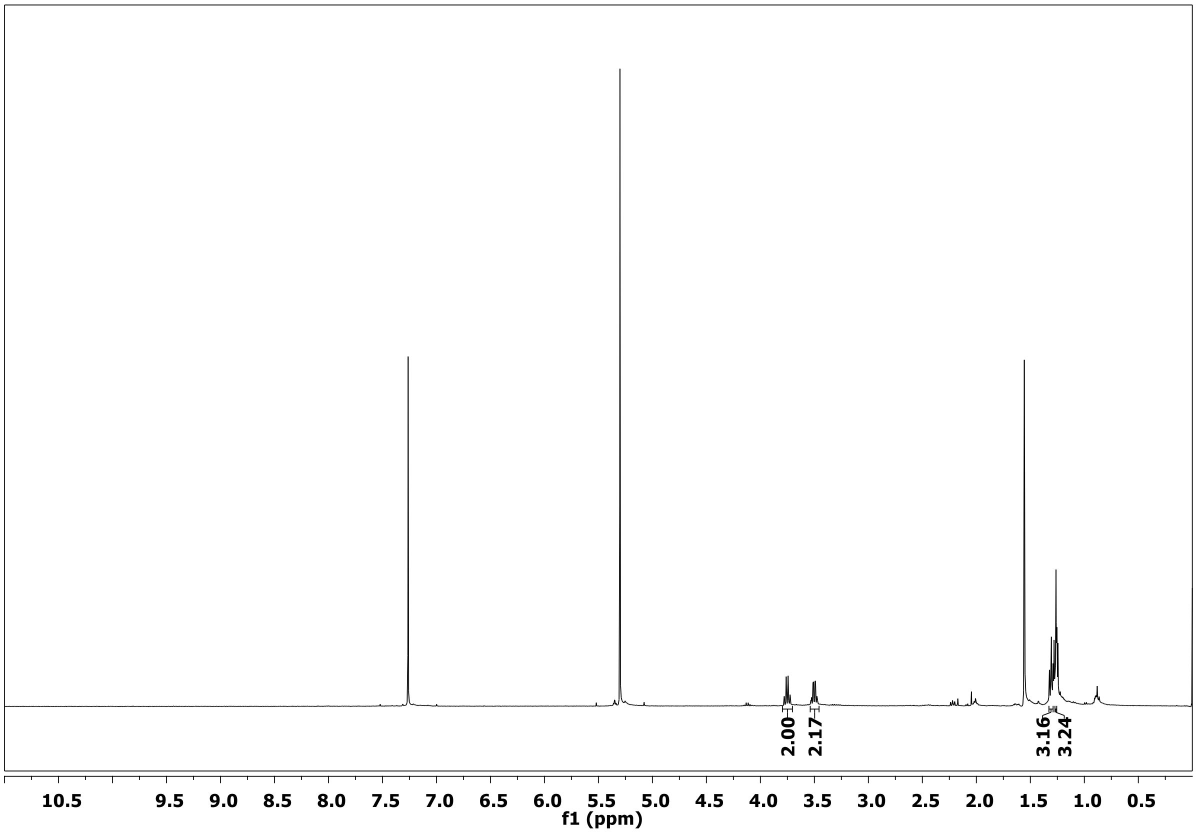

Figure S27. ^{1}*H NMR of diethylcarbamothioic fluoride* ***(2c)*** *($CDCl_3$), 400 MHz.*

Dipropylcarbamothioic fluoride ($C_7H_{14}FNS$, 3c)

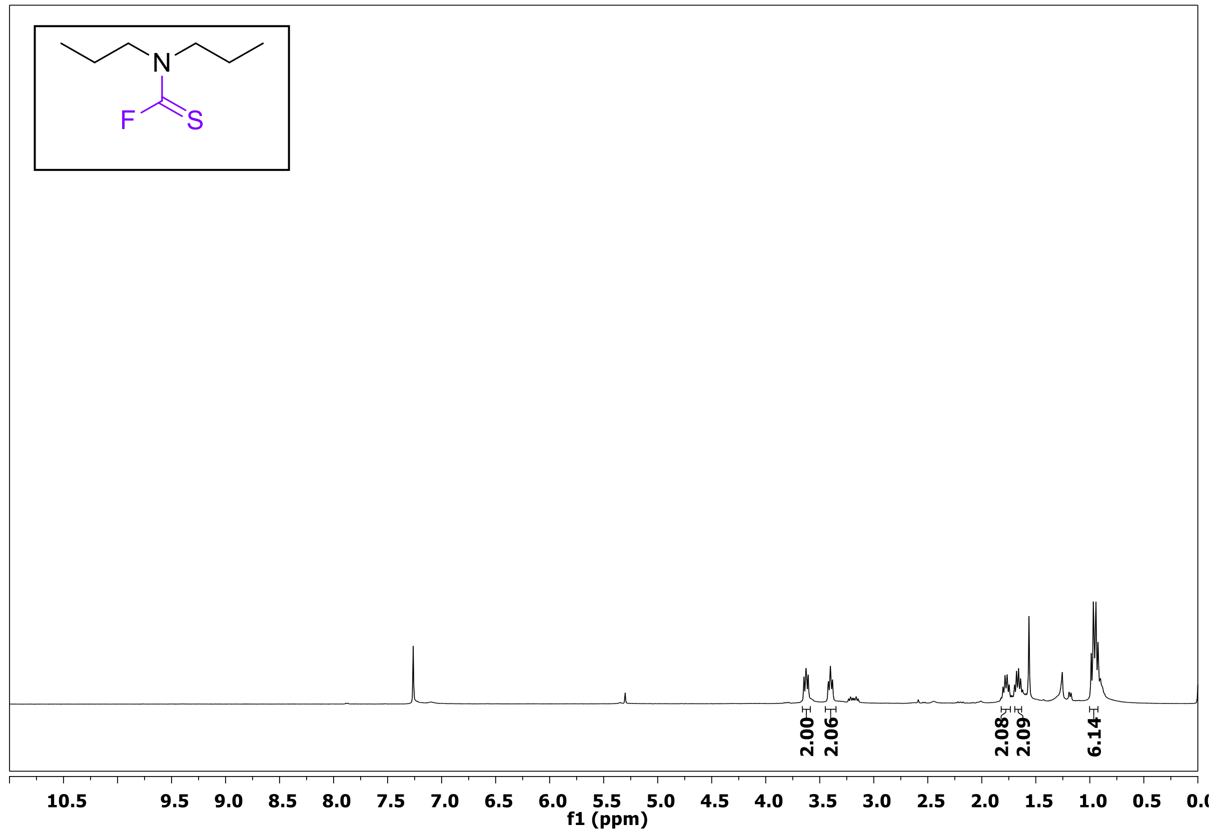

*Figure S28. ^{1}H NMR spectrum of dipropylcarbamothioic fluoride **(3c)** ($CDCl_3$), 400 MHz.*

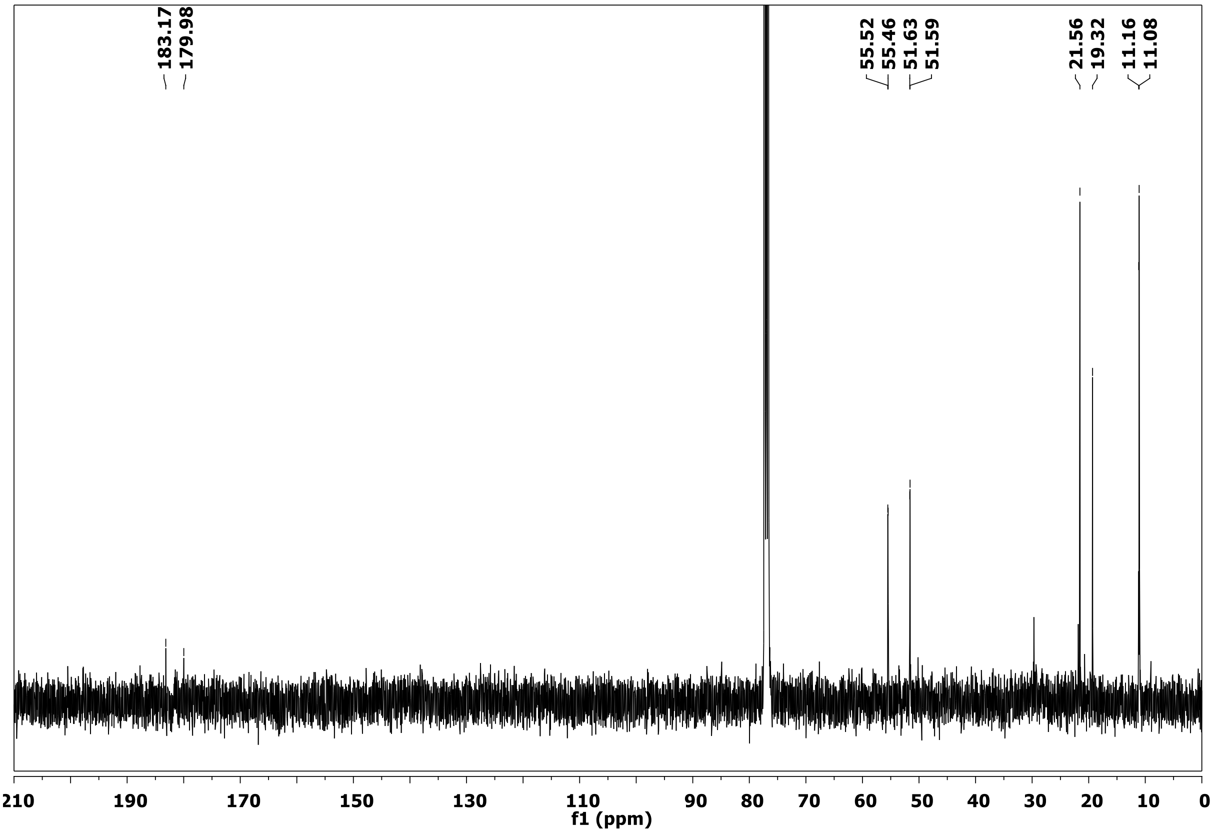

*Figure S29. $^{13}C\{H\}$ NMR spectrum of dipropylcarbamothioic fluoride **(3c)** ($CDCl_3$), 101 MHz.*

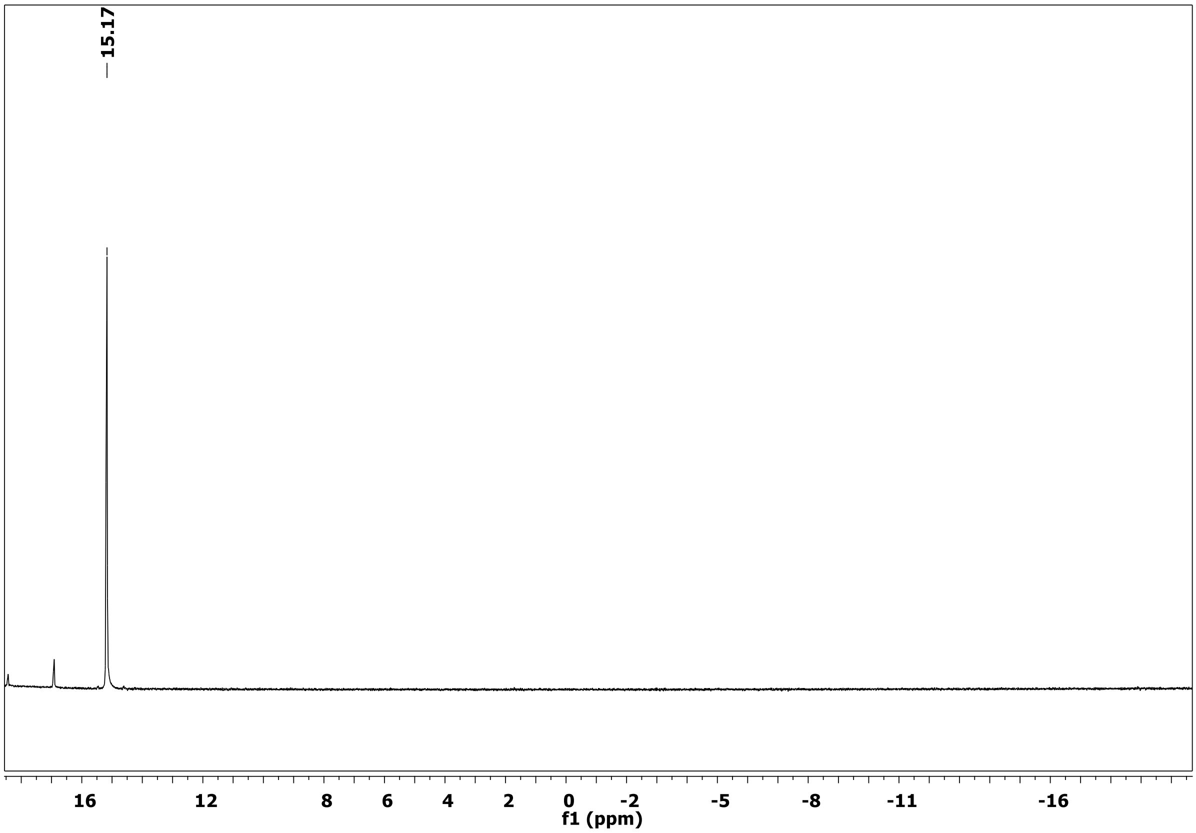

Figure S30. ^{19}*F NMR spectrum of dipropylcarbamothioic fluoride* ***(3c)*** *(*$CDCl_3$*), 376 MHz.*

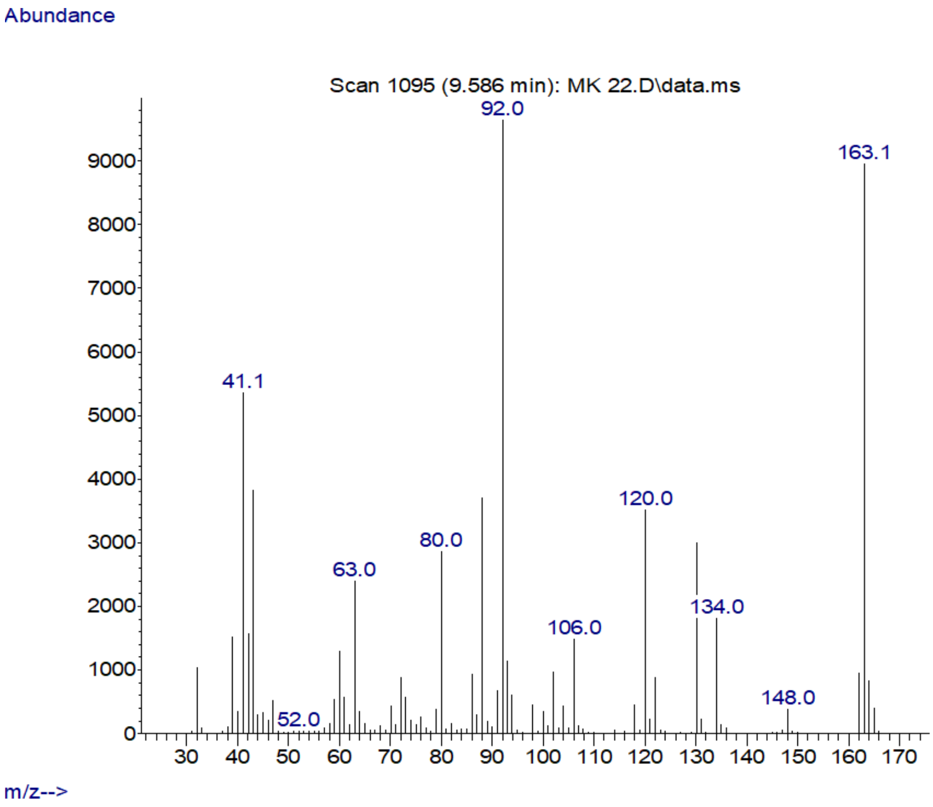

Figure S31. MS Spectrum of dipropylcarbamothioic fluoride ***(3c).***

Dipentylcarbamothioic fluoride ($C_{11}H_{22}FNS$, 4c)

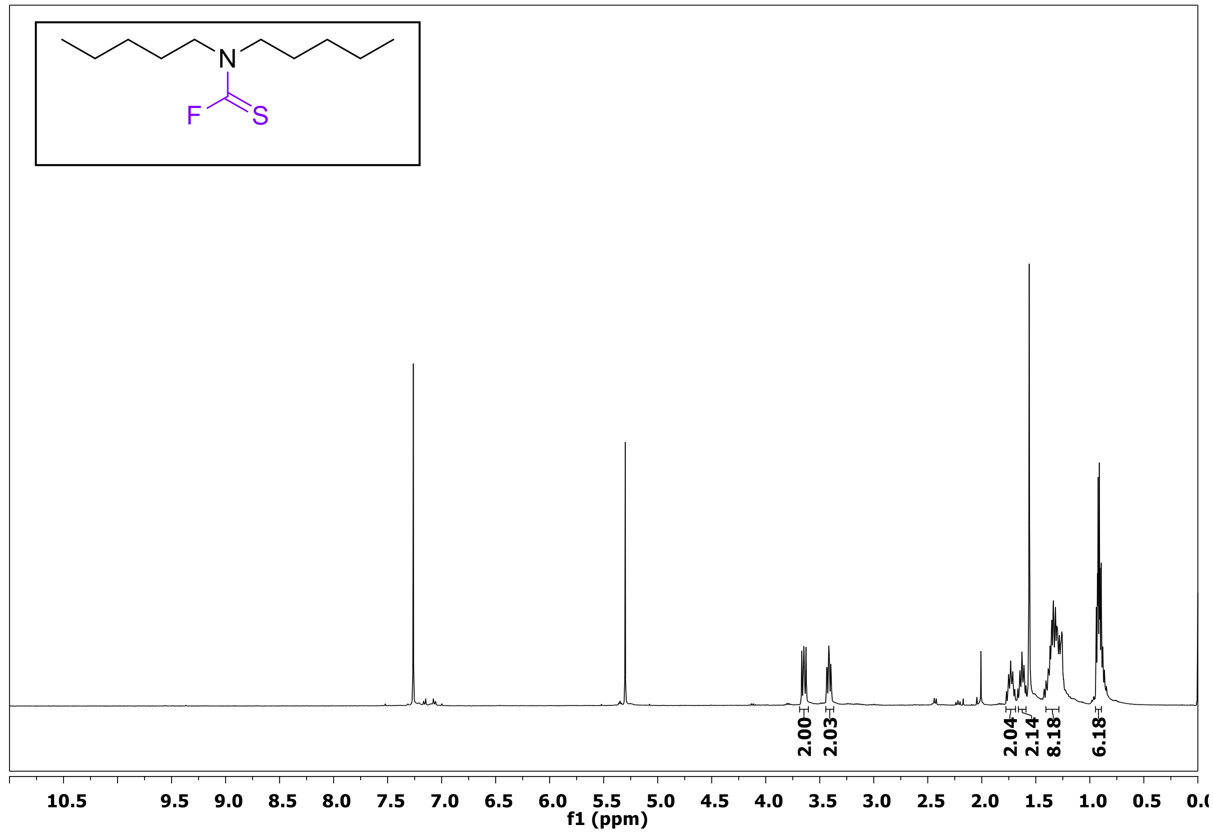

*Figure S32. ^{1}H NMR spectrum of dipentylcarbamothioic fluoride **(4c)** ($CDCl_3$), 400 MHz.*

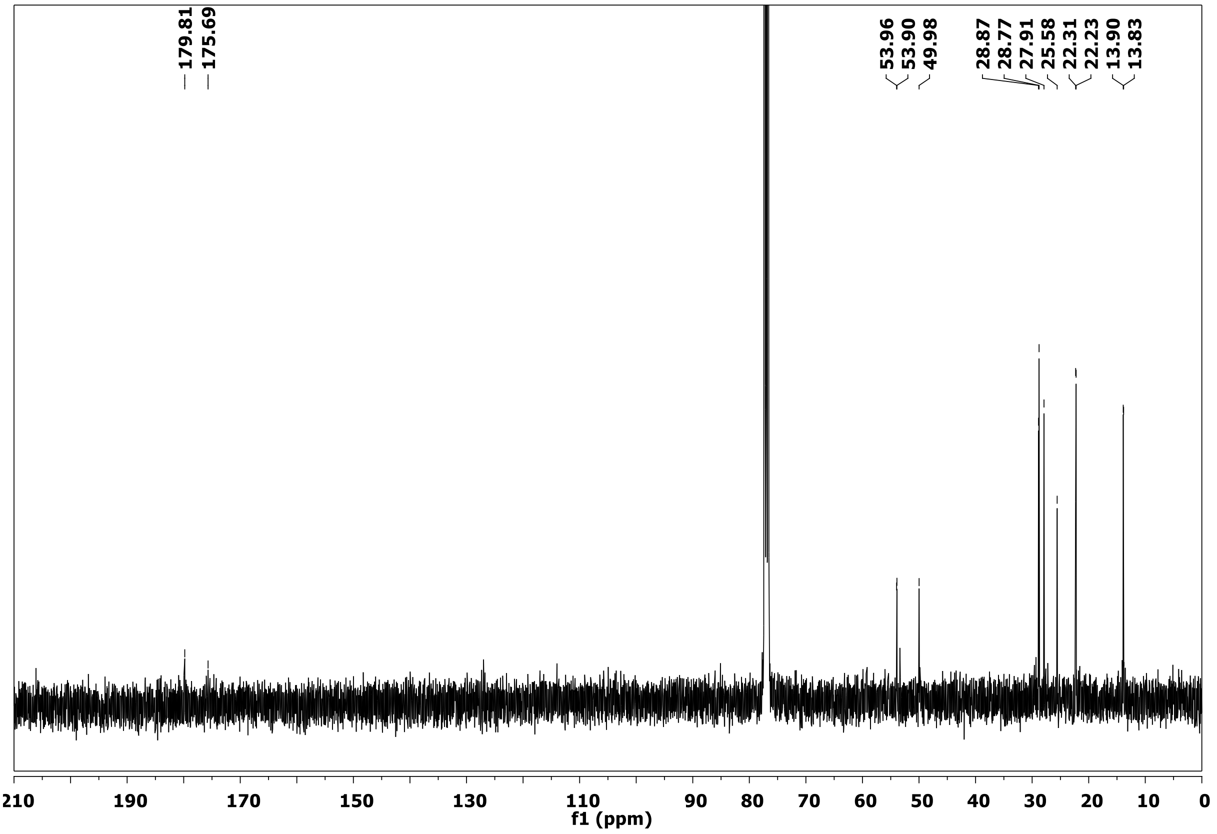

*Figure S33. ^{13}C{H} NMR spectrum of dipentylcarbamothioic fluoride **(4c)** ($CDCl_3$), 101 MHz.*

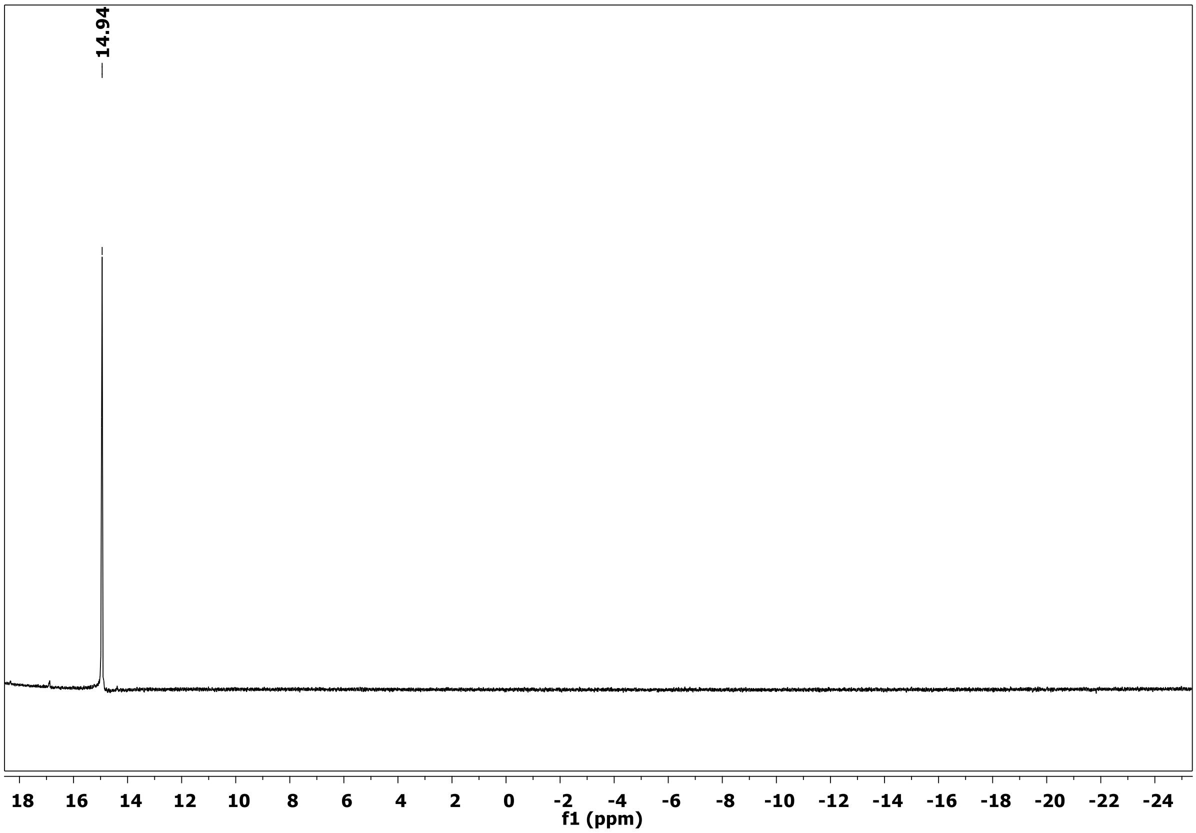

Figure S34. ^{19}F NMR spectrum of dipentylcarbamothioic fluoride ***(4c)*** *($CDCl_3$), 376 MHz.*

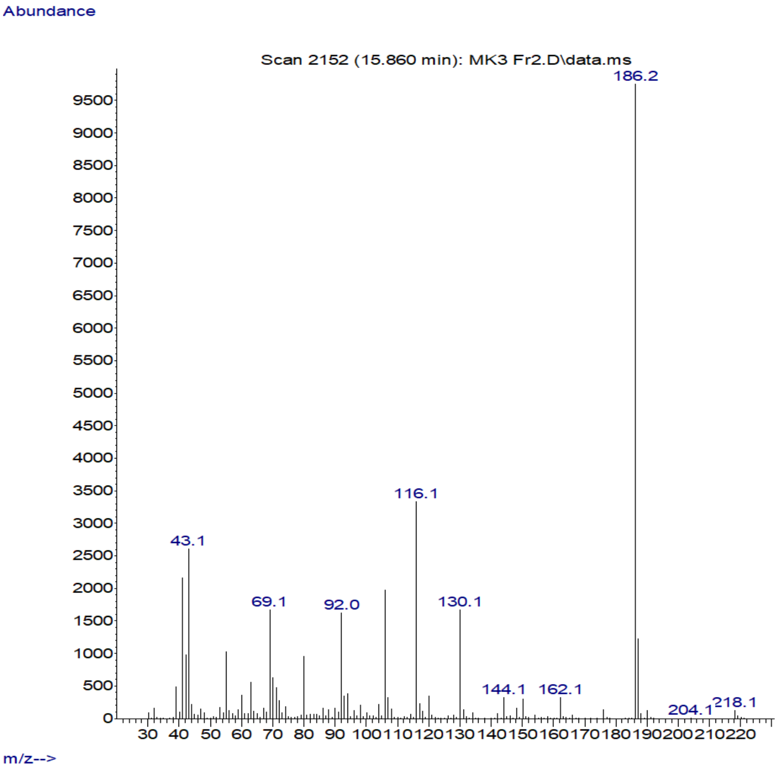

Figure S35. MS Spectrum of dipentylcarbamothioic fluoride ***(4c)***

Dihexylcarbamothioic fluoride ($C_{13}H_{26}FNS$, 5c)

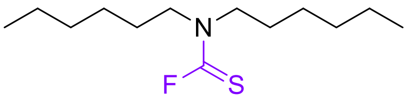

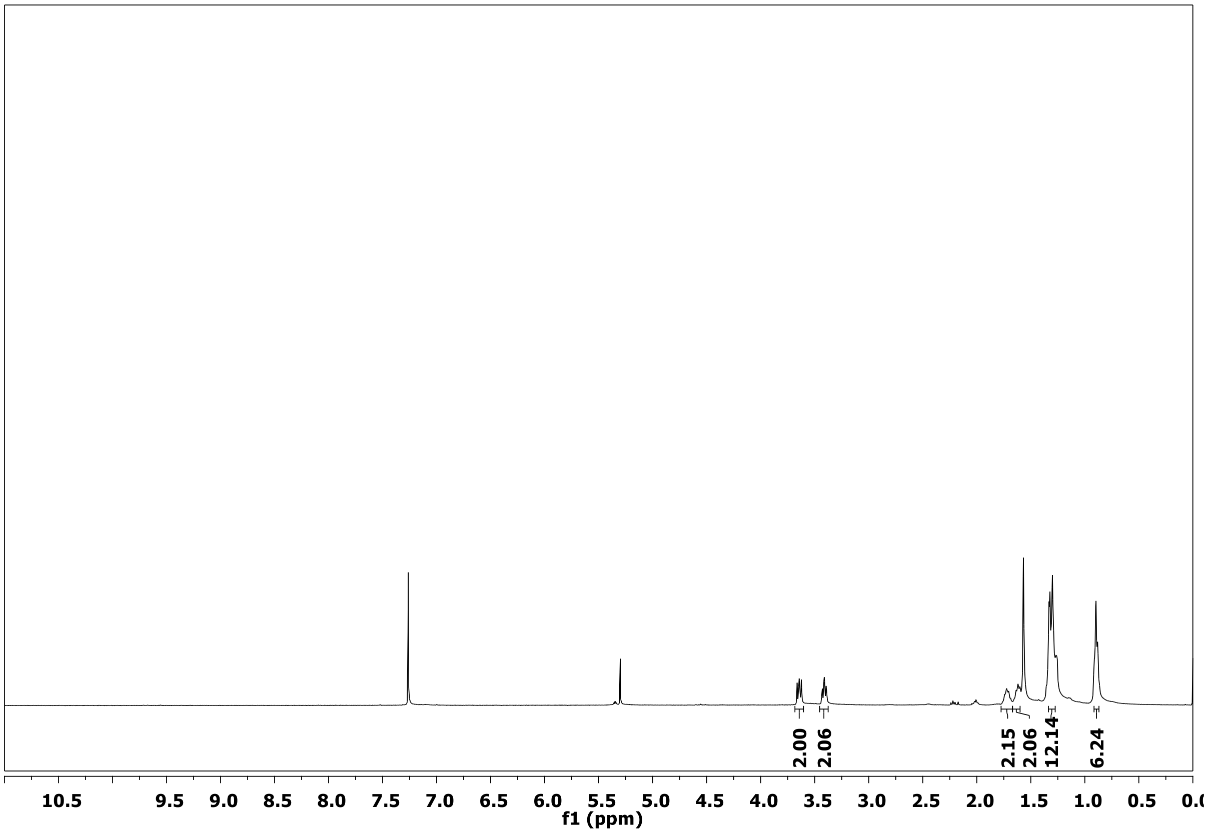

*Figure S36. ^{1}H NMR spectrum of dihexylcarbamothioic fluoride **(5c)** ($CDCl_3$), 400 MHz.*

Dioctylcarbamothioic fluoride ($C_{17}H_{34}F_3N$, 6c)

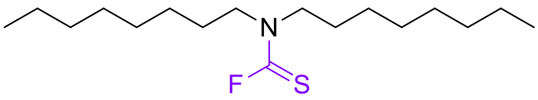

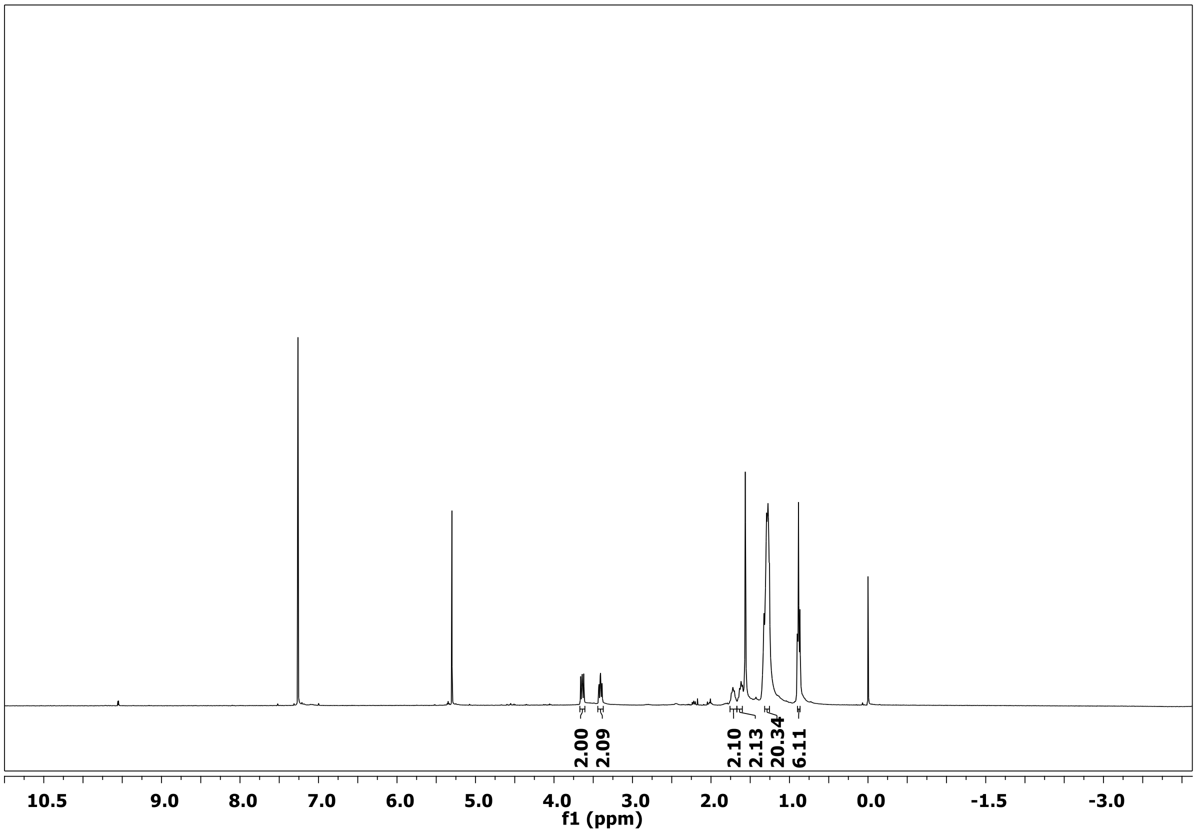

*Figure S37. ^{1}H NMR spectrum of dioctylcarbamothioic fluoride **(6c)** ($CDCl_3$), 400 MHz.*

Morpholine-4-carbothioyl fluoride (C_5H_8FNOS, 7c)

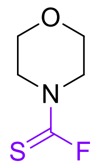

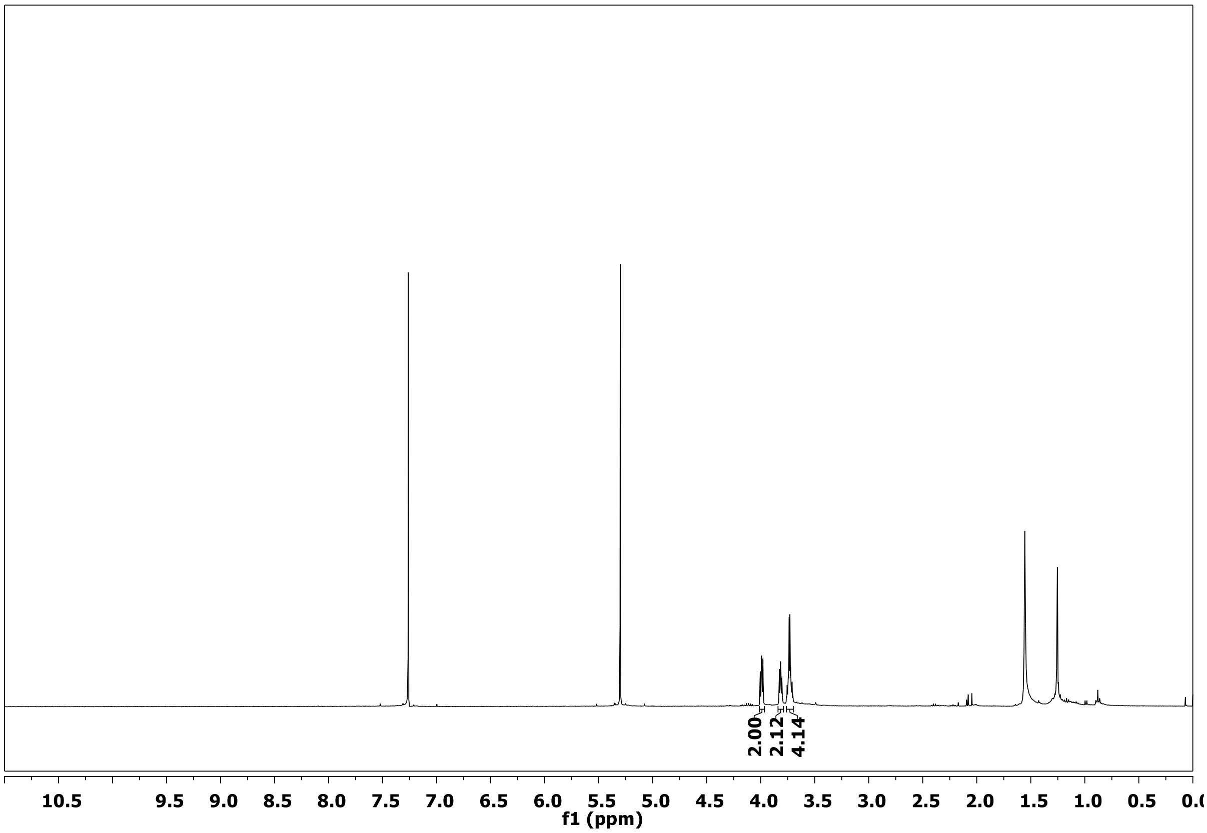

*Figure S38. ^1H NMR spectrum of morpholine-4-carbothioyl fluoride **(7c)** ($CDCl_3$), 400 MHz.*

Piperidine-1-carbothioyl fluoride ($C_6H_{10}FNS$, 8c)

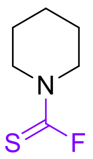

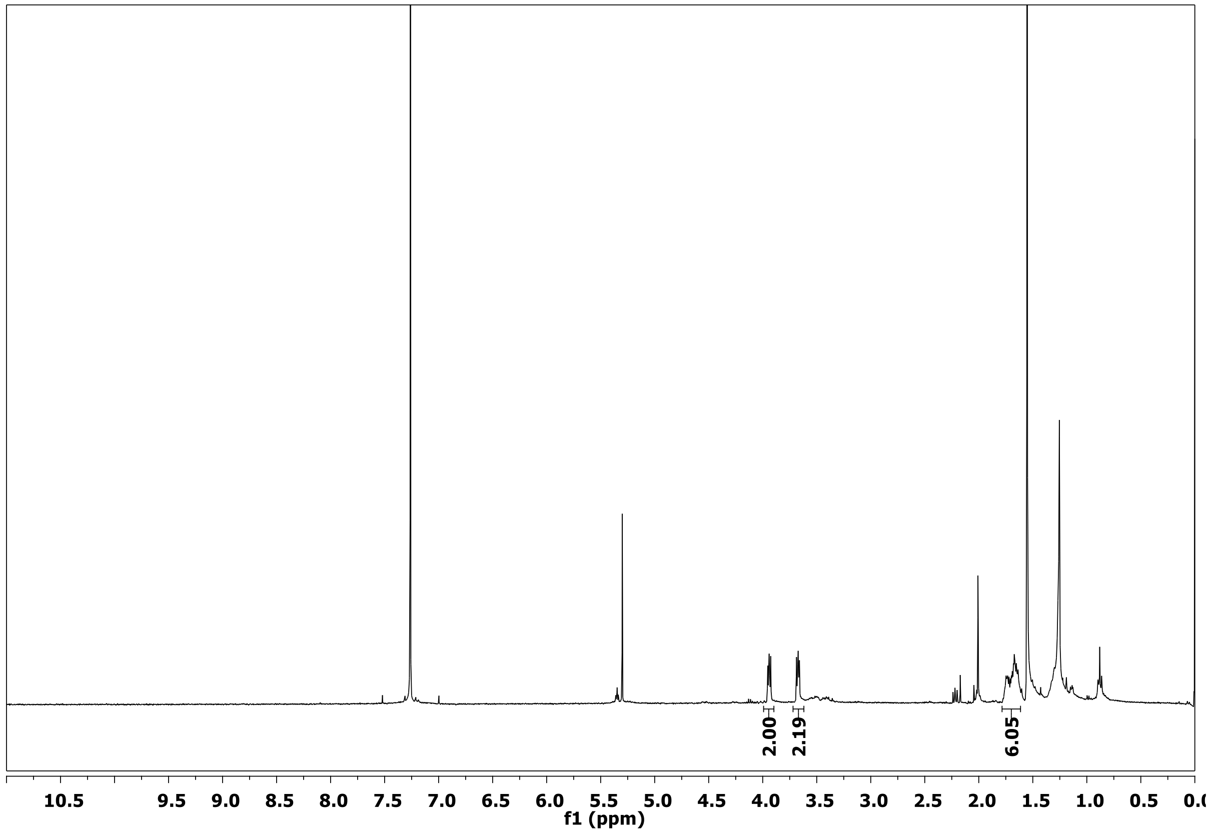

*Figure S39. ^1H NMR spectrum of piperidine-1-carbothioyl fluoride **(8c)** ($CDCl_3$), 400 MHz.*

(S)-*N,N*-dibutyl-2-(4-isobutylphenyl)propanamide ($C_{21}H_{35}NO$, 1d)

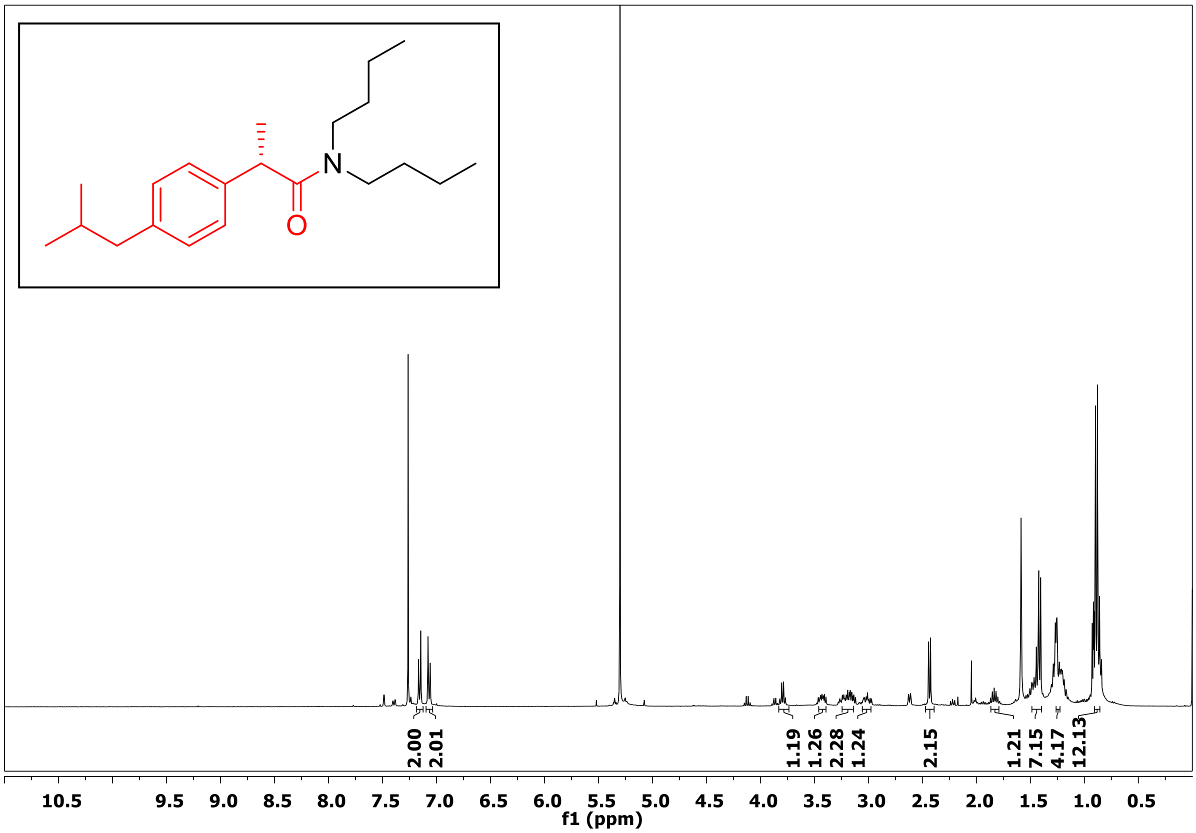

Figure S40. ^{1}H NMR spectrum of (S)-N,N-dibutyl-2-(4-isobutylphenyl)propanamide ***(1d)*** *(CDCl$_3$), 400 MHz.*

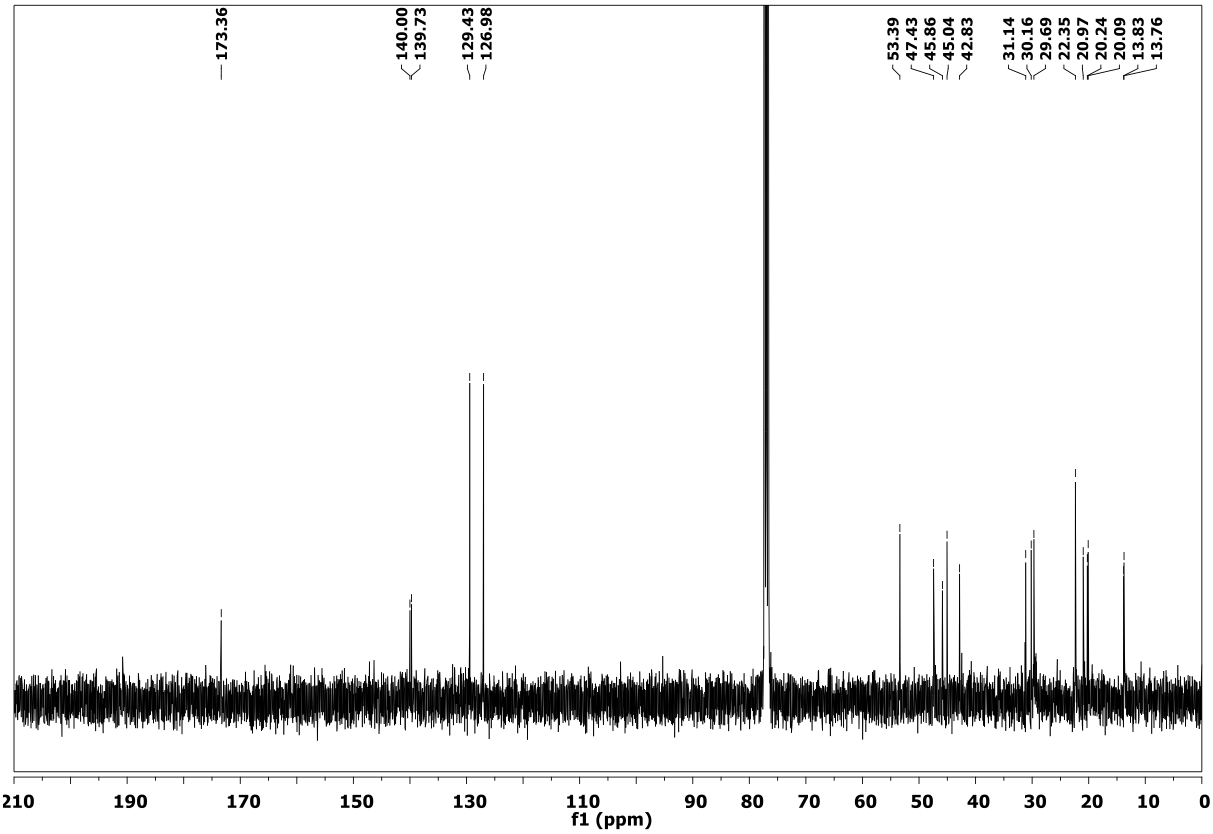

Figure S41. ^{13}C{H} NMR spectrum of (S)-N,N-dibutyl-2-(4-isobutylphenyl)propanamide ***(1d)*** *(CDCl$_3$), 101 MHz.*

Elemental Composition Report

Single Mass Analysis

Tolerance = 1000.0 PPM / DBE: min = -5.5, max = 1000.0
Element prediction: Off
Number of isotope peaks used for i-FIT = 9

Monoisotopic Mass, Even Electron Ions
1 formula(e) evaluated with 1 results within limits (all results (up to 1000) for each mass)
Elements Used:
C: 21-21 H: 35-36 N: 1-1 O: 1-1

Ozgur Yilmaz
43337_20240513_04-02 13 (0.518) Cm (8:14)
1: TOF MS ES+
7.98e+004

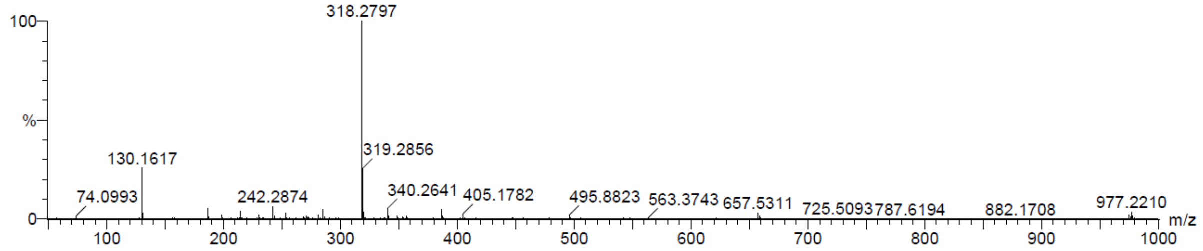

Minimum:				-5.5			
Maximum:		1000.0	1000.0	1000.0			
Mass	Calc. Mass	mDa	PPM	DBE	i-FIT	i-FIT (Norm)	Formula
318.2797	318.2797	0.0	0.0	4.5	1069.1	0.0	C21 H36 N O

Figure S42. HRMS result of (S)-N,N-dibutyl-2-(4-isobutylphenyl)propanamide ***(1d).***

(S)-*N,N*-diethyl-2-(4-isobutylphenyl)propanamide ($C_{17}H_{27}NO$, 2d)

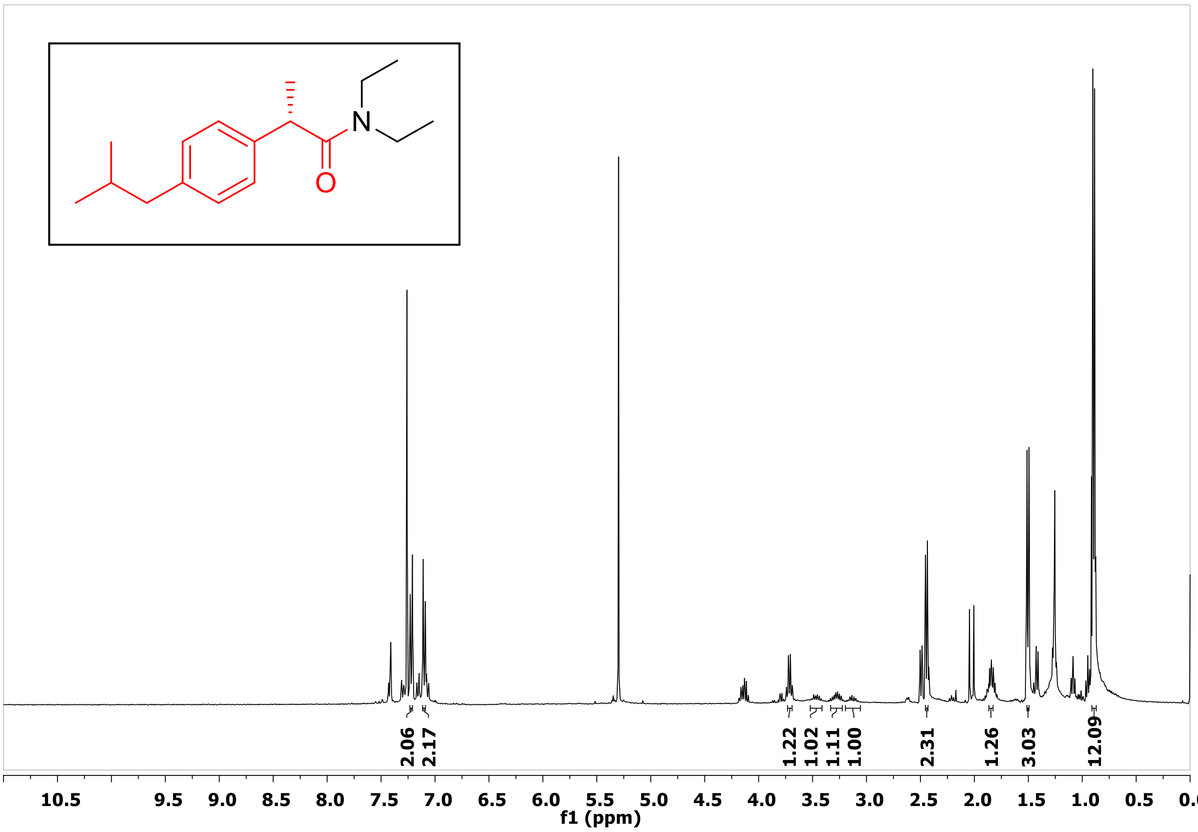

*Figure S43. ^{1}H NMR spectrum of (S)-N,N-diethyl-2-(4-isobutylphenyl)propanamide **(2d)** (CDCl₃), 400 MHz.*

(S)-2-(4-isobutylphenyl)-*N,N*-dipropylpropanamide ($C_{19}H_{31}NO$, 3d)

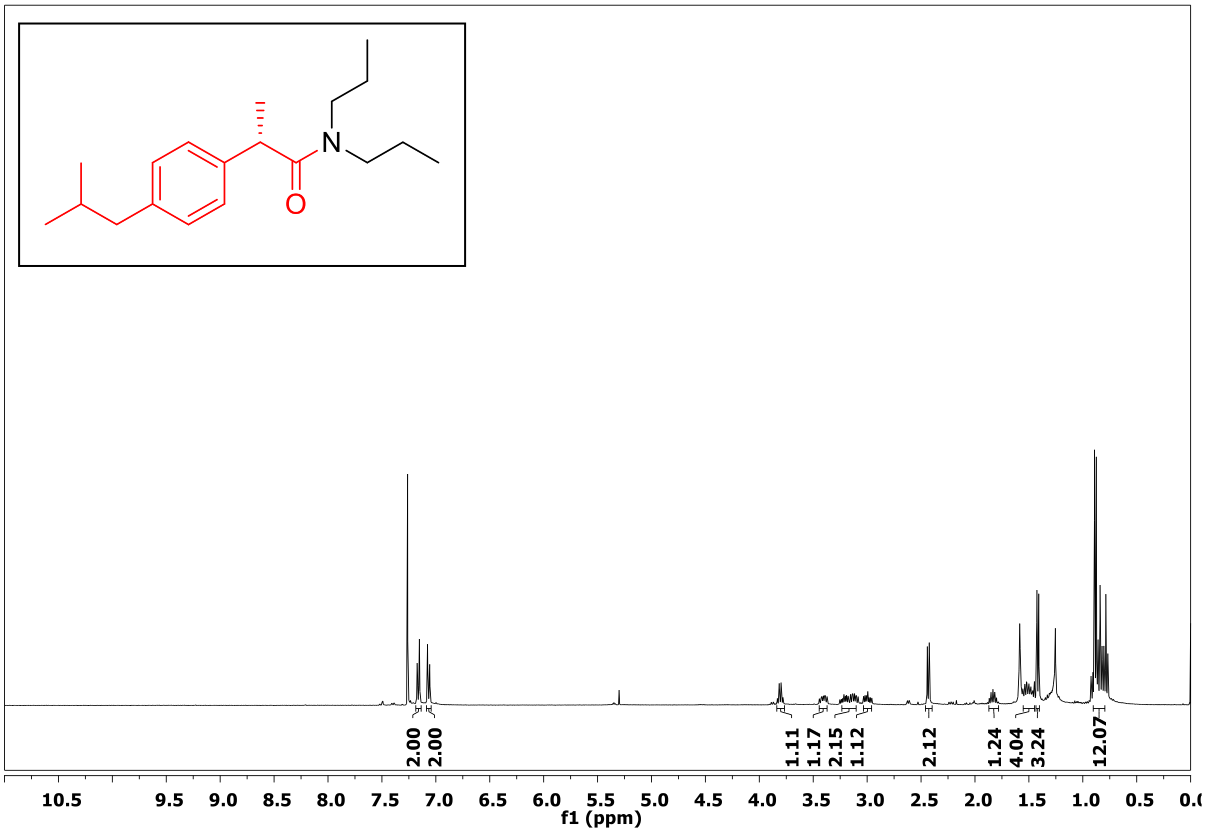

*Figure S44. ^{1}H NMR spectrum of (S)-2-(4-isobutylphenyl)-N,N-dipropylpropanamide **(3d)** ($CDCl_3$), 400 MHz.*

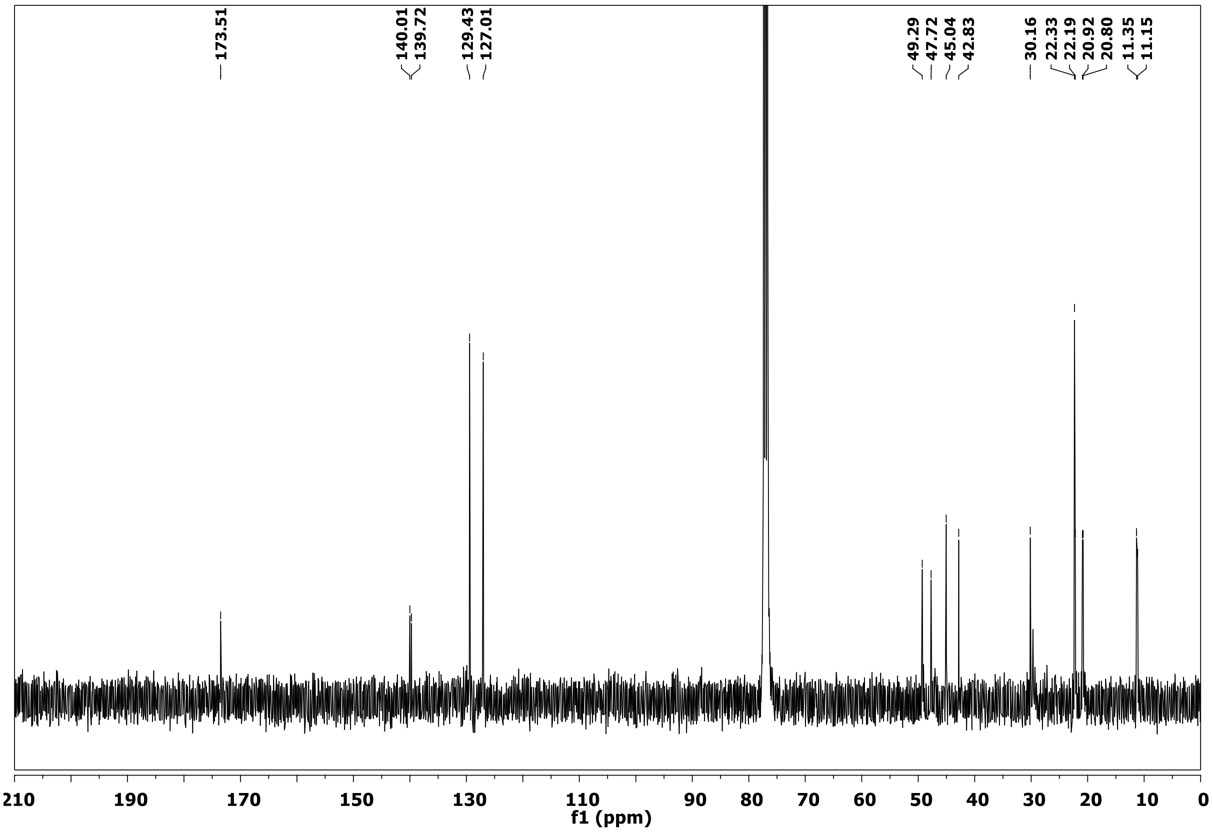

*Figure S45. $^{13}C\{H\}$ NMR spectrum of (S)-2-(4-isobutylphenyl)-N,N-dipropylpropanamide **(3d)** ($CDCl_3$), 101 MHz.*

Elemental Composition Report

Single Mass Analysis

Tolerance = 1000.0 PPM / DBE: min = -5.5, max = 1000.0
Element prediction: Off
Number of isotope peaks used for i-FIT = 9

Monoisotopic Mass, Even Electron Ions
1 formula(e) evaluated with 1 results within limits (all results (up to 1000) for each mass)
Elements Used:
C: 19-19 H: 31-32 N: 1-1 O: 1-1
Ozgur Yilmaz
43337_20240513_03-06 18 (0.707) Cm (13:20)

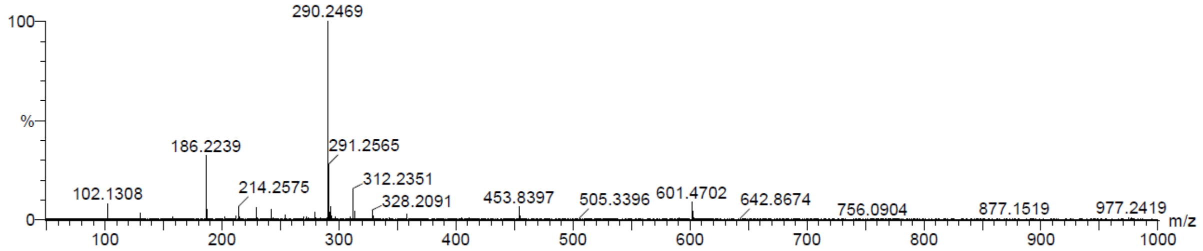

Minimum:				-5.5			
Maximum:		1000.0	1000.0	1000.0			
Mass	Calc. Mass	mDa	PPM	DBE	i-FIT	i-FIT (Norm)	Formula
290.2469	290.2484	-1.5	-5.2	4.5	554.9	0.0	C19 H32 N O

Figure S46. HRMS result of (S)-2-(4-isobutylphenyl)-N,N-dipropylpropanamide ***(3d).***

(S)-2-(4-isobutylphenyl)-*N,N*-dipentylpropanamide ($C_{23}H_{39}NO$, 4d)

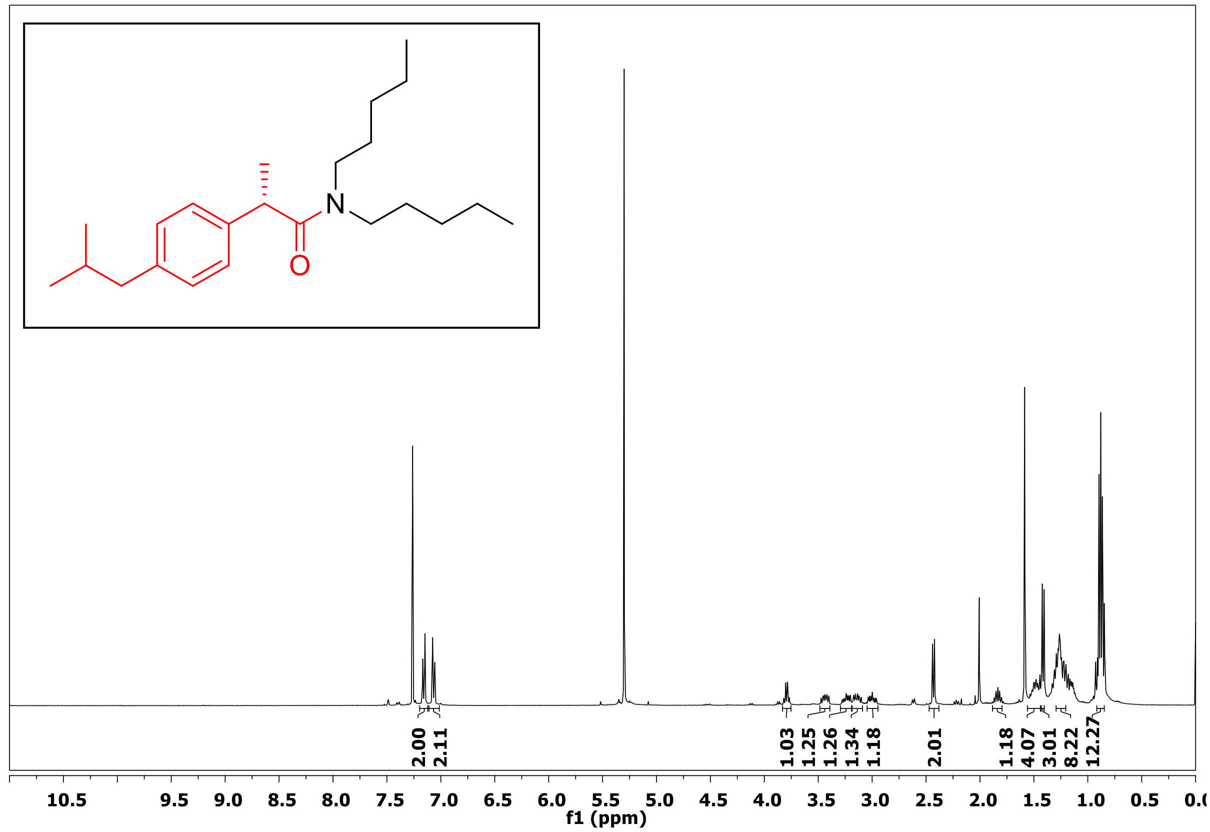

Figure S47. ¹H NMR spectrum of (S)-2-(4-isobutylphenyl)-N,N-dipentylpropanamide ***(4d)*** *($CDCl_3$), 400 MHz.*

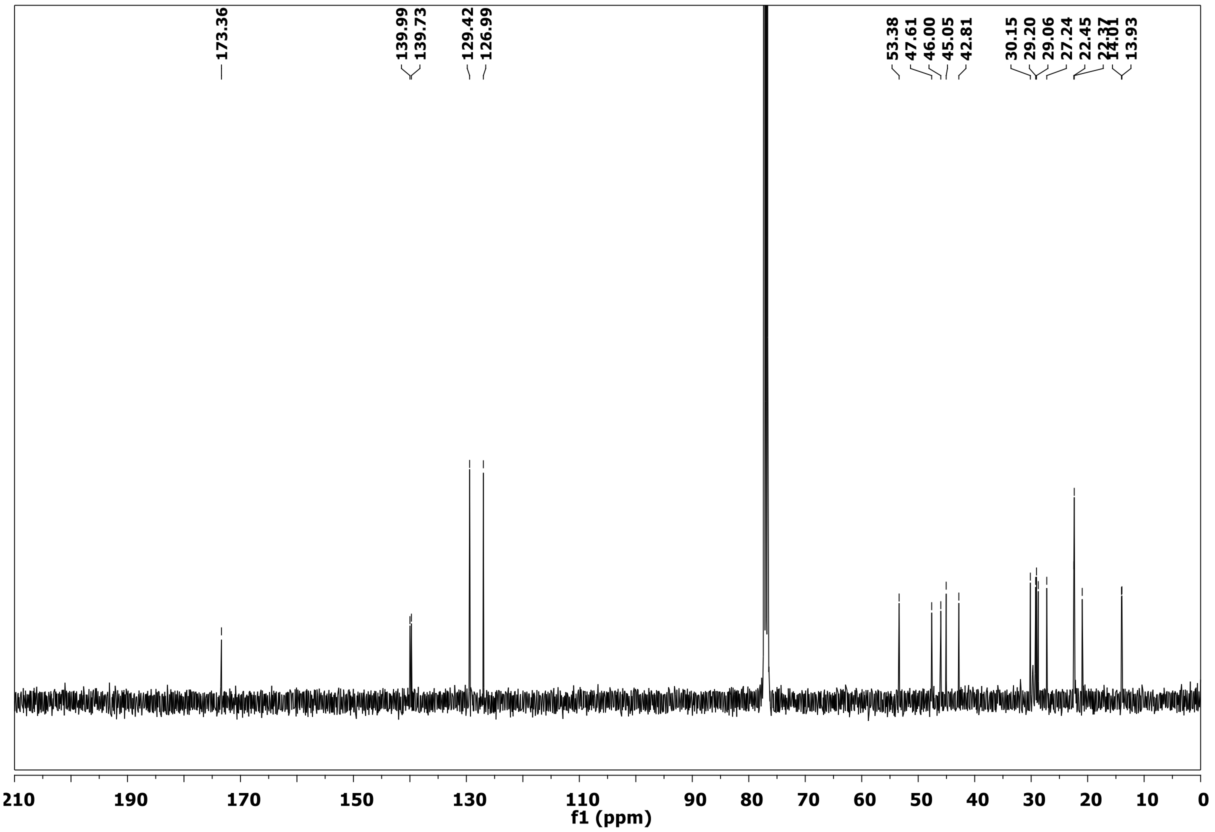

Figure S48. ¹³C{H} NMR spectrum of (S)-2-(4-isobutylphenyl)-N,N-dipentylpropanamide ***(4d)*** *($CDCl_3$), 101 MHz.*

Elemental Composition Report

Single Mass Analysis

Tolerance = 1000.0 PPM / DBE: min = -5.5, max = 1000.0
Element prediction: Off
Number of isotope peaks used for i-FIT = 9

Monoisotopic Mass, Even Electron Ions
1 formula(e) evaluated with 1 results within limits (all results (up to 1000) for each mass)
Elements Used:
C: 23-23 H: 39-40 N: 1-1 O: 1-1
Ozgur Yilmaz
43337_20240513_05-03 17 (0.673) Cm (10:19)

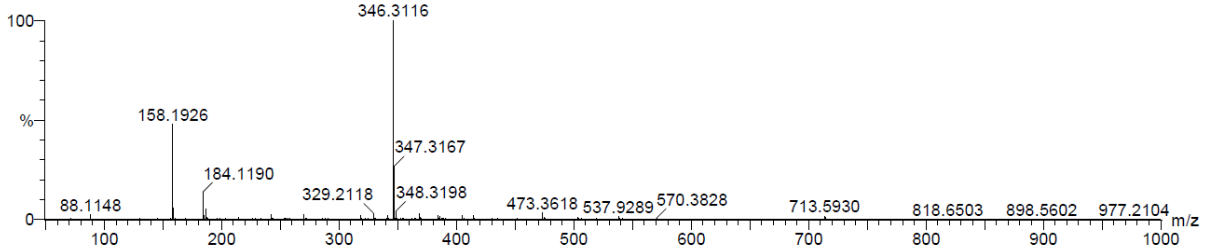

Minimum:				-5.5			
Maximum:		1000.0	1000.0	1000.0			
Mass	Calc. Mass	mDa	PPM	DBE	i-FIT	i-FIT (Norm)	Formula
346.3116	346.3110	0.6	1.7	4.5	1104.7	0.0	C23 H40 N O

Figure S49. HRMS result of (S)-2-(4-isobutylphenyl)-N,N-dipentylpropanamide ***(4d).***

(S)-*N,N*-dihexyl-2-(4-isobutylphenyl)propanamide ($C_{25}H_{43}NO$, 5d)

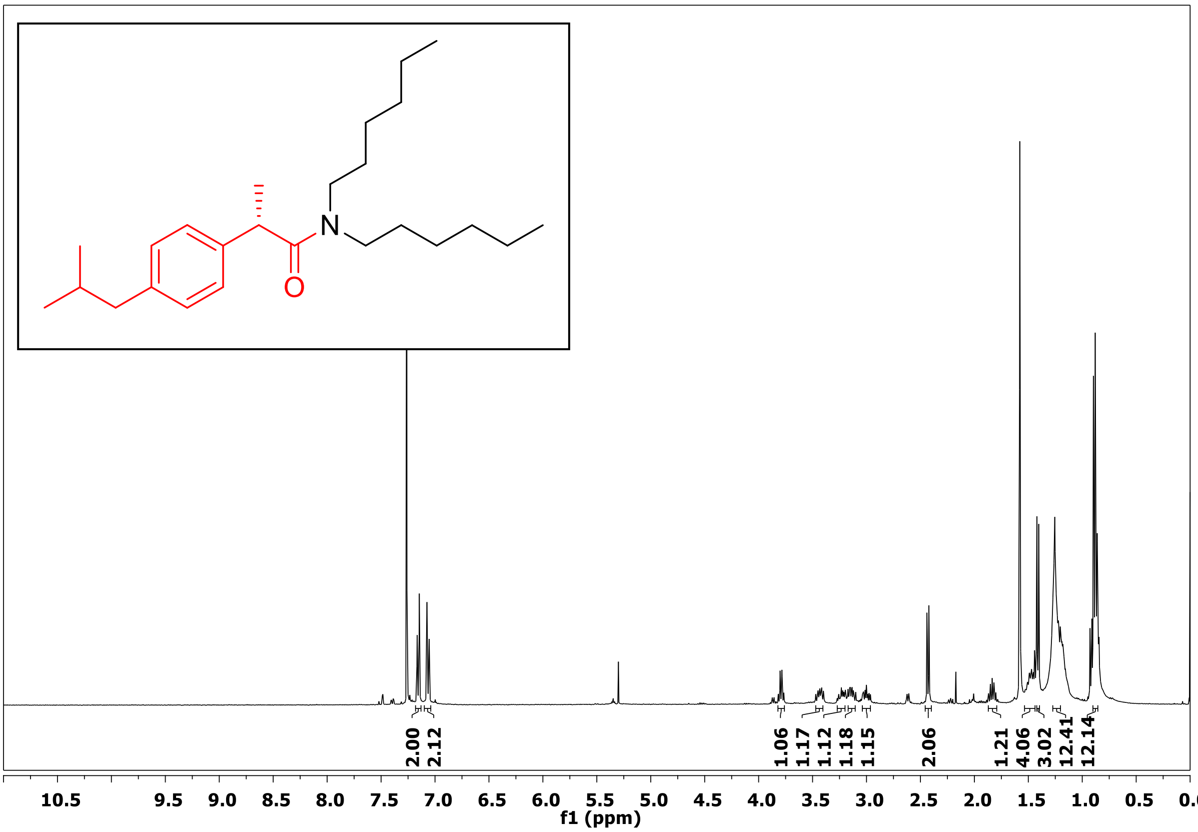

*Figure S50. ^{1}H NMR spectrum of (S)-N,N-dihexyl-2-(4-isobutylphenyl)propanamide **(5d)** (CDCl$_3$), 400 MHz.*

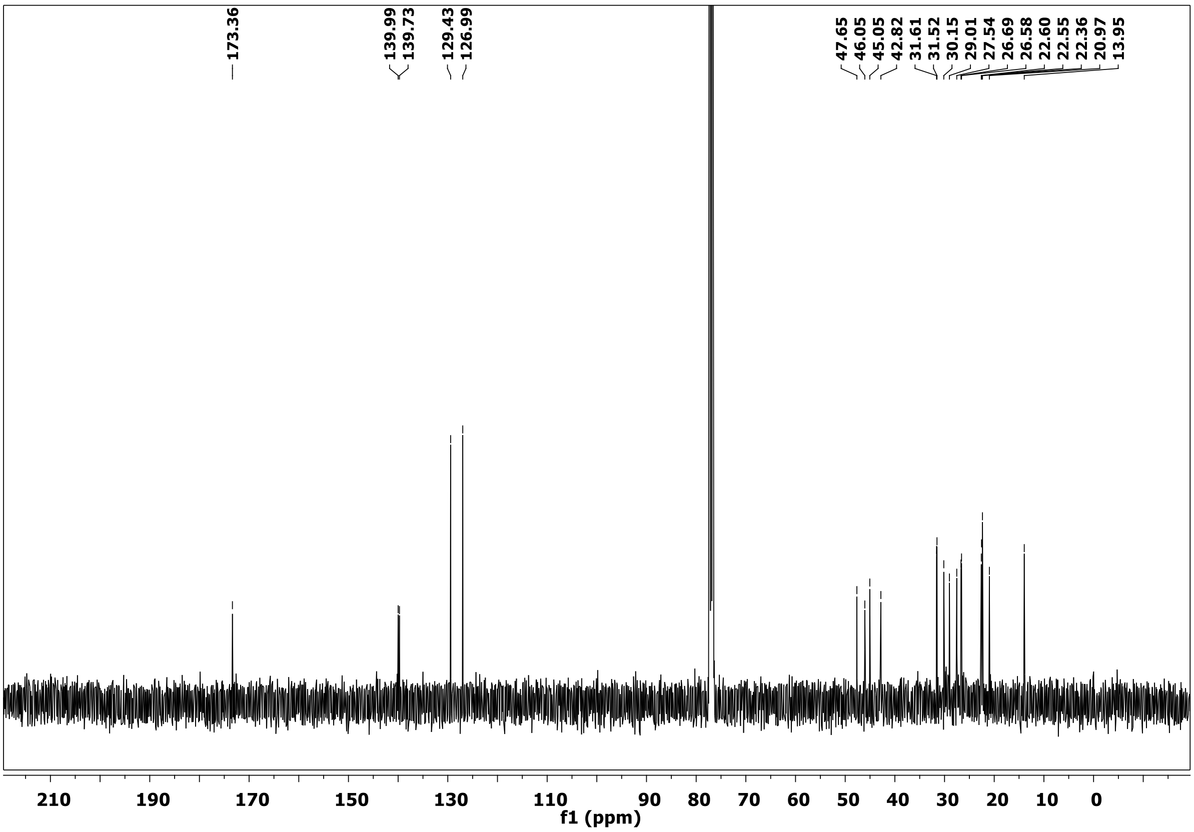

*Figure S51. ^{13}C{H} NMR spectrum of (S)-N,N-dihexyl-2-(4-isobutylphenyl)propanamide **(5d)** (CDCl$_3$), 101 MHz.*

Single Mass Analysis
Tolerance = 1000.0 PPM / DBE: min = -5.5, max = 1000.0
Element prediction: Off
Number of isotope peaks used for i-FIT = 9

Monoisotopic Mass, Odd and Even Electron Ions
1 formula(e) evaluated with 1 results within limits (all results (up to 1000) for each mass)
Elements Used:
C: 25-25 H: 43-44 N: 1-1 O: 1-1

Ozgur Yilmaz
43337_20240513_06-01 4 (0.172) Cm (1:10)

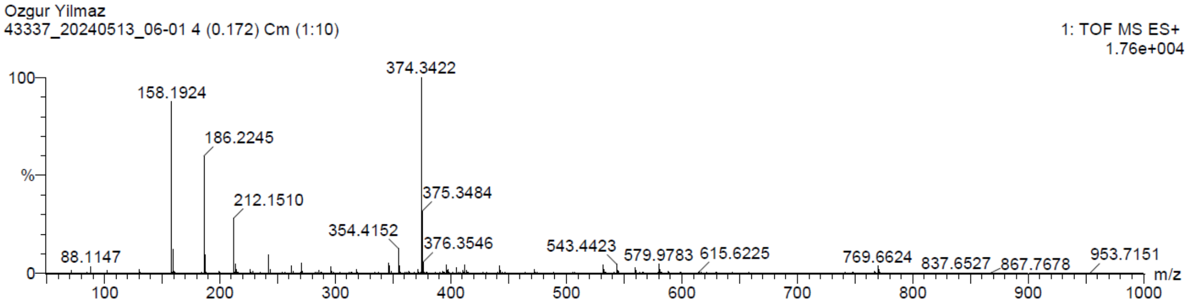

Minimum:				-5.5			
Maximum:		1000.0	1000.0	1000.0			
Mass	Calc. Mass	mDa	PPM	DBE	i-FIT	i-FIT (Norm)	Formula
374.3422	374.3423	-0.1	-0.3	4.5	835.9	0.0	C25 H44 N O

*Figure S52. HRMS result of (S)-N,N-dihexyl-2-(4-isobutylphenyl)propanamide **(5d).***

(S)-2-(4-isobutylphenyl)-*N,N*-dioctylpropanamide ($C_{29}H_{51}NO$, 6d)

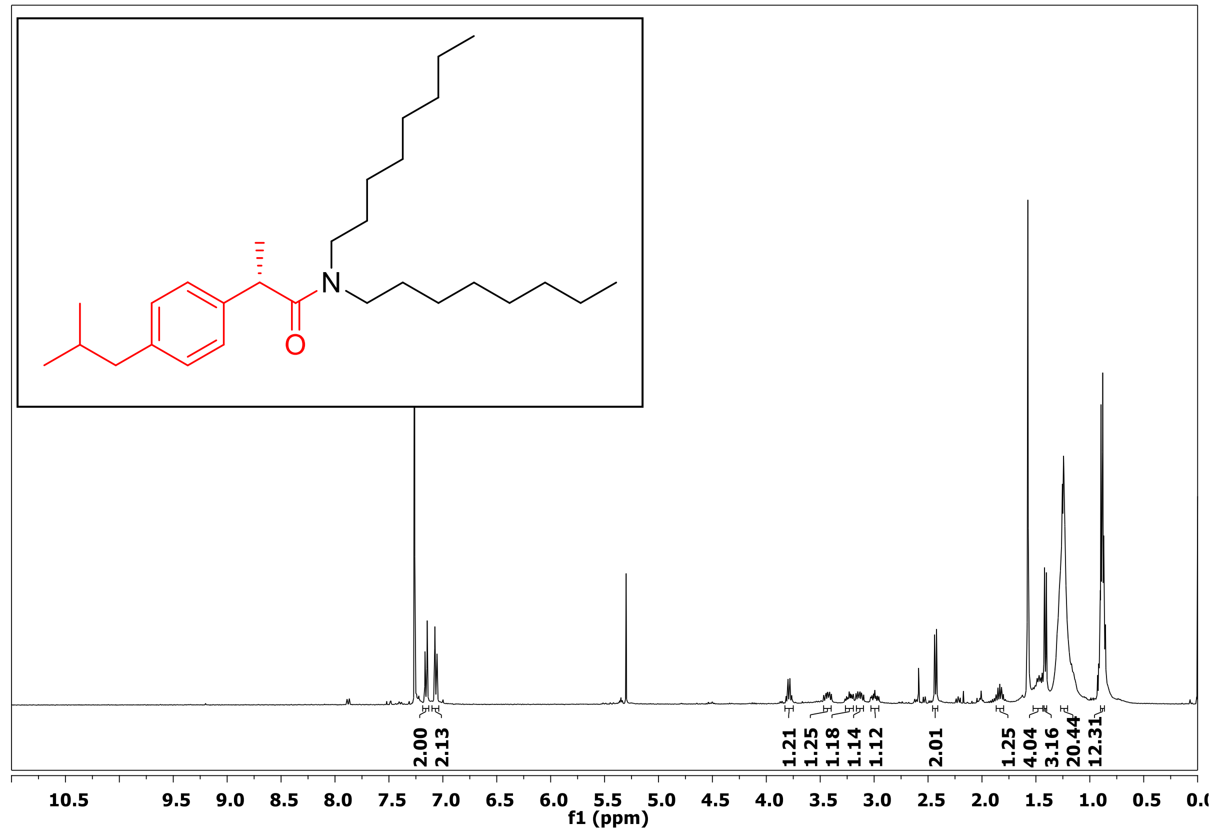

Figure S53. ¹H NMR spectrum of (S)-2-(4-isobutylphenyl)-N,N-dioctylpropanamide ***(6d)*** *($CDCl_3$), 400 MHz.*

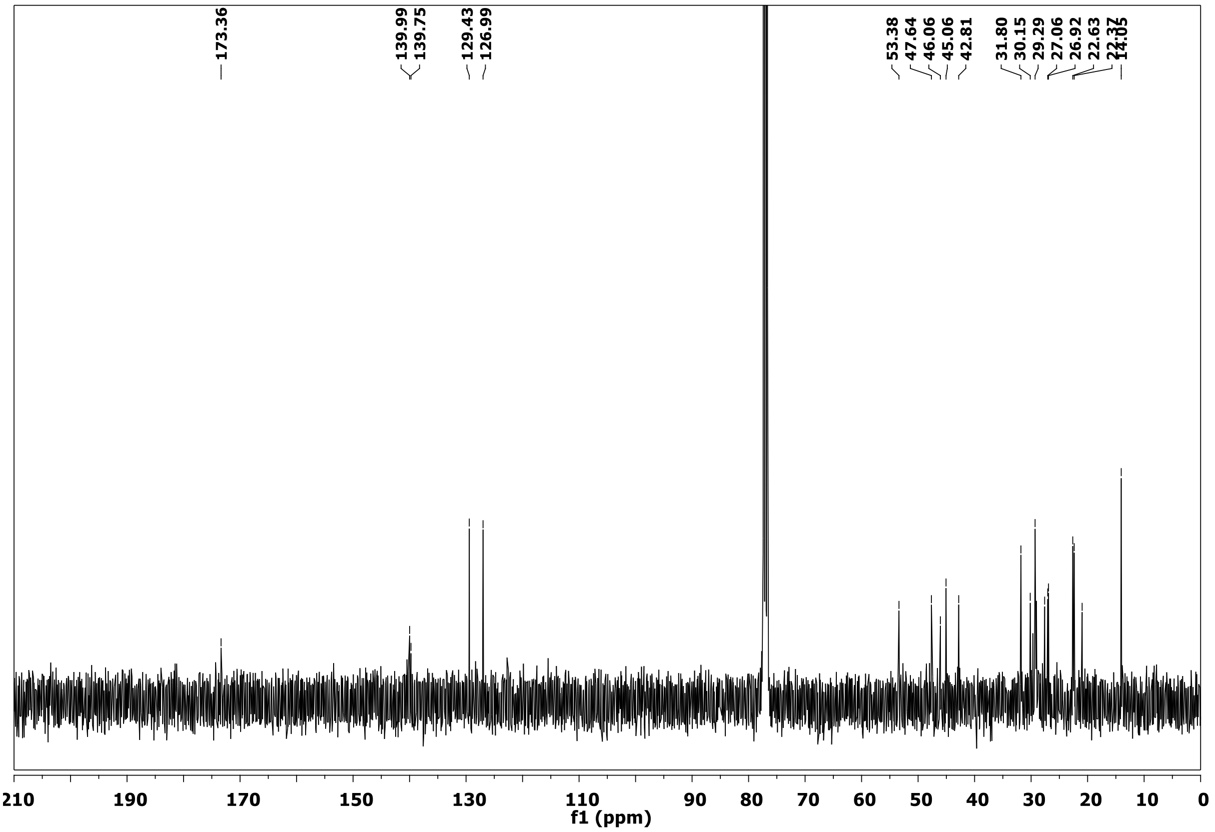

Figure S54. ¹³C{H} NMR spectrum of (S)-2-(4-isobutylphenyl)-N,N-dioctylpropanamide ***(6d)*** *($CDCl_3$), 101 MHz.*

Single Mass Analysis
Tolerance = 1000.0 PPM / DBE: min = -5.5, max = 1000.0
Element prediction: Off
Number of isotope peaks used for i-FIT = 9

Monoisotopic Mass, Even Electron Ions
1 formula(e) evaluated with 1 results within limits (all results (up to 1000) for each mass)
Elements Used:
C: 29-29 H: 51-52 N: 1-1 O: 1-1
Ozgur Yilmaz
43337_20240513_07-01 20 (0.775) Cm (15:25)

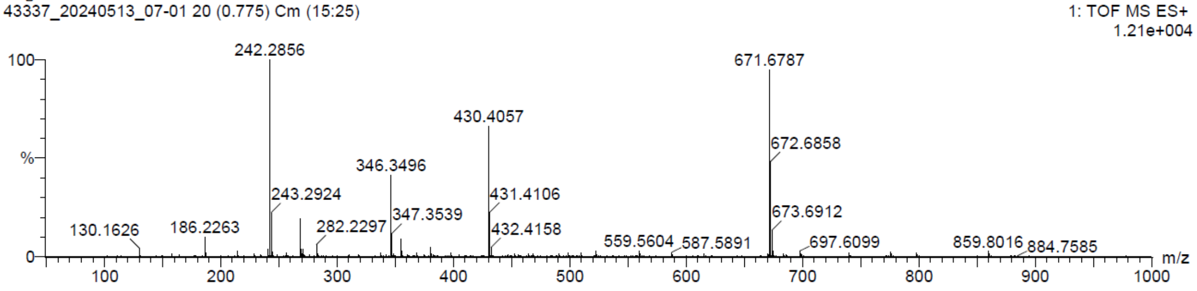

Minimum:				-5.5			
Maximum:		1000.0	1000.0	1000.0			
Mass	Calc. Mass	mDa	PPM	DBE	i-FIT	i-FIT (Norm)	Formula
430.4057	430.4049	0.8	1.9	4.5	724.7	0.0	C29 H52 N O

Figure S55. HRMS result of (S)-2-(4-isobutylphenyl)-N,N-dioctylpropanamide ***(6d).***

(S)-2-(4-isobutylphenyl)-1-morpholinopropan-1-one ($C_{17}H_{25}NO$, 7d)

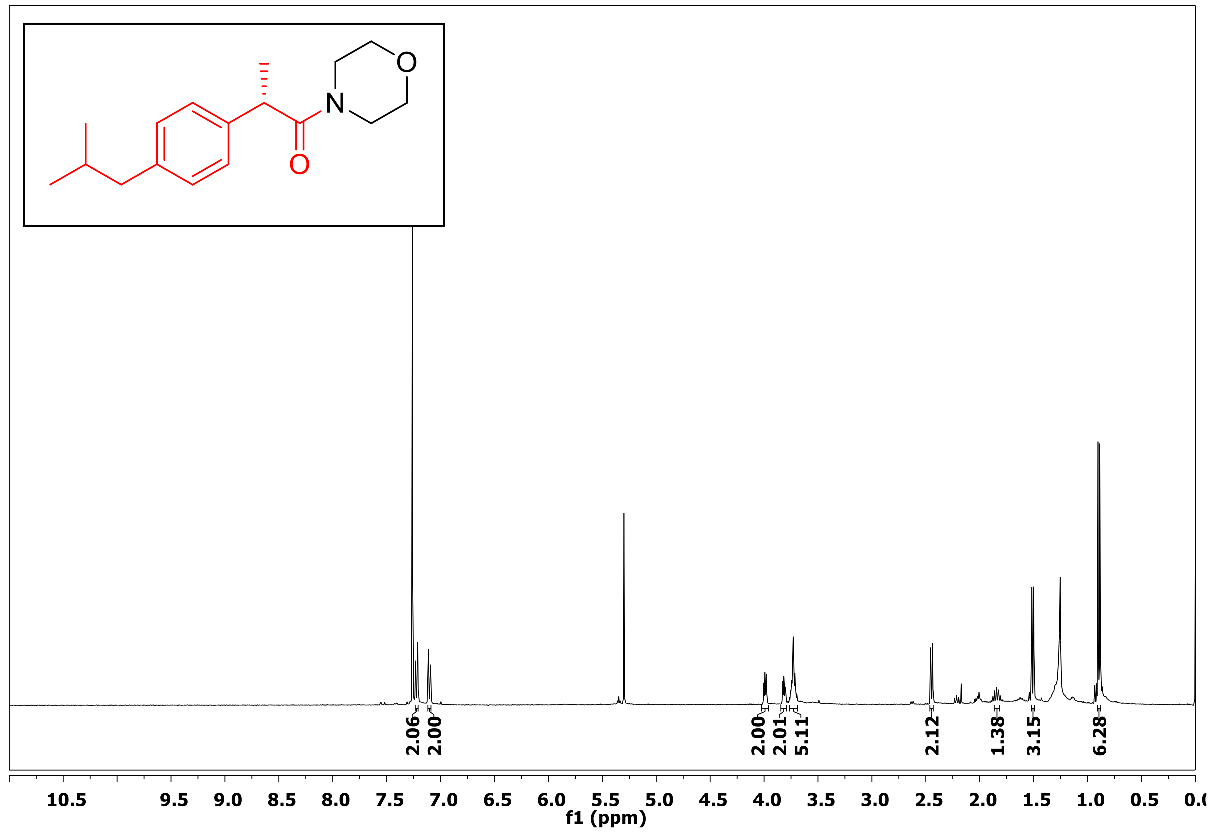

*Figure S56. ^{1}H NMR spectrum of (S)-2-(4-isobutylphenyl)-1-morpholinopropan-1-one **(7d)** ($CDCl_3$), 400 MHz.*

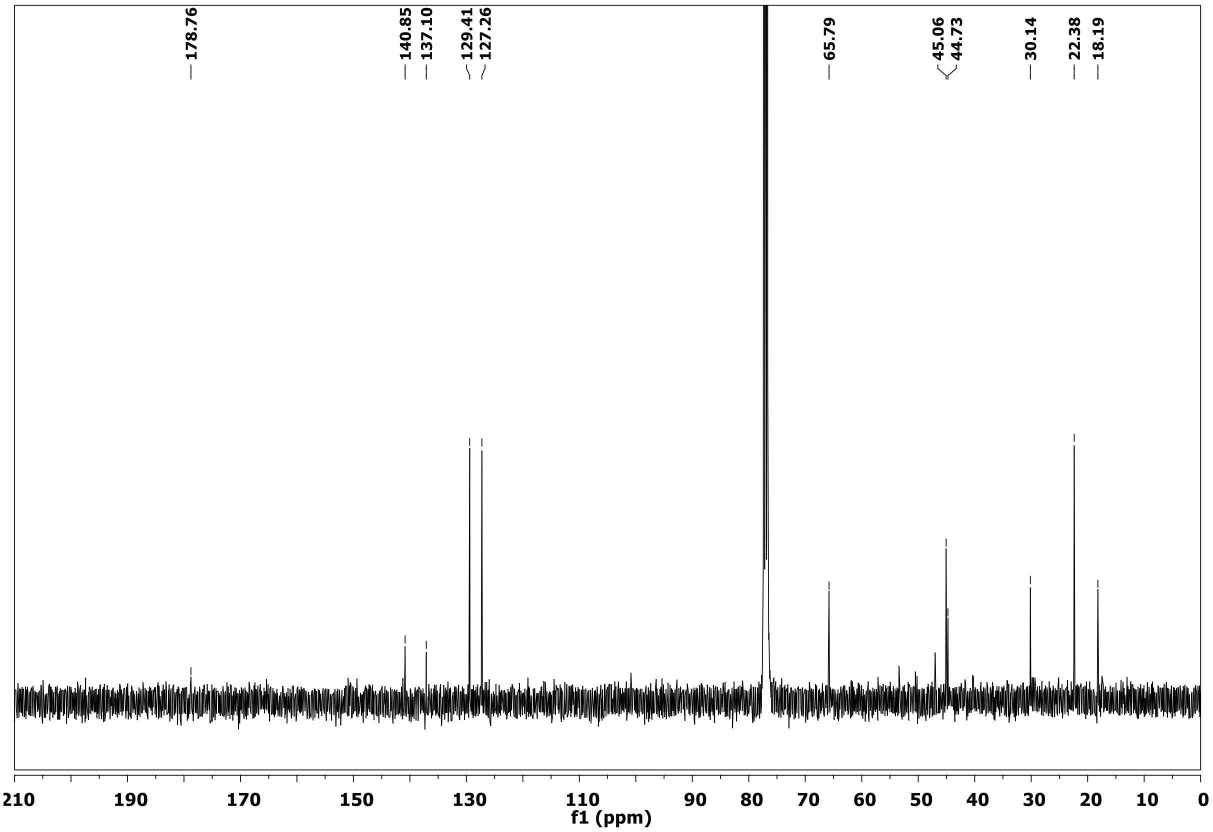

*Figure S57. ^{13}C{H} NMR spectrum of (S)-2-(4-isobutylphenyl)-1-morpholinopropan-1-one **(7d)** ($CDCl_3$), 101 MHz.*

Elemental Composition Report

Single Mass Analysis

Tolerance = 1000.0 PPM / DBE: min = -5.5, max = 1000.0
Element prediction: Off
Number of isotope peaks used for i-FIT = 9

Monoisotopic Mass, Even Electron Ions
1 formula(e) evaluated with 1 results within limits (all results (up to 1000) for each mass)
Elements Used:
C: 17-17 H: 25-26 N: 1-1 O: 2-2

Ozgur Yilmaz
43337_20240513_09-03 22 (0.863) Cm (18:24)

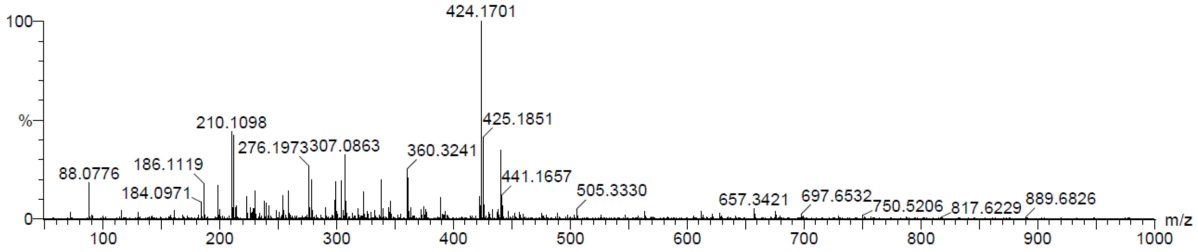

Minimum:				-5.5			
Maximum:		1000.0	1000.0	1000.0			
Mass	Calc. Mass	mDa	PPM	DBE	i-FIT	i-FIT (Norm)	Formula
276.1973	276.1964	0.9	3.3	5.5	869.6	0.0	C17 H26 N O2

Figure S58. HRMS result of (S)-2-(4-isobutylphenyl)-1-morpholinopropan-1-one ***(7d).***

(S)-2-(4-isobutylphenyl)-1-(piperidin-1-yl)propan-1-one ($C_{18}H_{27}NO$, 8d)

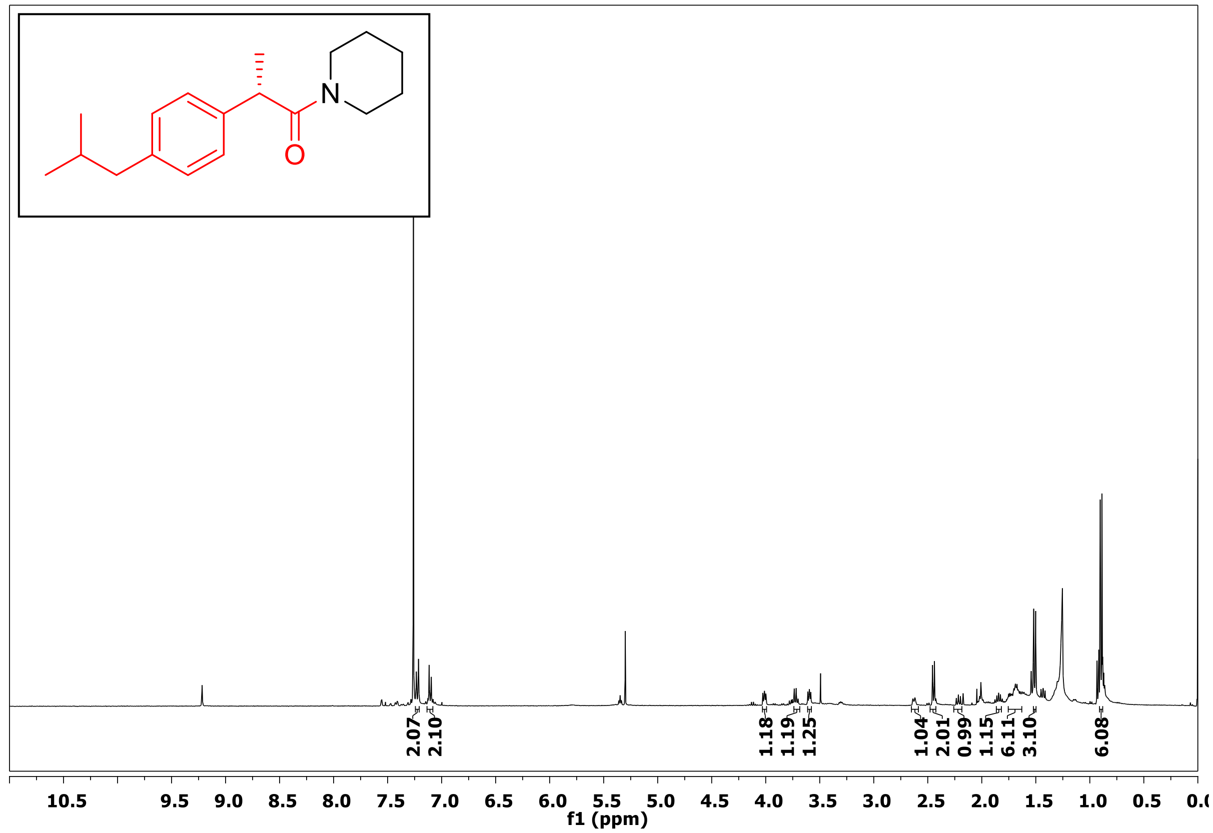

*Figure S59. ^{1}H NMR spectrum of (S)-2-(4-isobutylphenyl)-1-(piperidin-1-yl)propan-1-one **(8d)** ($CDCl_3$), 400 MHz.*

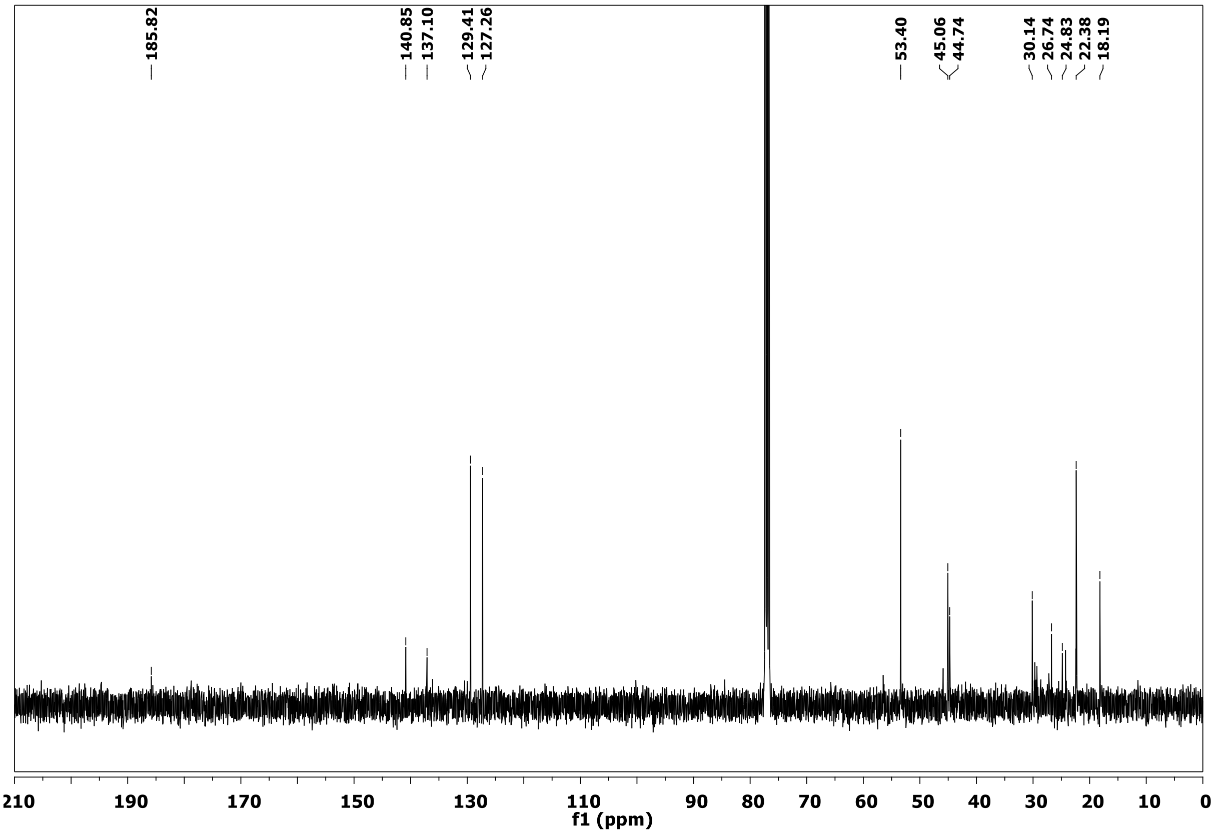

*Figure S60. ^{13}C{H} NMR spectrum of (S)-2-(4-isobutylphenyl)-1-(piperidin-1-yl)propan-1-one **(8d)** ($CDCl_3$), 101 MHz.*

Elemental Composition Report

Single Mass Analysis

Tolerance = 1000.0 PPM / DBE: min = -5.5, max = 1000.0
Element prediction: Off
Number of isotope peaks used for i-FIT = 9

Monoisotopic Mass, Even Electron Ions
1 formula(e) evaluated with 1 results within limits (all results (up to 1000) for each mass)
Elements Used:
C: 18-18 H: 27-28 N: 1-1 O: 1-1
Ozgur Yilmaz
43337_20240513_08-03 8 (0.328) Cm (1:15)

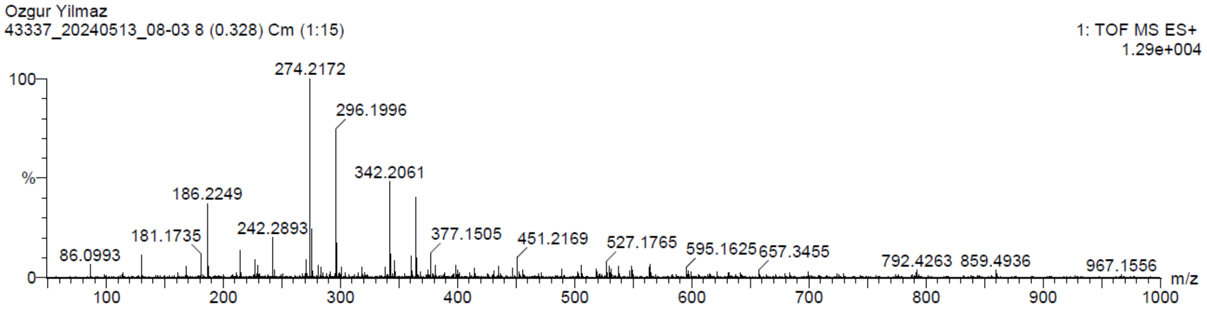

Minimum:				-5.5			
Maximum:		1000.0	1000.0	1000.0			
Mass	Calc. Mass	mDa	PPM	DBE	i-FIT	i-FIT (Norm)	Formula
274.2172	274.2171	0.1	0.4	5.5	860.2	0.0	C18 H28 N O

Figure S61. HRMS result of (S)-2-(4-isobutylphenyl)-1-(piperidin-1-yl)propan-1-one ***(8d).***

Comparison of ^{19}F NMR spectra for control reactions

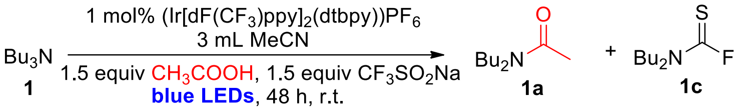

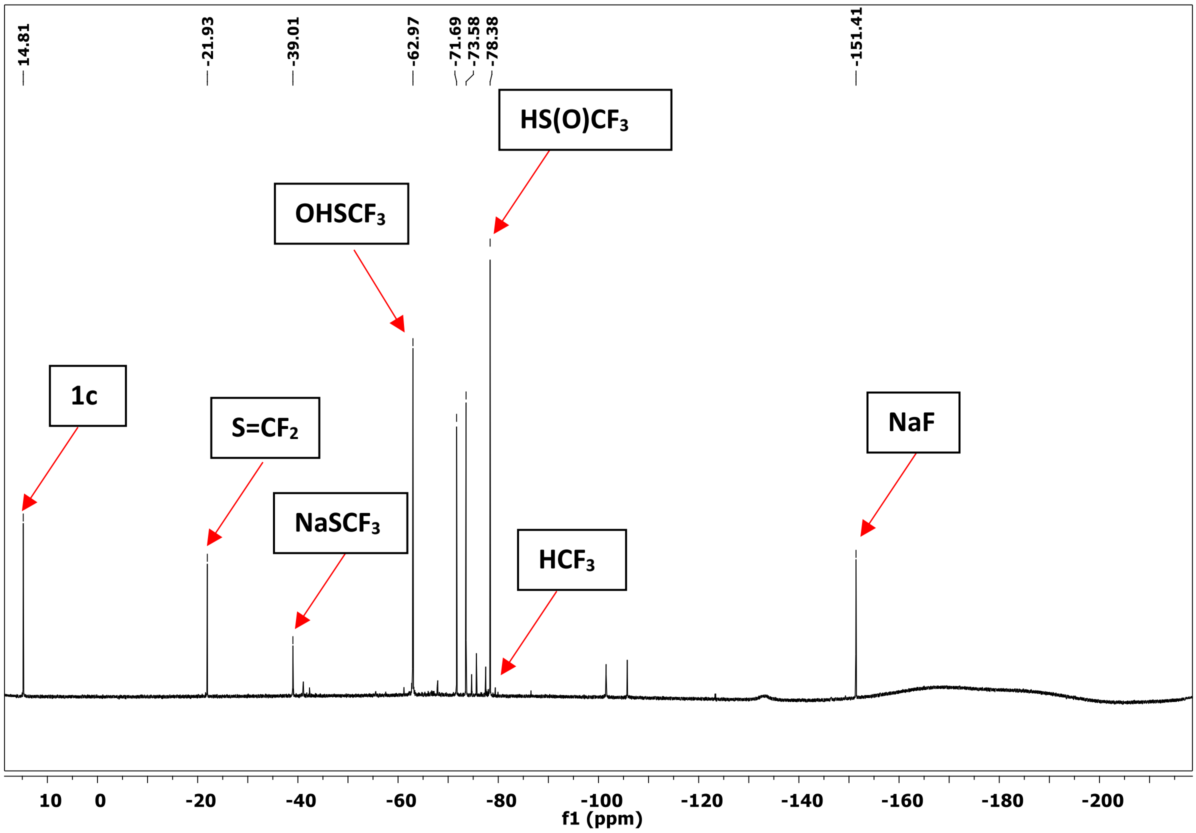

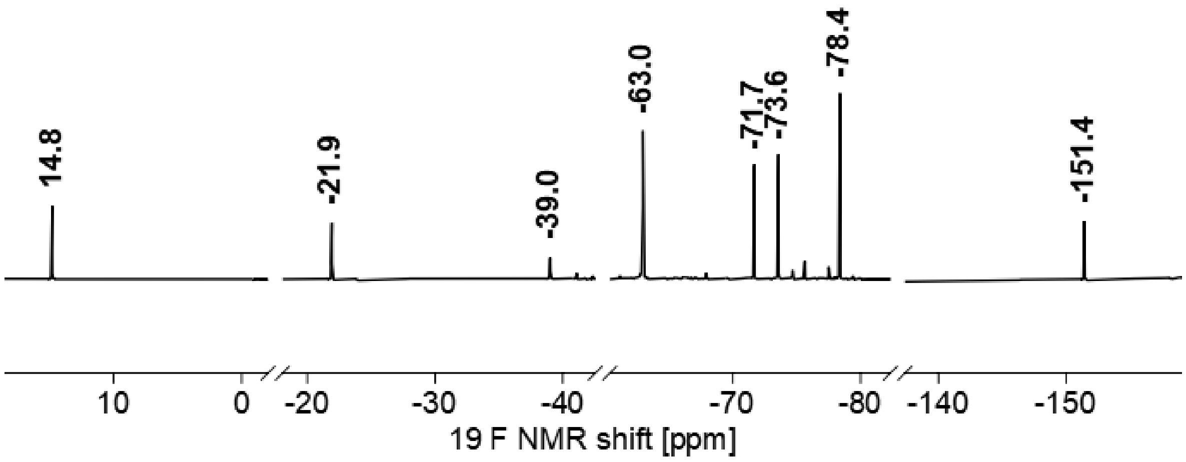

Figure S62. ^{19}F NMR spectra of representative reaction

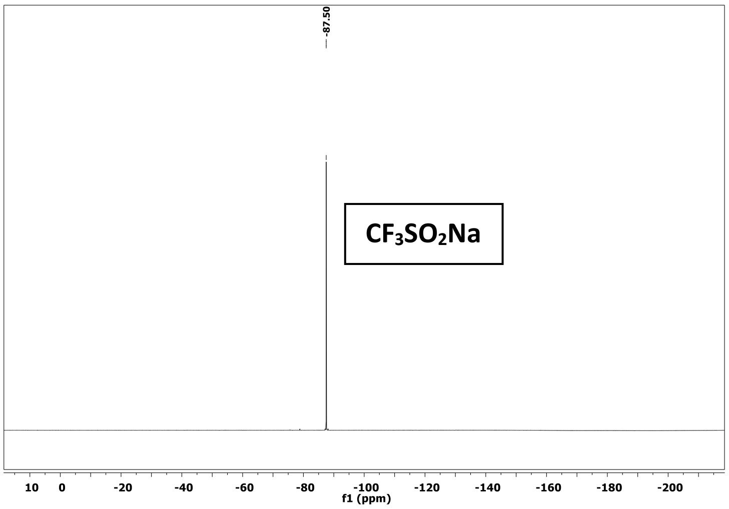

Figure S63. ^{19}F NMR spectra of CF_3SO_2Na

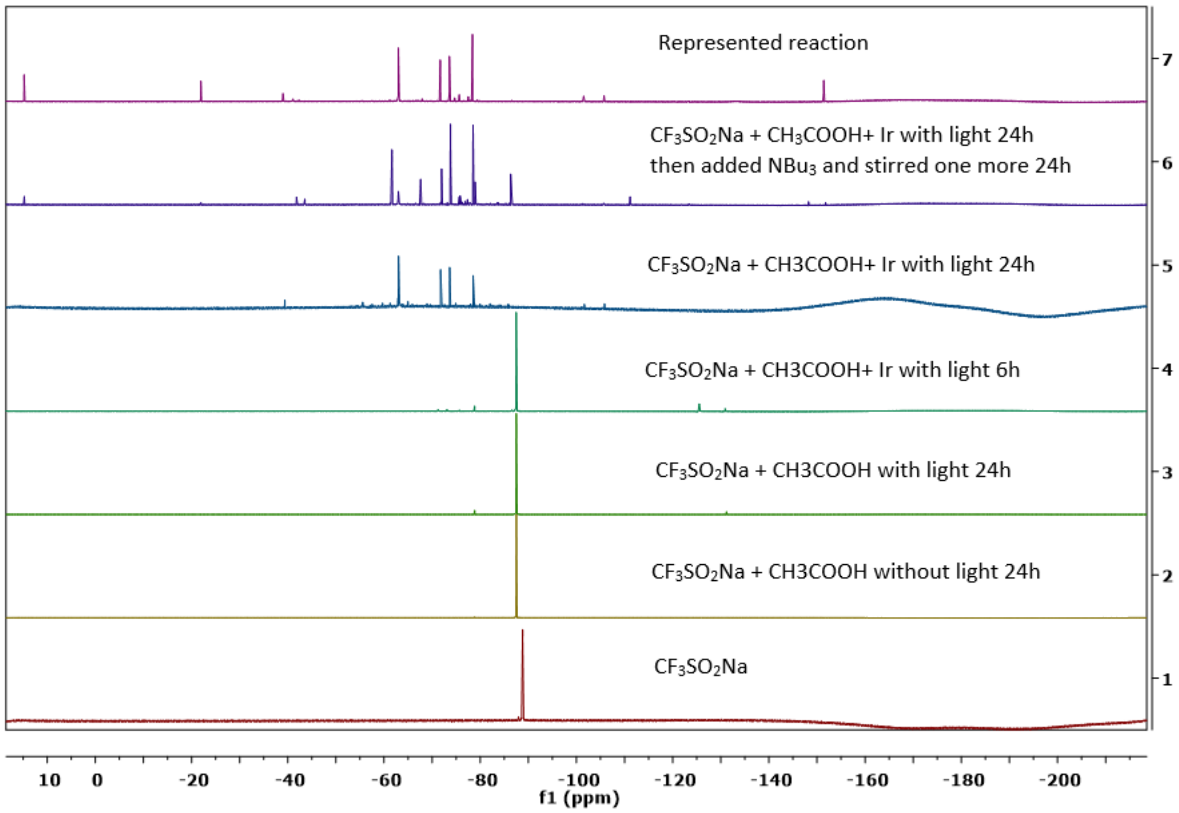

Figure S64. Stacked Plot Comparison of ^{19}F NMR spectra of control reactions

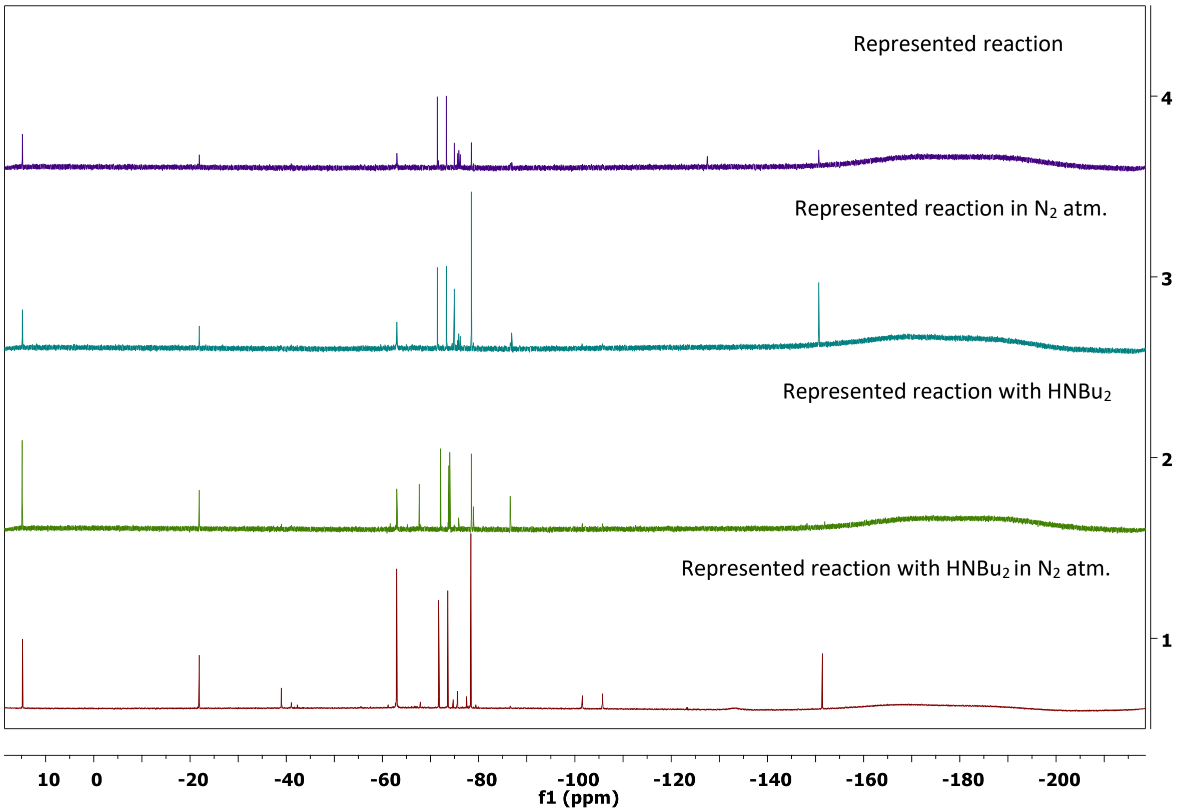

Figure S65. Stacked Plot Comparison of ^{19}F NMR spectra of represented reactions with NBu_3 and $HNBu_2$ in O_2 atm. or N_2 atm.

** For the reaction in N_2 atm., a septum cap allowing syringe inlet was used. Nitrogen was passed through the reaction medium for 30 min with a nitrogen-filled balloon connected to the syringe, then the syringe was removed and the reaction was continued.

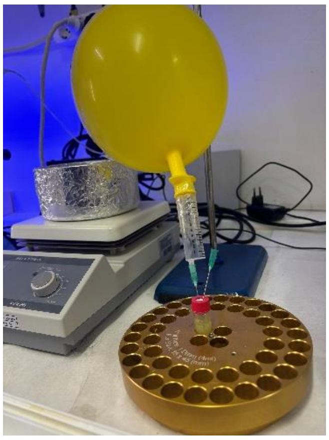

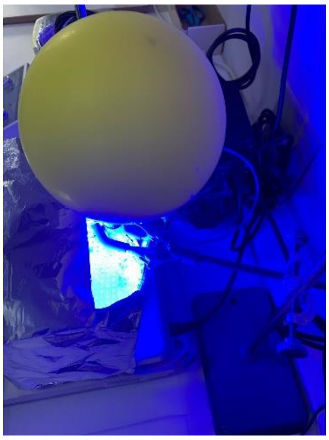

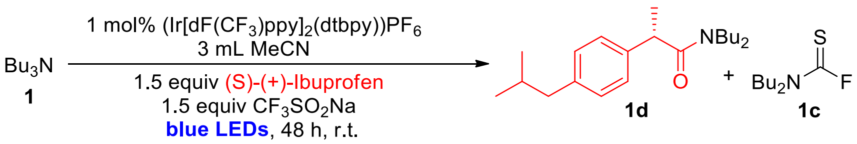

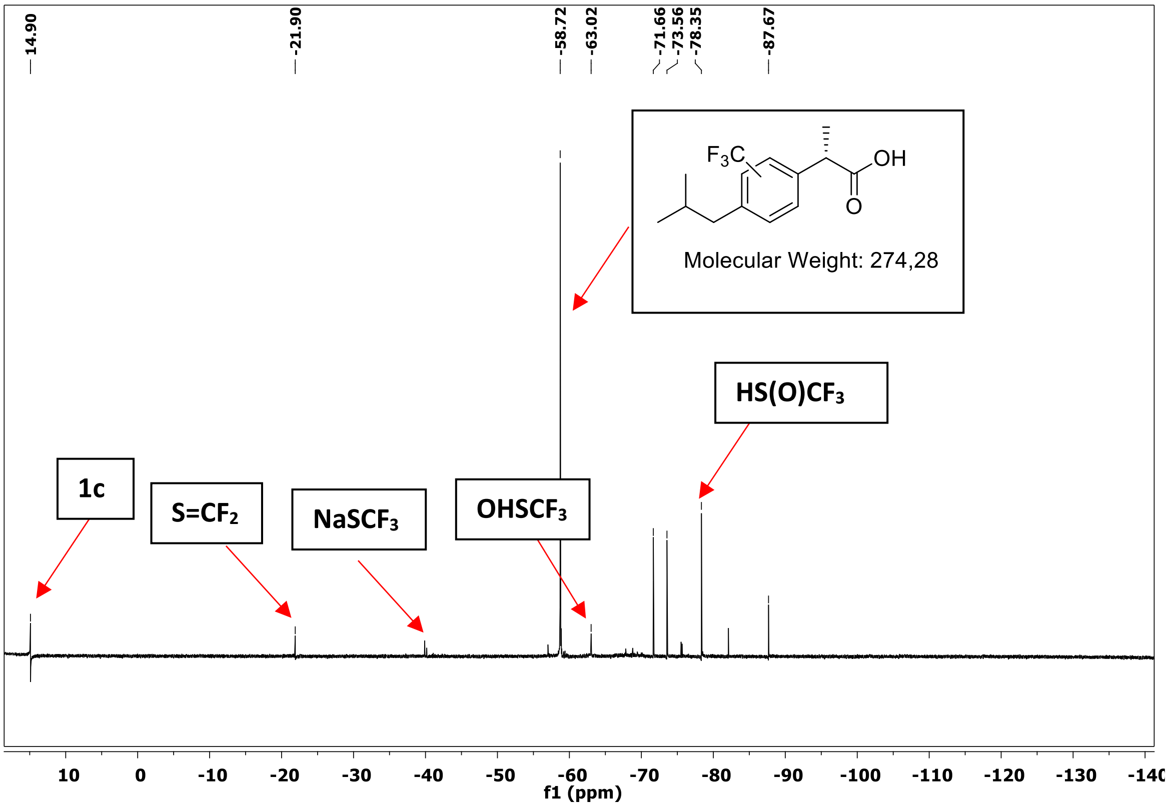

Figure S66. ^{19}F NMR spectra of represented reactions with S-ibuprofen

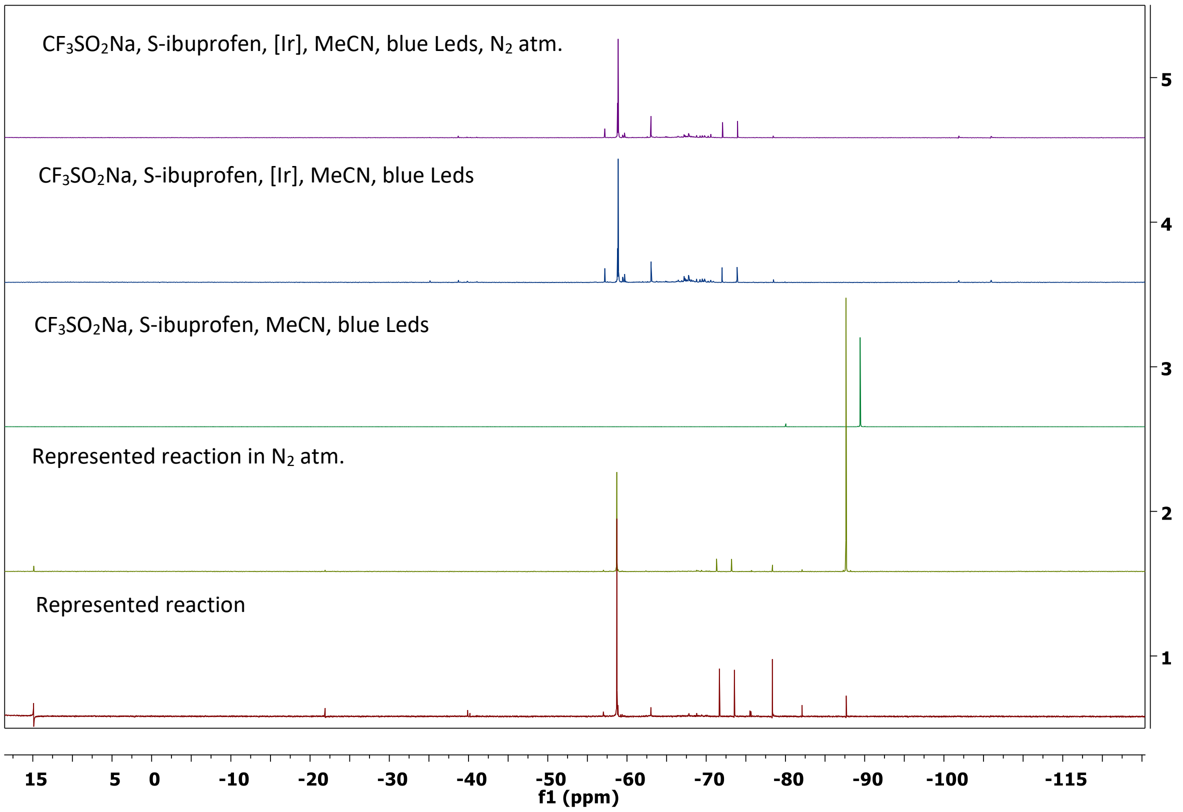

Figure S67. Stacked Plot Comparison of ^{19}F NMR spectra of control reactions with S-ibuprofen

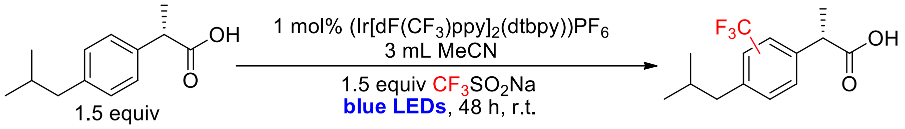

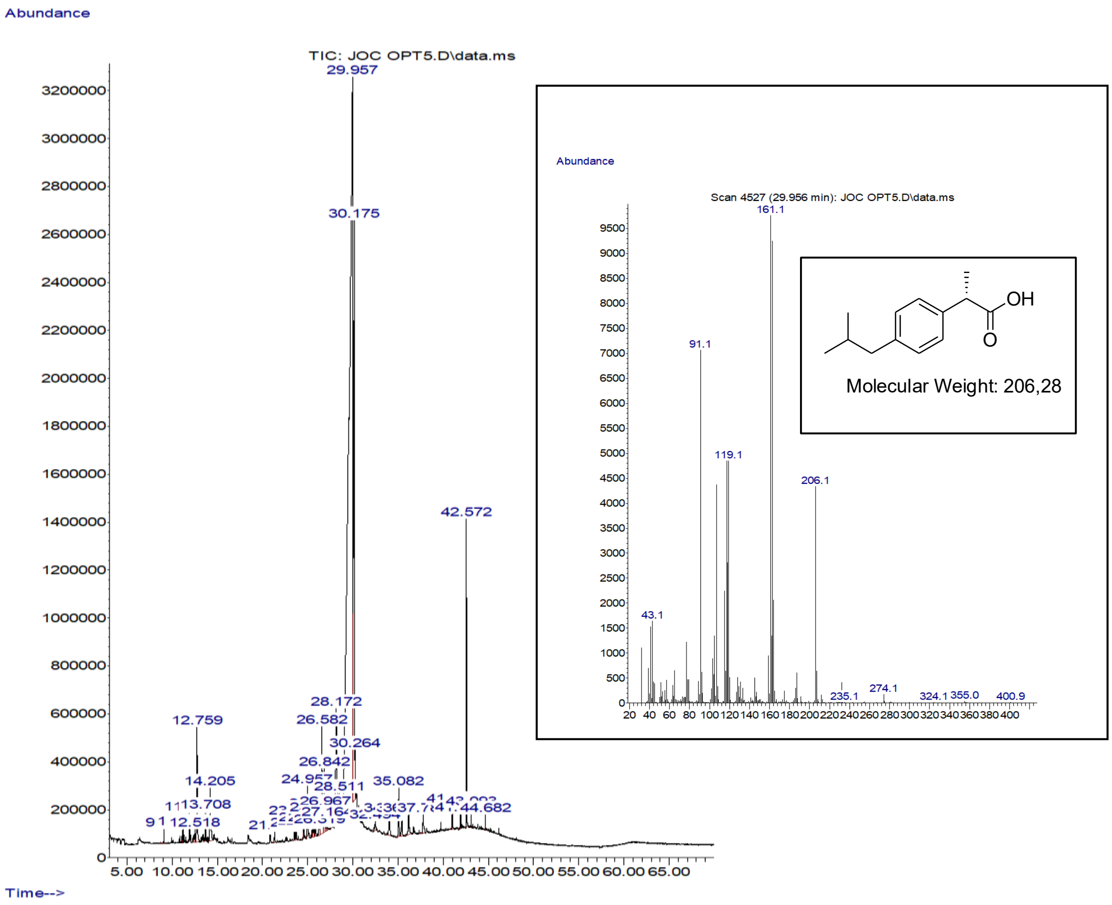

Figure S68. Chromatogram and peak assignments for crude reaction S-ibuprofen, CF_3SO_2Na, [Ir] and blue leds (obtained same products under N_2 condition)

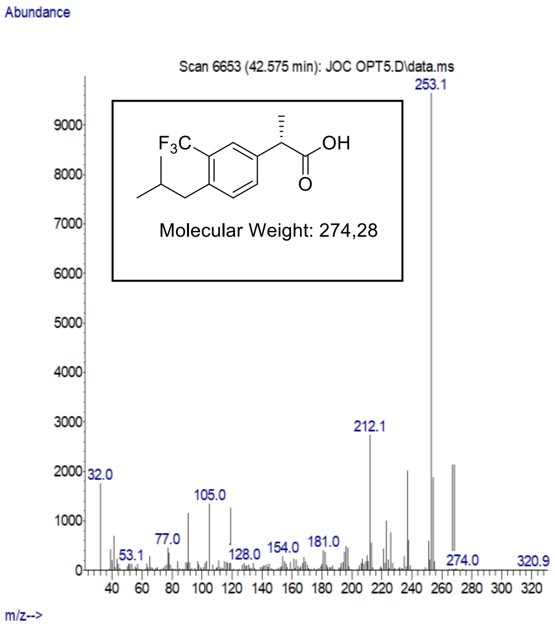

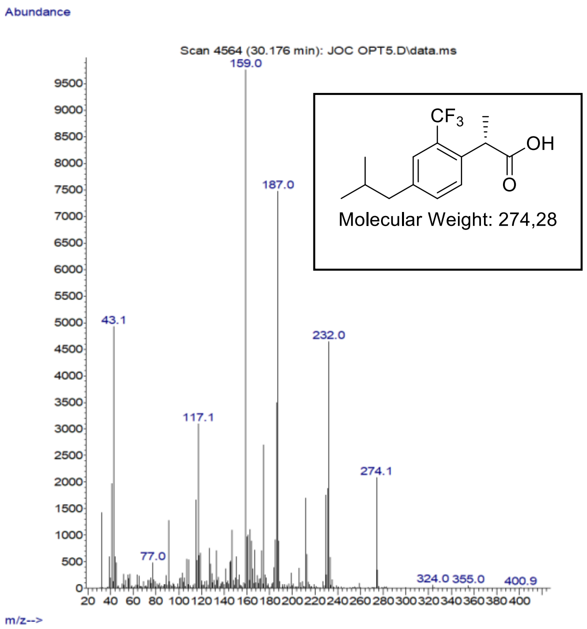

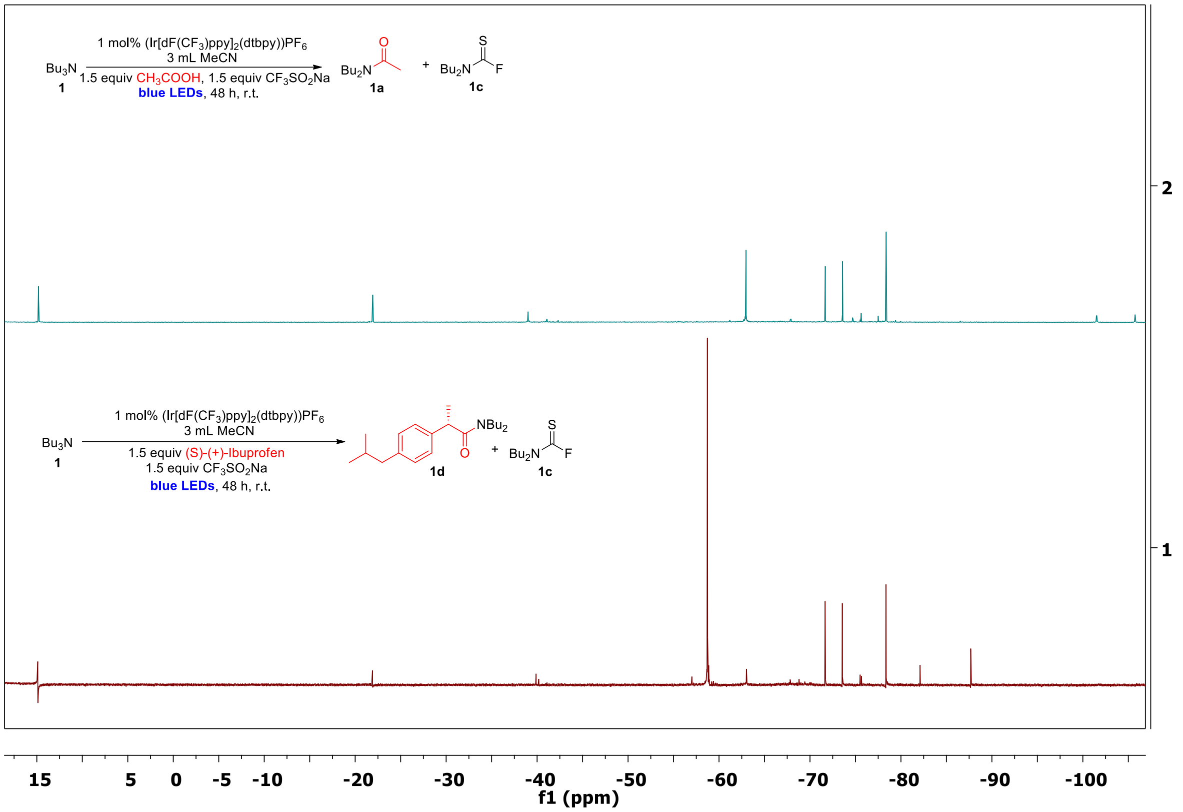

Figure S69. Stacked Plot Comparison of ^{19}F NMR spectra of reaction NBu_3 with CH_3COOH or S-ibuprofen

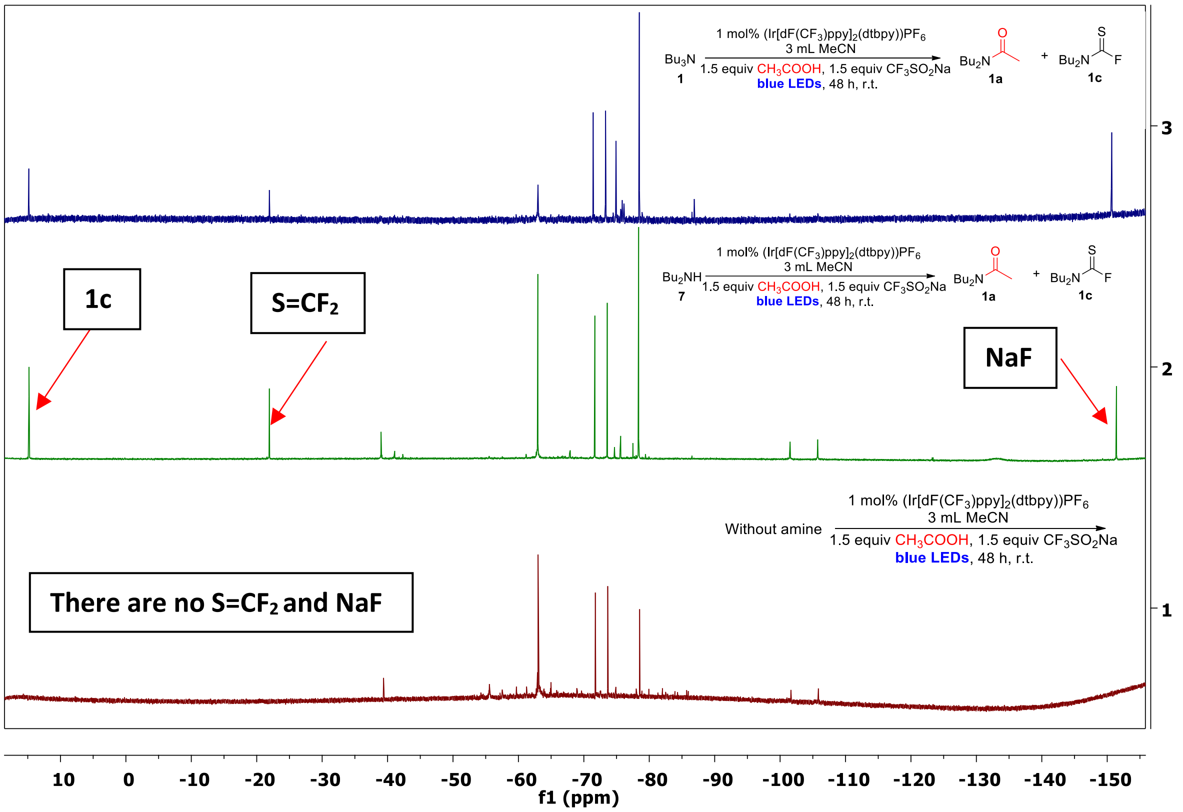

Figure S70. Stacked Plot Comparison of ^{19}F NMR spectra of represented reaction with NBu_3 or $HNBu_2$ and without amine

Comparison of ^{19}F NMR spectra at different reaction times

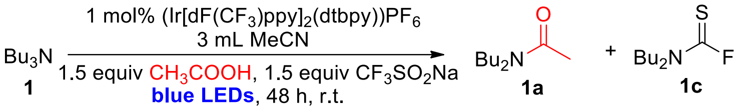

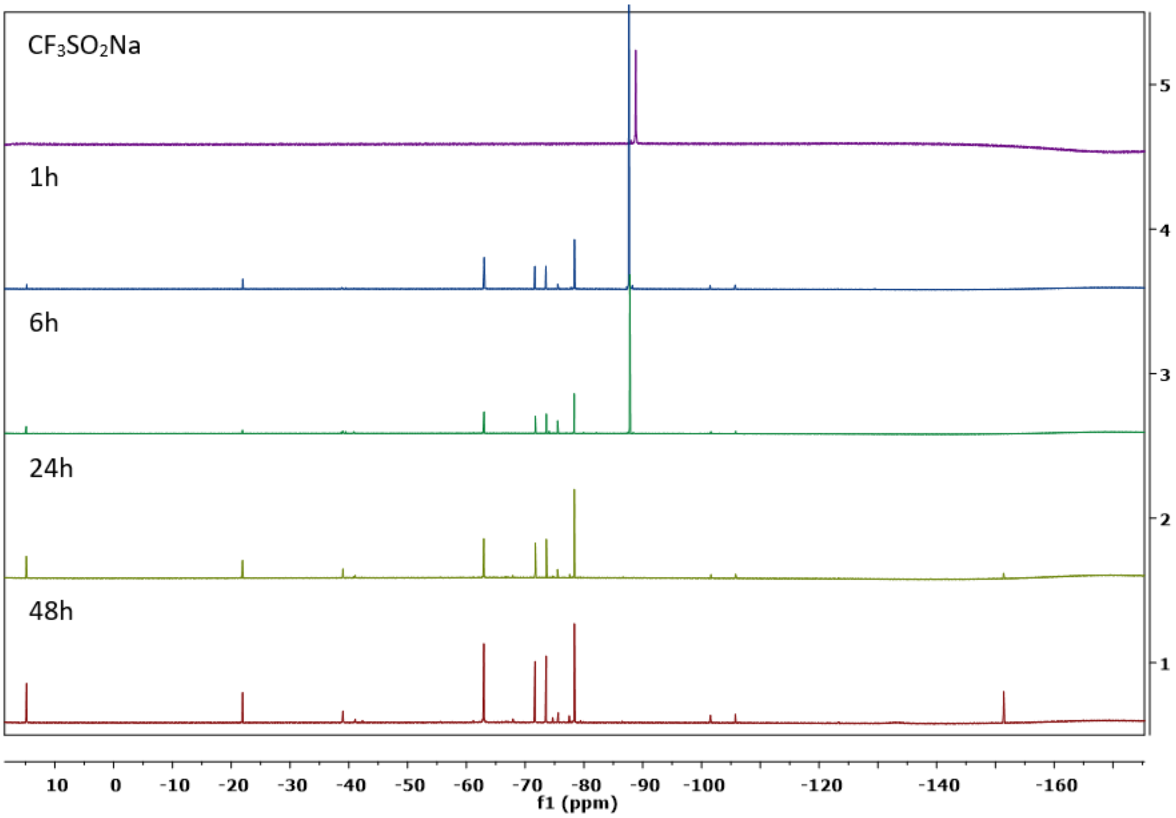

Figure S71. Stacked Plot Comparison of ^{19}F NMR spectra of reaction with NBu_3 after Irradiation for 1h, 6h, 24h and 48h.

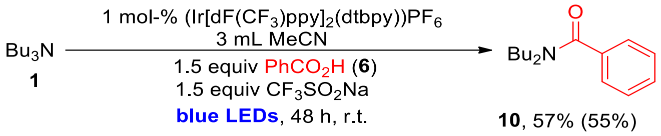
Bu3N
1
1 mol-% (Ir[dF(CF3)ppy]2(dtbpy))PF6
3 mL MeCN
1.5 equiv PhCO2H (6)
1.5 equiv CF3SO2Na
blue LEDs, 48 h, r.t.
Bu2N
O
10, 57% (55%)

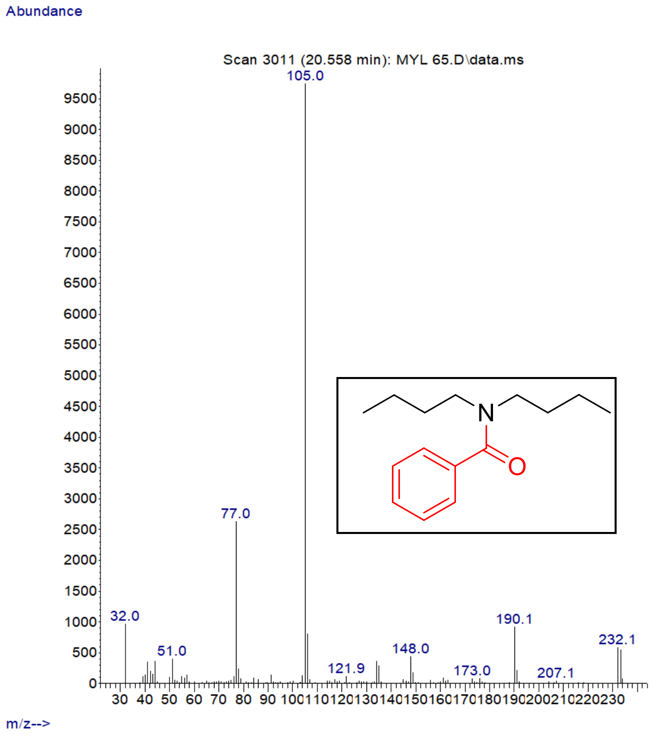
Abundance
Scan 3011 (20.558 min): MYL 65.D\data.ms
105.0
77.0
32.0
51.0
121.9
148.0
173.0
190.1
207.1
232.1
N
O
m/z-->

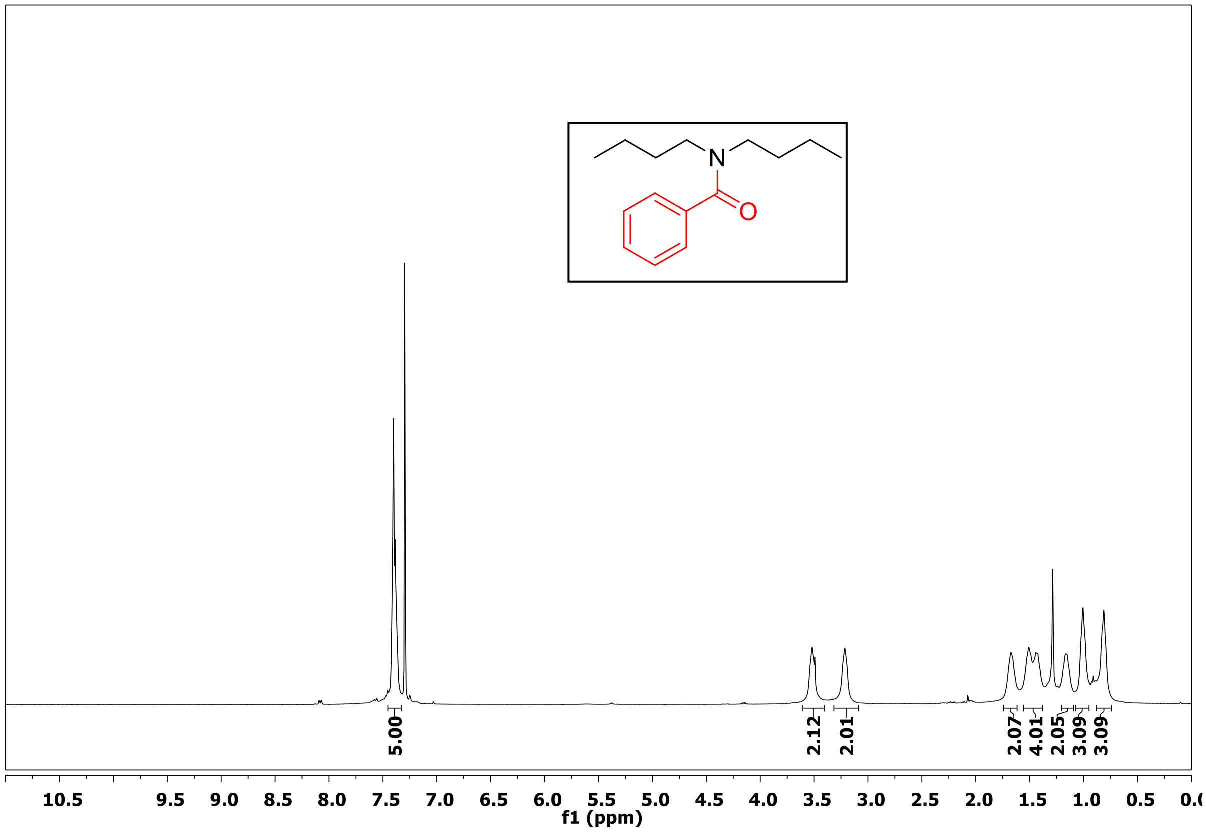
N
O
5.00
2.12
2.01
2.07
4.01
2.05
3.09
3.09
f1 (ppm)

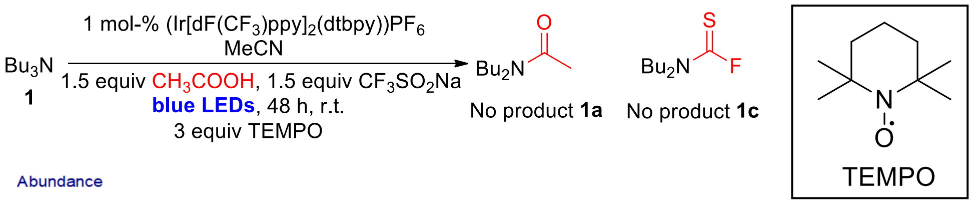

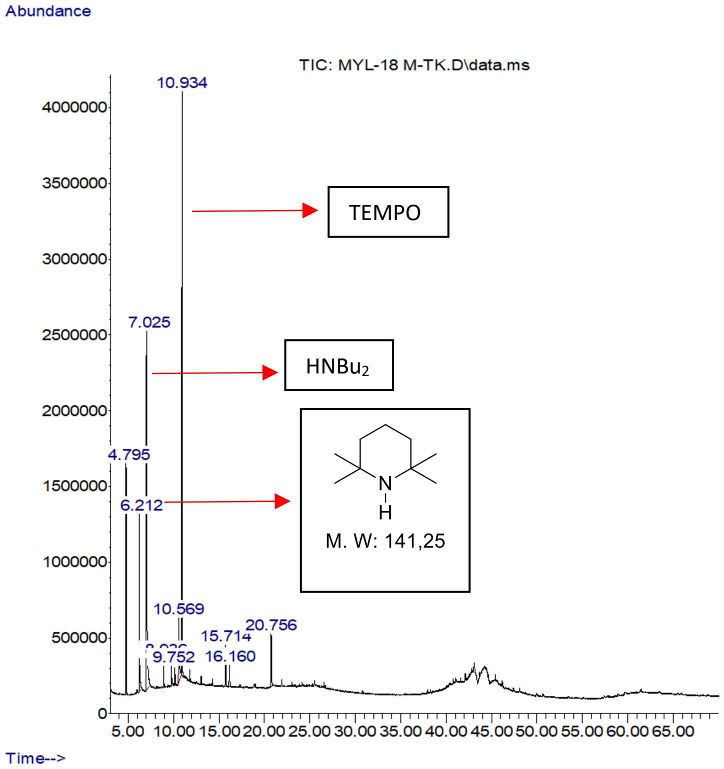

Figure S72. GCMS Chromatogram and peak assignments for crude reaction

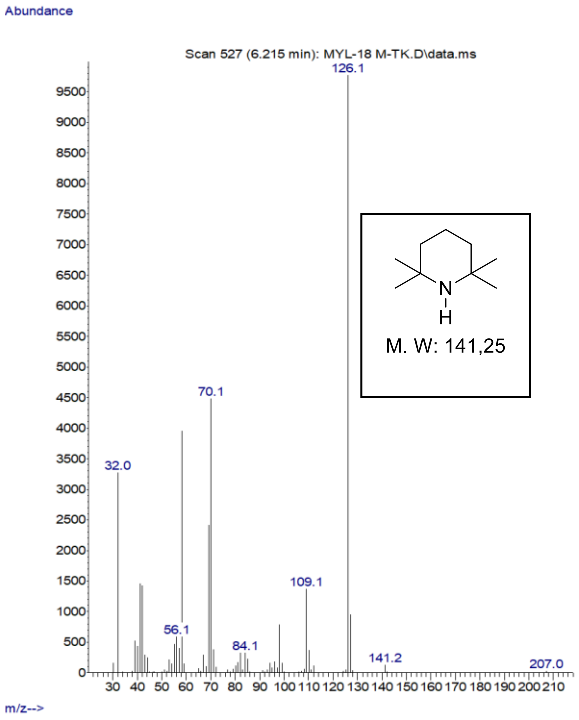

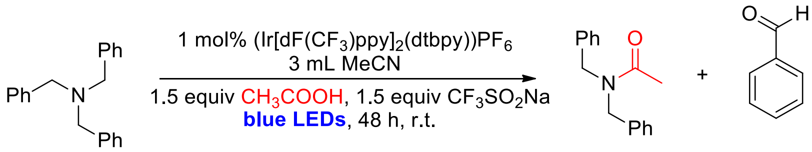
1 mol% (Ir[dF(CF3)ppy]2(dtbpy))PF6
3 mL MeCN
1.5 equiv CH3COOH, 1.5 equiv CF3SO2Na
blue LEDs, 48 h, r.t.
Ph
N
O
H
+

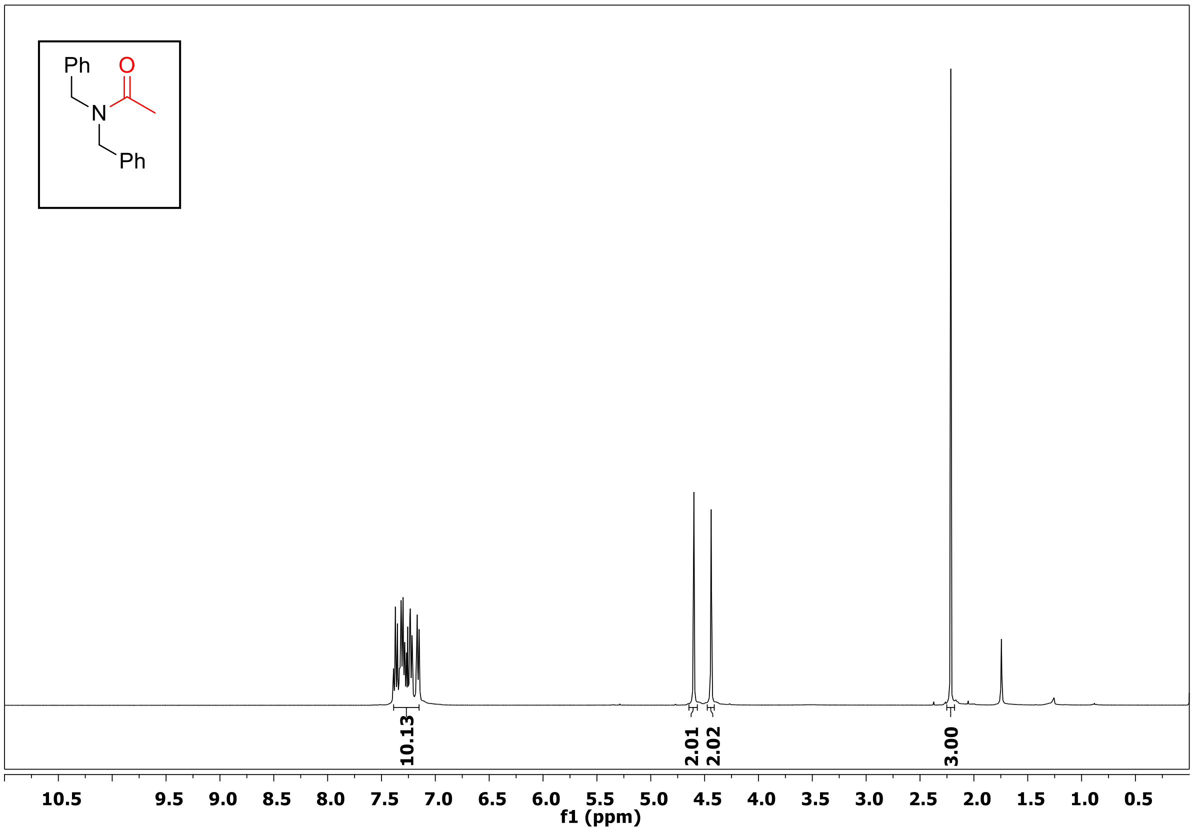
Ph
O
N
10.13
2.01
2.02
3.00
10.5
9.5
9.0
8.5
8.0
7.5
7.0
6.5
6.0
5.5
5.0
4.5
4.0
3.5
3.0
2.5
2.0
1.5
1.0
0.5
f1 (ppm)

Procedure for reactions of complex acids with *N*-methyl morpholine

1.5 equiv of each tested acid (40.5 µmol) was weighed into a 4 mL vial under an air atmosphere. 81 µL of MeCN was added to each vial and the vial was equipped with a stirbar.

A stock solution of 113 mg (123 µL, 1122 µmol) of *N*-methyl morpholine in 2121 µL MeCN was prepared. A stock solution of (Ir[dF(CF_3)ppy]$_2$(dtbpy))PF_6 (28.5 mg, 25.4 µmol) in 1243 µL MeCN was prepared. A stock solution of 478 mg (3066 µmol) CF_3SO_2Na in 5.65 mL MeCN was prepared.

54 µL of the *N*-methyl morpholine stock solution (corresponding to 1.0 equiv, 27 µmol) was added to each vial. 27 µL of (Ir[dF(CF_3)ppy]$_2$(dtbpy))PF_6 stock solution (corresponding to 2 mol %, 0.5 µmol) was added to each vial. 162 µL of CF_3SO_2Na stock solution (corresponding to 3.0 equiv, 81 µmol) was added to each vial.

The vials were sealed with teflon-lined caps and placed into a photoredox block designed for 1 dram vials (1 DRAM 24-Position, Photoredox, 18mm spacing; SKU 24626; Analytical Sales & Services). 3 lightshields (Analytical Sales & Services; SKU LIGHTSHIELD) were installed; orange tinted safety goggles were worn whenever in the vicinity of the actively irradiating reactor. The vials were then irradiated with a Lumidox® II 24-Well, 18mm Spacing LED array (470-BLUE LED Array, with Lens Mat and Active Base) at 270 nm (209 mW/well) for 72 h. The light source was switched off and the slightly warm plate (55 °C internal plate temperature) was allowed to cool to room temperature.

10 µL of the obtained reaction mixtures was diluted with 250 µL MeCN and 500 µL DMSO in an analytical 96-well sample plate. The plate was sealed and centrifuged for 5 min to settle any solids. Then, the crude reactions were analyzed by UPLCMS (MeCN/H_2O gradient, 2.0 min method, TFA modifier).

Reaction outcomes were analyzed with Virscidian Analytical Studio, primarily using traces at 254 nm and extracted product mass chromatograms.

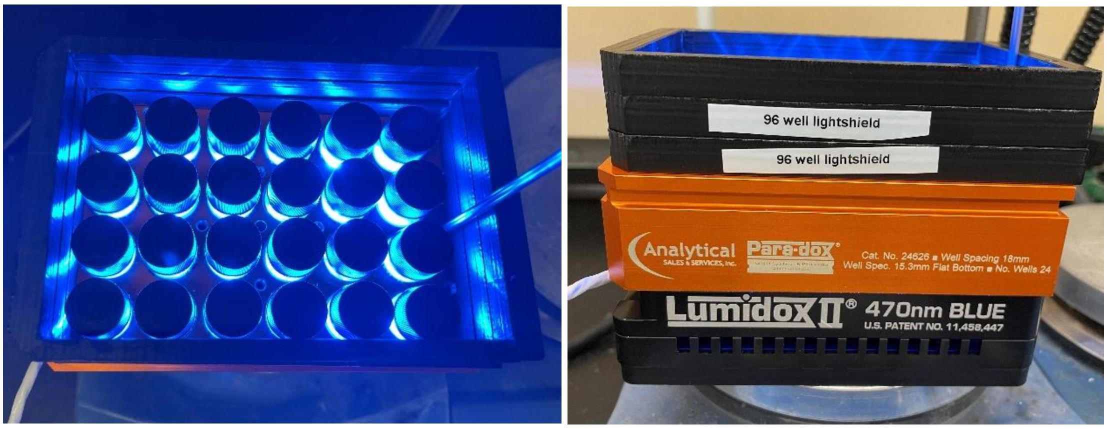

Figure S 73. Reactor setup. Left: Top view with irradiation. Right: Front view with irradiation.

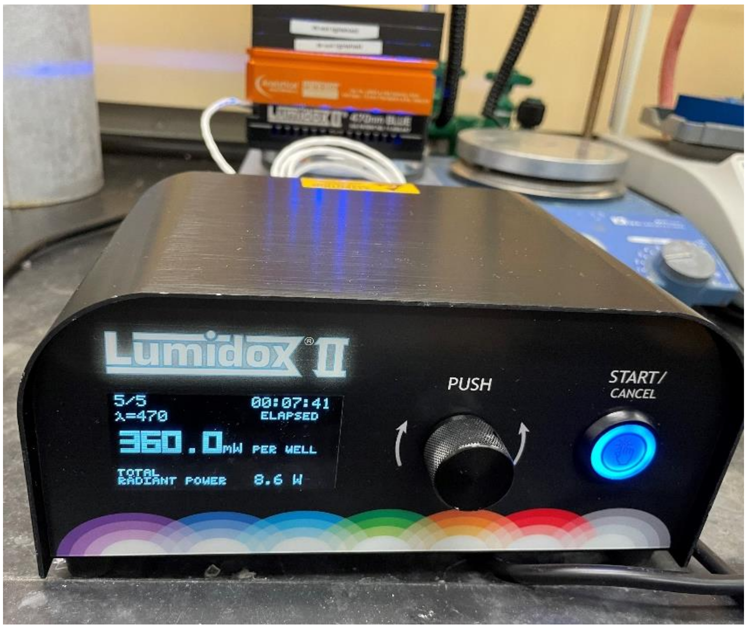

Figure S74. Front view of assembled reactor and LED array (background) together with Lumidox II power controller. All equipment is commercially available on the Analytical Sales & Services website.

LCMS data for reactions of complex acids with N-methyl morpholine

1,3-Dimethyl-7-(2-morpholino-2-oxoethyl)-1H-purine-2,6(3H,7H)-dione (11a)

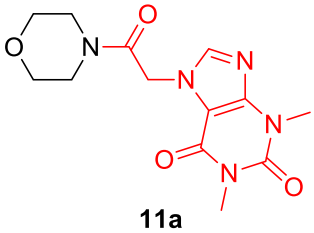

11a

Exact Mass: 307,13
Molecular Weight: 307,31
m/z: 307.13 (100.0%), 308.13 (16.3%), 309.13 (2.0%)

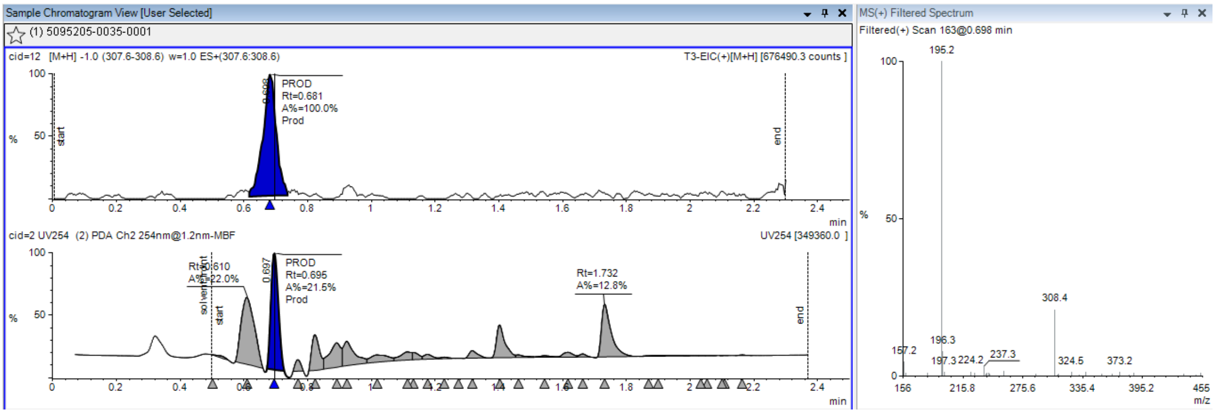

Anthracen-9-yl(morpholino)methanone (12a)

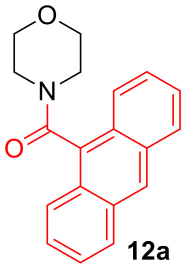

12a

Exact Mass: 291,13
Molecular Weight: 291,34
m/z: 291.13 (100.0%), 292.13 (20.8%), 293.13 (2.5%)

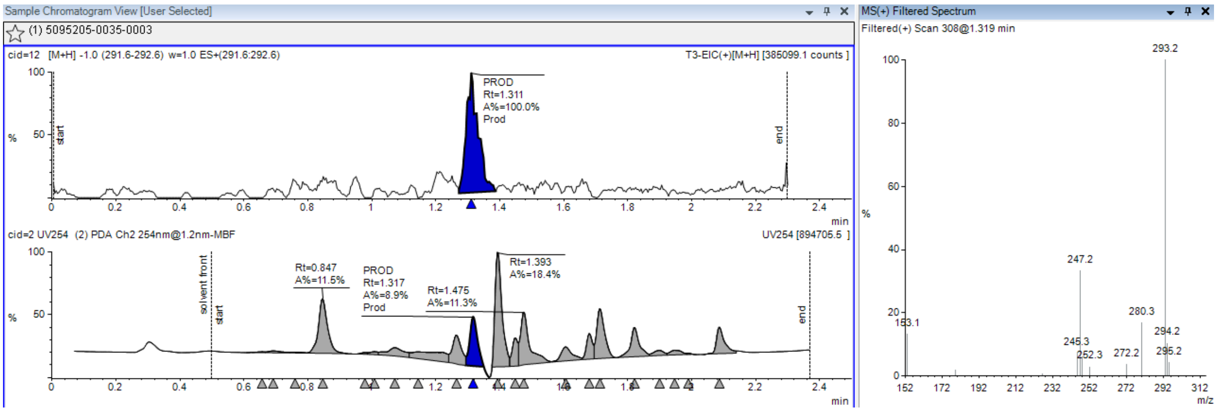

(2-Methyl-2,4,5,6-tetrahydrocyclopenta[c]pyrazol-3-yl)(morpholino)methanone (13a)

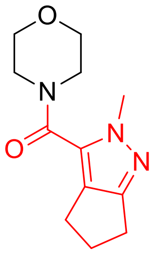

13a

Exact Mass: 235,13
Molecular Weight: 235,28
m/z: 235.13 (100.0%), 236.14 (13.3%), 237.14 (1.2%), 236.13 (1.1%)

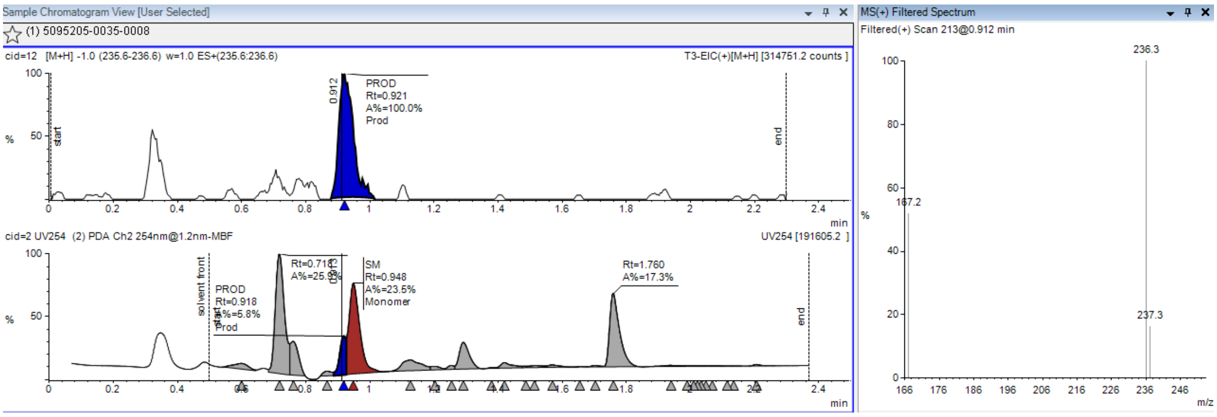

Morpholino(2-phenylimidazo[1,2-a]pyridin-3-yl)methanone (14a)

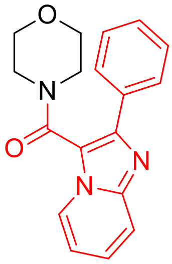

14a

Exact Mass: 307,13
Molecular Weight: 307,35
m/z: 307.13 (100.0%), 308.14 (19.7%), 309.14 (2.3%), 308.13 (1.1%)

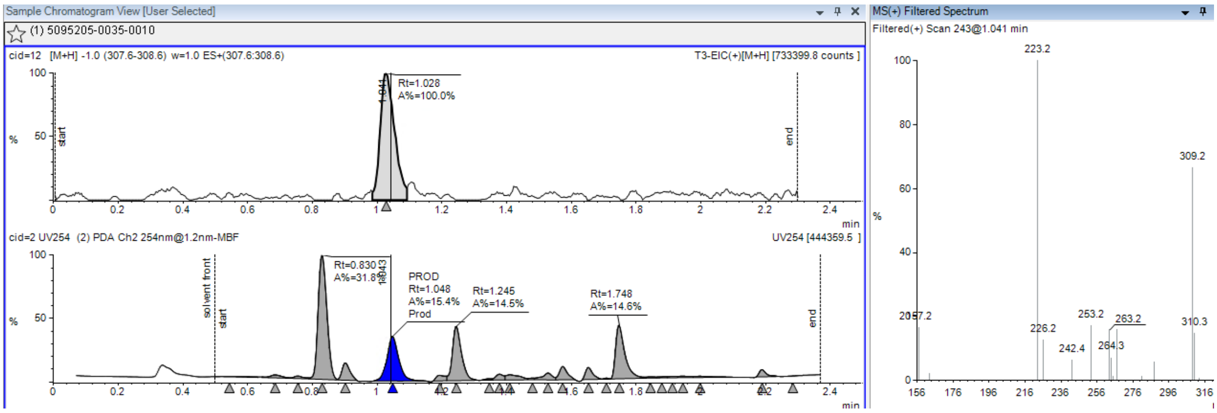

(2,5-Dichloro-4,6-dimethylpyridin-3-yl)(morpholino)methanone (15a)

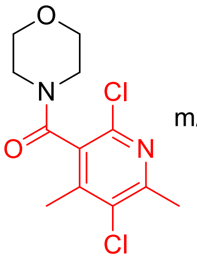

15a

Exact Mass: 288,04
Molecular Weight: 289,16
m/z: 288.04 (100.0%), 290.04 (64.0%), 289.05 (13.2%), 292.04 (10.5%), 291.04 (8.8%), 293.04 (1.4%), 290.05 (1.2%)

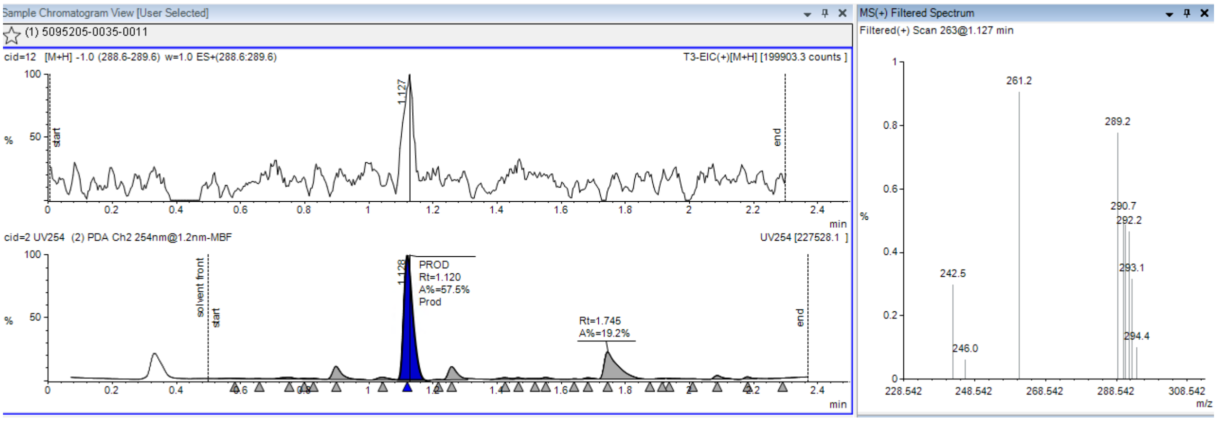

[1,2,4]Triazolo[1,5-a]pyrimidin-6-yl(morpholino)methanone (16a)

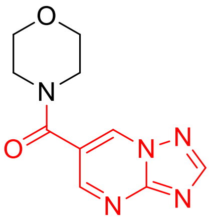

16a

Exact Mass: 233,09
Molecular Weight: 233,23
m/z: 233.09 (100.0%), 234.09 (12.7%)

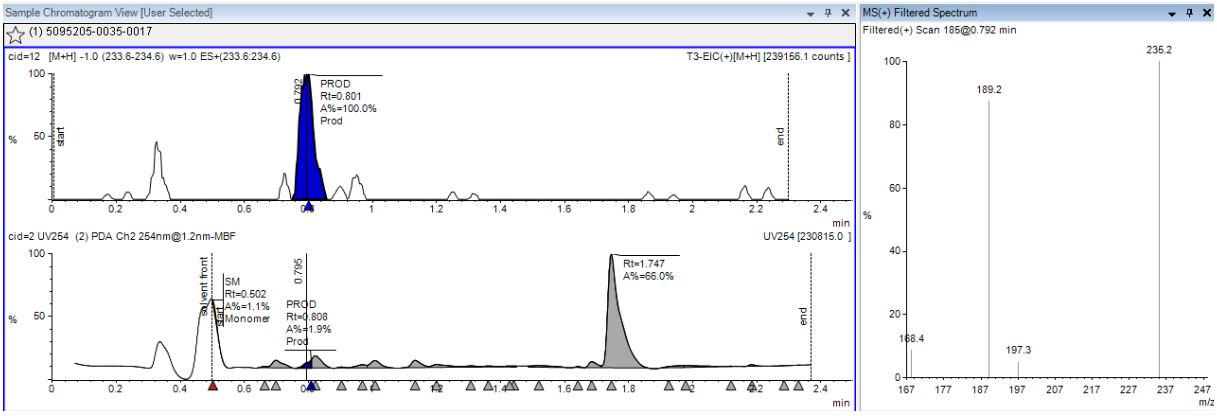

3-(3,4-Dihydro-1H-carbazol-9(2H)-yl)-1-morpholinopropan-1-one (17a)

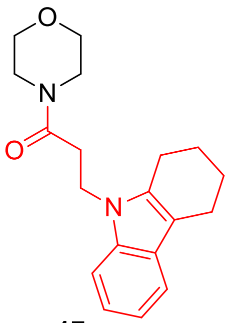

17a

Exact Mass: 312,18
Molecular Weight: 312,41
m/z: 312.18 (100.0%), 313.19 (20.9%), 314.19 (2.5%)

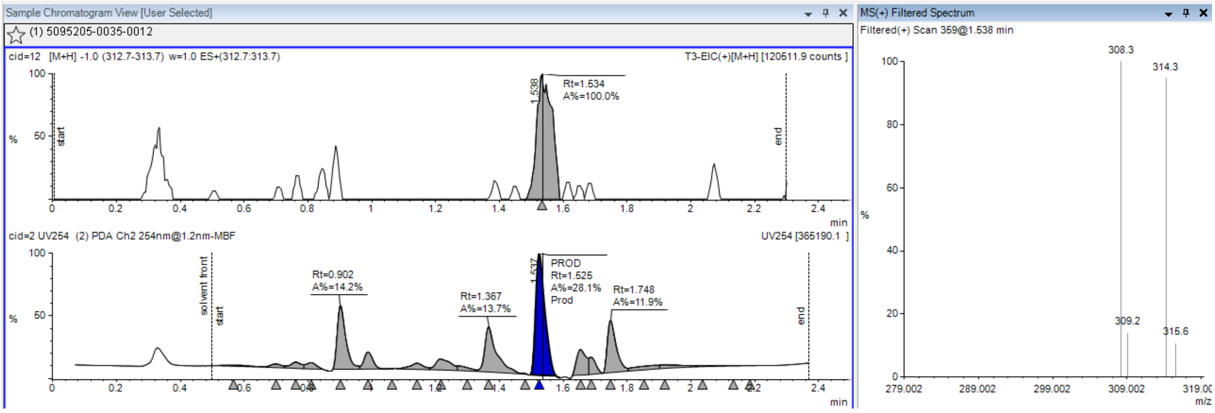

1-(3-Chlorophenyl)-6-morpholinohexane-1,6-dione (18a)

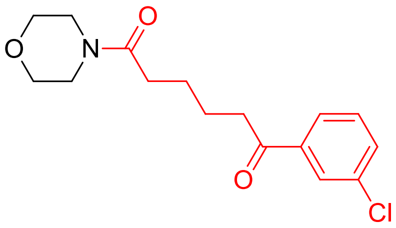

18a

Exact Mass: 309,11
Molecular Weight: 309,79
m/z: 309.11 (100.0%), 311.11 (32.0%), 310.12 (17.6%), 312.11 (5.7%), 311.12 (2.1%)

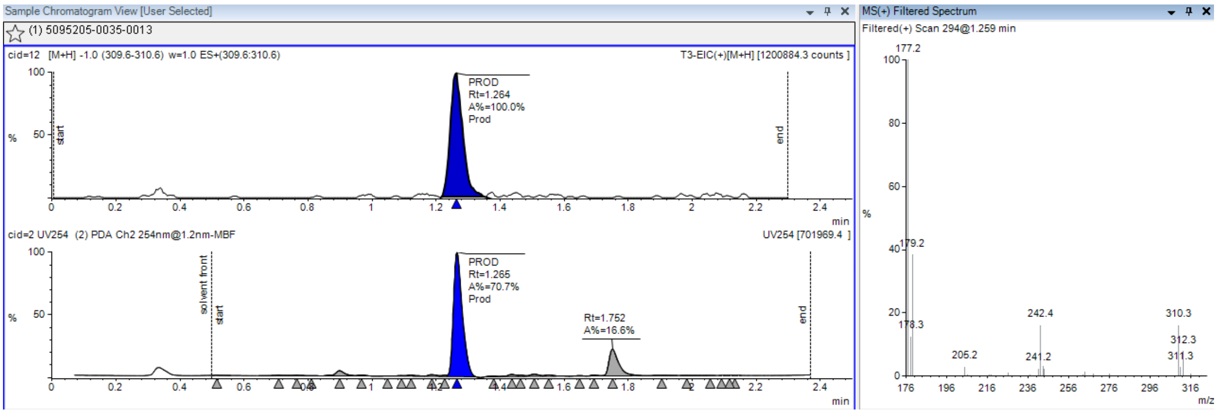

3-(4-(1H-Imidazol-1-yl)phenyl)-1-morpholinopropan-1-one (19a)

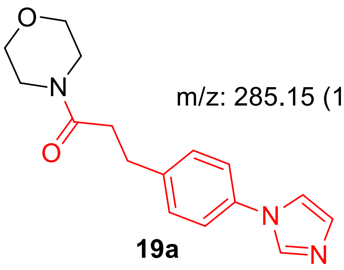

19a

Exact Mass: 285,15
Molecular Weight: 285,34
m/z: 285.15 (100.0%), 286.15 (17.6%), 287.15 (2.0%), 286.14 (1.1%)

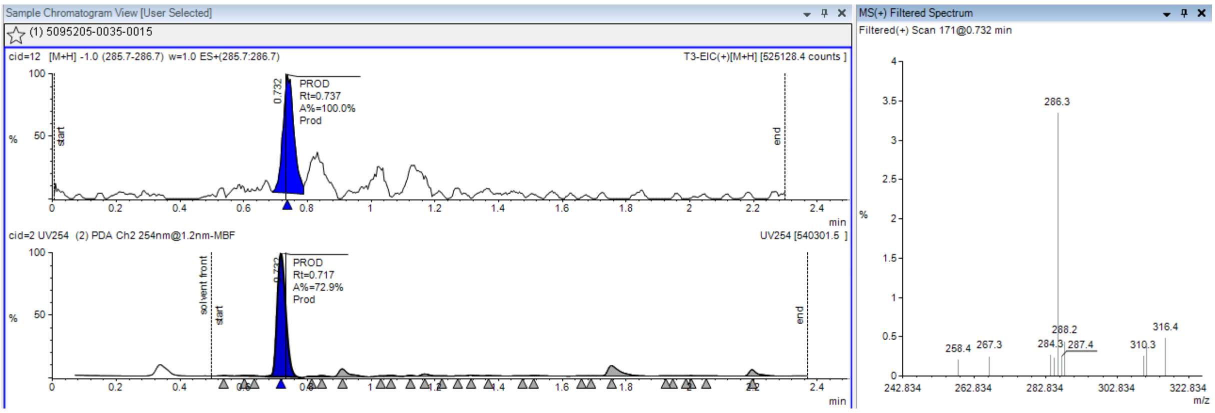

(5-Chlorothieno[3,2-b]pyridin-3-yl)(morpholino)methanone (20a)

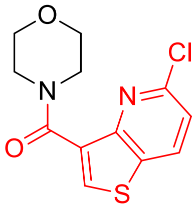

20a

Exact Mass: 282,02
Molecular Weight: 282,75
m/z: 282.02 (100.0%), 284.02 (36.6%), 283.03 (13.2%), 285.02 (5.3%), 286.02 (1.7%), 283.02 (1.5%), 284.03 (1.3%)

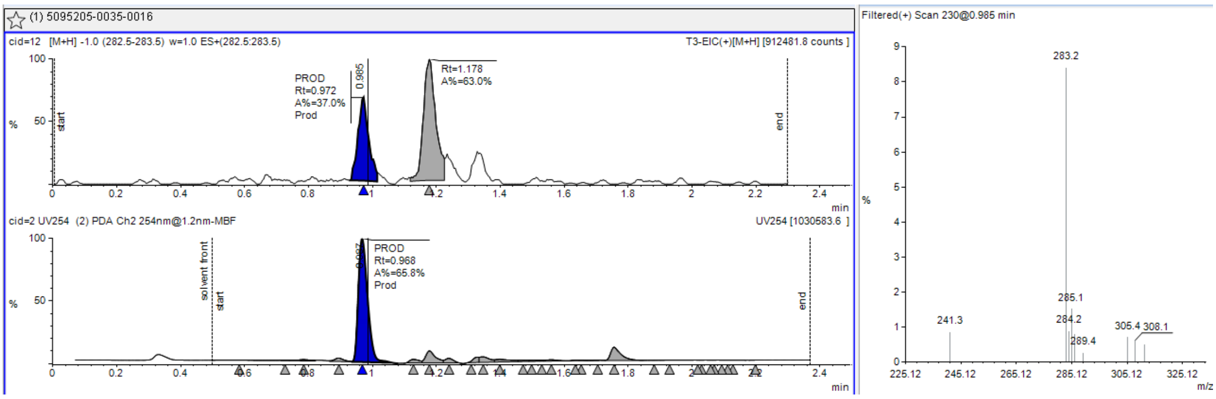

Morpholino(3-propoxypyridin-2-yl)methanone (21a)

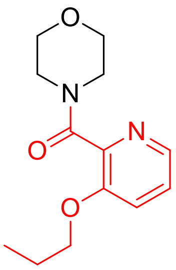

21a

Exact Mass: 250,13
Molecular Weight: 250,29
m/z: 250.13 (100.0%), 251.14 (14.4%), 252.14 (1.6%)

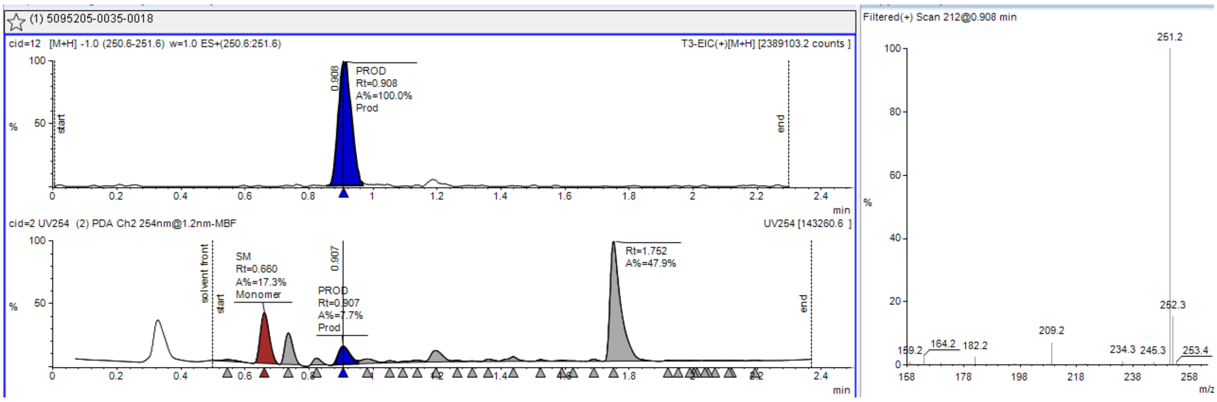

7-(2-Morpholino-2-oxoethyl)-2H-benzo[b][1,4]oxazin-3(4H)-one (22a)

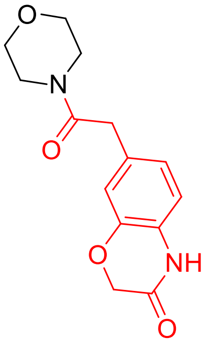

22a

Exact Mass: 276,11
Molecular Weight: 276,29
m/z: 276.11 (100.0%), 277.11 (15.9%), 278.12 (1.9%)

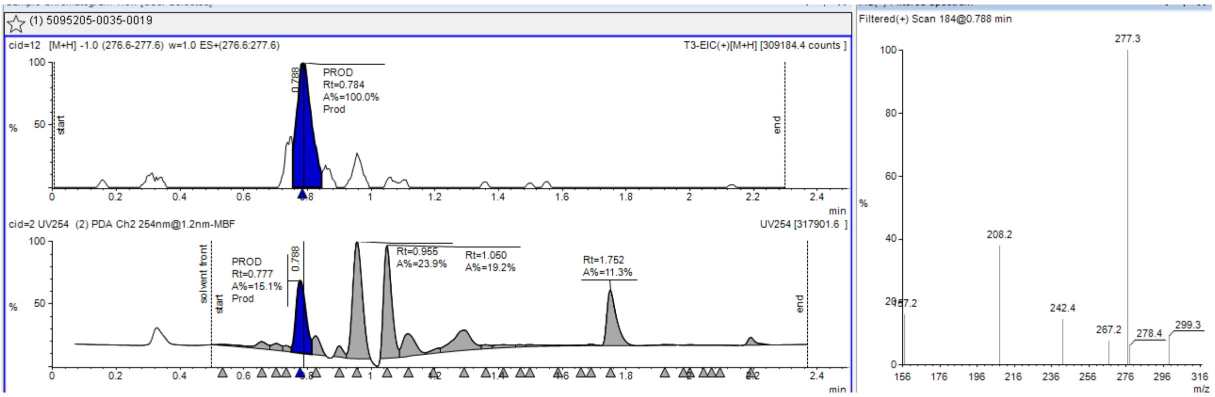

(4-(Heptyloxy)phenyl)(morpholino)methanone (23a)

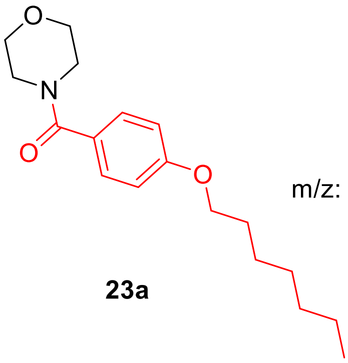

23a

Exact Mass: 305.20
Molecular Weight: 305,41
m/z: 305.20 (100.0%), 306.20 (20.0%), 307.21 (1.9%)

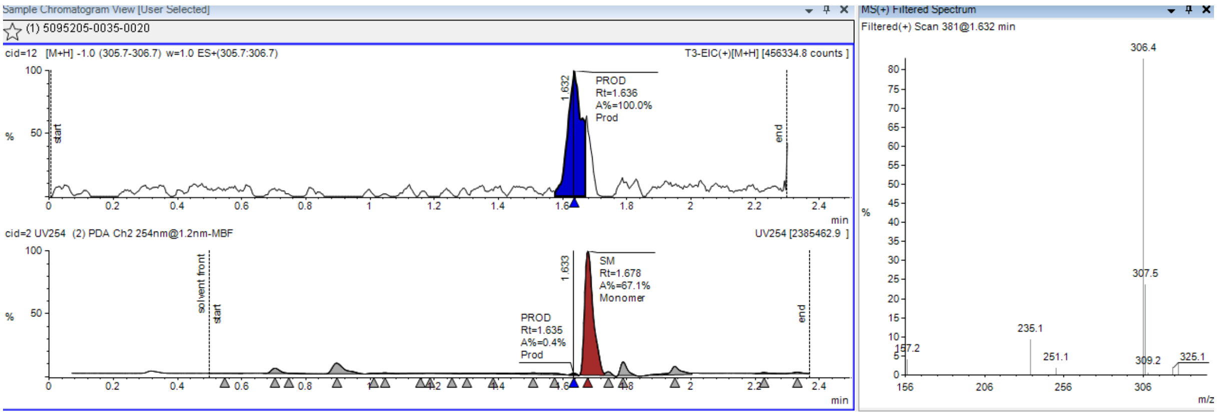

(5-(Benzo[d][1,3]dioxol-5-yl)-1H-pyrazol-3-yl)(morpholino)methanone (24a)

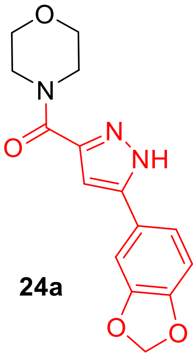

24a

Exact Mass: 301.11
Molecular Weight: 301,30
m/z: 301.11 (100.0%), 302.11 (16.5%),
303.11 (2.3%), 302.10 (1.1%)

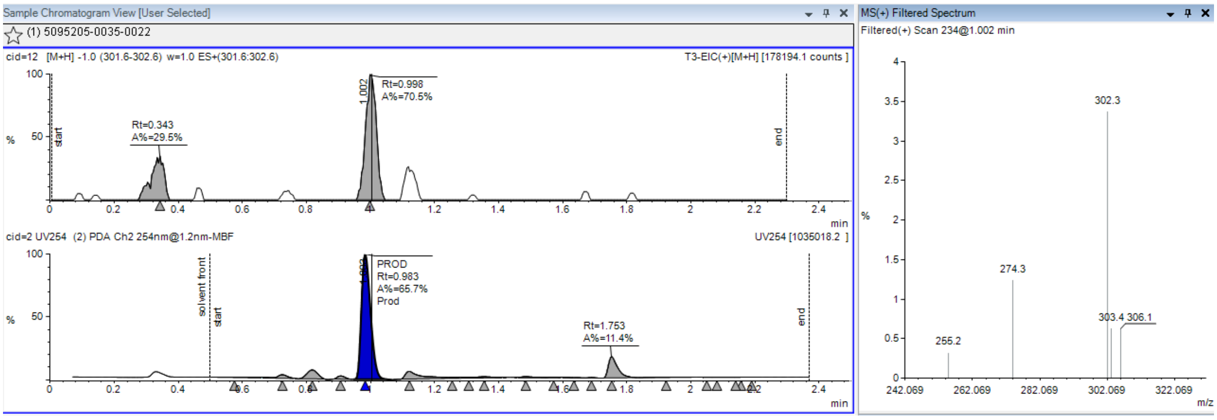

Supplement: Supplementary file 1 [file ao5c04718_si_001.pdf]
